# Understanding Clinician Perceptions of GenAI: A Mixed Methods Analysis of Clinical Documentation Tasks

**DOI:** 10.1007/s10916-025-02234-8

**Published:** 2025-08-02

**Authors:** David Fraile Navarro, A. Baki Kocaballi, Shlomo Berkovsky

**Affiliations:** 1 Centre for Health Informatics, Australian Institute of Health Innovation, Macquarie University, 75 Talavera Road, Sydney, 2113 NSW Australia; 2 Faculty of Engineering and Information Technology, University of Technology Sydney, Sydney, Australia

**Keywords:** Electronic health records, Generative artificial intelligence, Primary care, Human computer interaction

## Abstract

This mixed-methods study evaluated clinicians’ user experience (UX) with Generative AI (GenAI) in Electronic Health Record (EHR) systems across three clinical documentation tasks (Information Extraction, Summarization, and Speech-to-Text) at varying levels of user supervision (low, medium, high), focusing on workflow improvements, safety, and acceptable automation levels. Using conceptual prototyping in a usability study framework, we evaluated how incorporating GenAI into EHR could support the three documentation tasks at varying automation levels. A total of 38 clinicians interacted with the prototype and completed a questionnaire on task relevance, perceived importance, desired automation level, and EHR satisfaction. Both quantitative (descriptive statistics, Kruskal-Wallis tests, Spearman correlations) and qualitative (thematic) analyses were conducted with equal priority to explore preferences, perceived safety, and practical requirements. Clinicians showed positive reception to GenAI integration, particularly for streamlining documentation. While task relevance and importance were strongly correlated, EHR satisfaction did not significantly predict automation acceptance. Medium automation emerged as the preferred level, considered “safe with caution”. Five key themes emerged from qualitative analysis: efficiency and quality benefits; system reliability concerns; safety and medico-legal considerations; automation bias and loss of nuance; and deployment requirements including adjustable settings and oversight. While clinicians welcome GenAI-driven documentation, they prefer moderate automation to balance efficiency with clinical control. Successful integration requires addressing safety concerns, conducting real-world trials, and mitigating potential biases and medico-legal challenges. These findings suggest a cautious but optimistic path forward for AI integration in EHR systems, emphasizing the importance of maintaining clinician oversight while leveraging automation benefits.

**Supplementary Information:**

The online version contains supplementary material available at 10.1007/s10916-025-02234-8.

## Introduction

Electronic Health Records (EHRs) have fundamentally transformed healthcare documentation, promising improved efficiency and quality of care [1]. However, their implementation has introduced significant challenges that continue to impact clinical practice. Despite growing standardization efforts [2–4], clinical documentation remains heavily dependent on free-text entries. This reliance on unstructured data, combined with increasing documentation requirements, has created persistent friction in healthcare workflows [5, 6].

The burden of EHR-related tasks has become a critical issue in healthcare delivery. Clinicians report spending up to 50% of their time on documentation tasks, contributing significantly to professional dissatisfaction and burnout [6–9]. These tasks encompass manual coding, document transcription, clinical note-taking during patient encounters, and information retrieval from fragmented records [10]. Paradoxically, while EHRs were designed to streamline clinical practice, their impact on workflow efficiency [11] and clinician satisfaction remains contentious [12].

The emergence of advanced Natural Language Processing (NLP) technologies, particularly Large Language Models (LLMs) and Generative AI (GenAI), presents an unprecedented opportunity to address these documentation challenges [5, 13]. Primary care represents an ideal setting for GenAI deployment, as primary care physicians face increasing patient loads while serving as the primary curators of medical records [6]. Early implementations of AI-powered clinical scribes have demonstrated promising results in reducing documentation time and improving clinician satisfaction [7].

Recent research has systematically explored healthcare providers’ attitudes toward AI-assisted documentation [14–17]. These studies consistently identify three core tasks where AI assistance could provide substantial value: (1) information extraction from clinical texts, (2) speech-to-text conversion during consultations, and (3) summarization of clinical documents [14, 18]. While current AI technologies demonstrate technical capability in performing these tasks, significant implementation challenges remain [19, 20].

The path from technical capability to clinical implementation requires addressing multiple complex challenges. These include determining appropriate levels of automation that balance efficiency with clinical oversight, ensuring seamless integration with existing EHR systems, maintaining rigorous safety standards, and designing user experiences that support rather than disrupt clinical workflows [14, 15, 21]. Success requires careful consideration of both technical capabilities and the realities of clinical practice [22, 23]. Additionally, the evolving regulatory landscape necessitates frameworks for evaluating and approving AI-powered medical software that can adapt to rapid technological advancement.

This study aims to advance previous research by systematically evaluating how clinicians interact with and perceive various levels of GenAI automation in clinical documentation tasks. We seek to understand user requirements and provide evidence-based guidance for designing GenAI tools that effectively support clinical practice while maintaining safety and usability standards.

## Methods

We conducted a convergent mixed-methods study with equal priority given to both quantitative and qualitative components to assess clinician perspectives on AI-assisted clinical documentation. Following established HCI methodologies [24], we conducted a usability evaluation using conceptual prototyping to inform design requirements before real-world implementation. This approach allows systematic exploration of user preferences and concerns in a controlled environment, providing crucial insights that guide the development of systems suitable for clinical deployment. The study design incorporated three integrated components to provide comprehensive insights:

- An interactive, high-fidelity prototype simulating GenAI integration within a familiar EHR interface
- Structured tasks representing authentic primary care documentation workflows
- Mixed-methods evaluation combining quantitative usability metrics with qualitative feedback

### Participants

We recruited 38 practicing Australian primary care physicians through multiple recruitment channels including Macquarie University mailing lists, Australian Local Health District Networks, Royal Australian College of General Practitioners (RACGP) channels, and personal professional networks. To ensure a diverse and representative sample, we employed purposive sampling with the inclusion criterion of current clinical practice in primary care and prior experience with documenting in patient records. All participants provided written informed consent and received AU$50 compensation for their time. The study sessions were conducted fully online.

### Prototype Design and Tasks

We developed an interactive EHR prototype using iterative co-design principles, with the interface modeled after a widely-used Australian EHR system familiar to most participants. The prototype, referred to as “MagicGP” in the interface elements, underwent multiple rounds of testing to ensure realism and usability. Synthetic patient data, including complete medical histories, consultation notes, and specialist correspondence, were created by a primary care physician researcher (DFN) based on personal clinical experiences and validated by an independent clinician for authenticity and complexity. These cases were previously prototyped with clinical staff to ensure they reflected realistic documentation scenarios encountered in primary care practice.

The prototype evaluated three fundamental clinical documentation tasks identified through prior research as high-impact areas for AI assistance:

#### Information Extraction (IE)

This task simulated the common clinical scenario of processing specialist correspondence. Participants received a detailed specialist letter and were required to identify and extract clinically relevant information (diagnoses, medications, follow-up recommendations) for integration into the patient’s EHR. This task addresses a time-consuming aspect of primary care practice where important clinical information must be accurately transferred between documents.

#### Summarization (SUM)

Participants were tasked with generating concise summaries of patient health records for various clinical purposes. The task included creating condition-specific summaries (e.g., diabetes management history) and comprehensive patient overviews. This functionality addresses the challenge of quickly synthesizing large volumes of historical clinical data.

#### Speech-to-Text (S2T)

This task simulated the conversion of patient-clinician dialogue into structured clinical notes. Participants reviewed simulated consultation recordings and evaluated the system’s ability to generate accurate, clinically relevant documentation from verbal exchanges. This addresses the growing interest in ambient clinical intelligence and voice-enabled documentation.

For each task, we implemented three distinct automation levels based on established human-automation interaction frameworks [25, 26]. These levels were carefully calibrated to represent meaningful differences in system autonomy and user control (Table 1).

**Table 1:** Automation levels and interaction design for each clinical documentation task

Task	Low Automation	Medium Automation	High Automation
IE	System highlights potential entities; user manually selects each item; individual review required for each selection; explicit confirmation needed	System extracts and presents all entities; user selects relevant items from list; optional bulk review; single confirmation step	System automatically extracts and pre-selects all entities for inclusion; user can deselect items; optional review; streamlined confirmation
SUM	Generates basic summary of input document (e.g., specialist letter) with minimal interpretation	Creates targeted summary for specific conditions using full EHR context; allows customization of focus areas	Produces comprehensive, intelligent summary of entire patient record with key insights highlighted
S2T	Provides verbatim transcription only; user must manually edit and structure for clinical notes	Generates structured summary of selected conversation segments; allows user to choose relevant portions	Automatically creates complete, structured clinical note from entire consultation; includes decision support

**Fig. 1:**
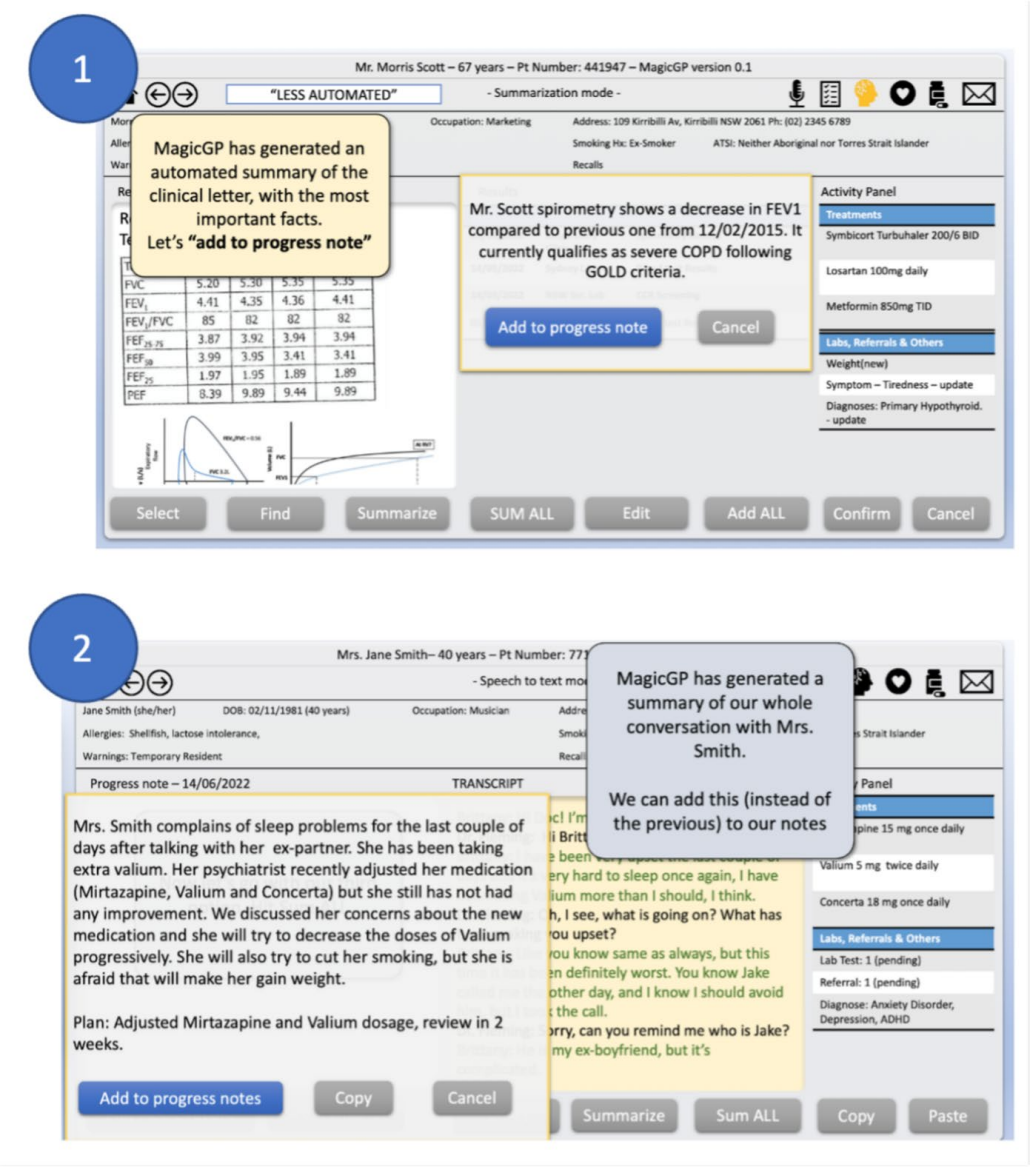
Example Screens from Summarization and Speech to Text Tasks. (1) Summarization scenario, with the Low automation level. The user received a specialist letter and asked to produce a summary of one element to review or add to notes. (2) Speech-to-Text scenario, with the High automation level. The system created a full summary of a conversation between a doctor and a patient, for the doctor to add to the patient records with a single confirmation step

The prototype interface incorporated evidence-based design principles including progressive disclosure, clear visual feedback, and reversible actions. Navigation guides and contextual help were integrated to support participants unfamiliar with specific features while avoiding interference for experienced users. Figure 1 provides examples of Summarization and S2T tasks (full-experiment screen-captures are available in Supplementary File 1, see Appendix A).

### Study Procedure

Each participant completed a structured 45-60 minute session following a standardized protocol:

1. **Introduction and Tutorial** (10 minutes): Participants received an overview of the study objectives and completed an interactive tutorial demonstrating the three automation levels across all tasks.
2. **Task Completion** (25 minutes): Participants completed all nine task-automation combinations (3 tasks $\times$ 3 automation levels) in randomized order to control for learning and fatigue effects. Each task used different patient scenarios to prevent repetition. The majority of participants (n=32) completed the tasks within the expected timeframe, with a mean completion time of 8.43 minutes. Overall completion times ranged from 2.17 to 44.75 minutes (median = 6.86 minutes), with outliers likely reflecting participants who took breaks during the session.
3. **Immediate Evaluation** (15 minutes): After each task, participants completed structured questionnaires assessing task relevance, perceived importance, safety concerns, and automation preferences using 7-point Likert scales.
4. **Final Assessment** (10 minutes): Participants completed a comprehensive questionnaire covering demographic information, EHR experience, overall impressions, and open-ended feedback on their experience with the prototype features, safety concerns, and implementation considerations.

Data were collected using Google Forms, which captured both structured questionnaire responses (e.g., 7-point Likert scales) and free-text feedback. The form responses were exported in spreadsheet format and processed locally using Python statistical analysis packages for quantitative analysis. This approach ensured data quality through built-in validation in Google Forms while maintaining participant privacy through secure data handling procedures. The complete questionnaire is available in Supplementary File 2 (see Appendix B).

### Data Collection and Analysis

#### Quantitative Analysis

We employed a comprehensive statistical approach appropriate for our mixed-methods design and non-normally distributed ordinal data:

- **Descriptive Statistics**: Calculated means, standard deviations, and medians for all quantitative measures.
- **Group Comparisons**: Used Kruskal-Wallis tests [12] to examine differences in ratings based on automation preferences and perceived workflow improvements. When significant differences were detected (p < 0.05), we conducted post-hoc pairwise comparisons using Dunn’s test [27] with Bonferroni correction.
- **Repeated Measures**: Applied Friedman’s test to analyze safety perceptions across automation levels, accounting for within-subject design. Significant results were followed by Wilcoxon signed-rank tests [14] for pairwise comparisons.
- **Effect Sizes**: Calculated eta-squared ($\eta ^2$) for Kruskal-Wallis tests and Kendall’s W for Friedman tests to assess practical significance.
- **Correlation Analysis**: Used Spearman’s rank correlation to examine relationships between task relevance, importance, daily use intentions, and EHR satisfaction.

Complete analysis scripts are available in Supplementary File 3 (see Appendix C).

#### Qualitative Analysis

Open-ended responses underwent rigorous thematic analysis following Braun and Clarke’s [28] six-phase framework:

1. Data familiarization through repeated reading
2. Initial code generation by two independent researchers (DFN, who has a background as a GP with experience in qualitative research and HCI methods, and ABK, with expertise in computer science, HCI and qualitative methods)
3. Searching for themes across the dataset
4. Reviewing themes against coded extracts and entire dataset
5. Defining and naming themes through collaborative discussion
6. Producing the final thematic map with representative quotes

The integration of qualitative and quantitative findings was achieved through joint displays and triangulation of results, with both components given equal weight in the interpretation.

### Researcher Reflexivity

The research team brought complementary perspectives to the analysis. The first author (DFN) is a practicing general practitioner with experience in qualitative research and HCI methods, providing clinical insight and understanding of documentation workflows. The second author (ABK) has expertise in computer science, HCI, and qualitative methods, offering technical and methodological perspectives. The senior author (SB) provided oversight from a health informatics perspective. This multidisciplinary approach enabled balanced interpretation of both clinical and technical aspects of the findings.

## Results

### Participant Characteristics

The study sample comprised 38 primary care physicians representing diverse practice contexts and experience levels. Table 2 presents detailed participant characteristics. The majority (97%) worked exclusively in primary care settings, with 43% also providing telehealth services. Experience levels varied substantially, from recent graduates to senior practitioners with over 20 years of experience. Urban practitioners dominated the sample (89%), though 11% worked across both urban and rural settings.

**Table 2:** Participant demographics and practice characteristics (N=38)

Characteristic	n	Percentage
Primary Practice Setting*		
Primary care only	37	97%
Primary care with telehealth	16	43%
Years in Practice		
Less than 5 years	13	35%
5-10 years	12	32%
10-20 years	10	27%
More than 20 years	3	5%
Practice Location		
Urban only	34	89%
Urban and rural	4	11%
EHR Usage		
Every consultation	33	86%
Most consultations	5	14%
Current EHR System		
Medical Director	22	58%
Best Practice	11	29%
Other	5	13%

*Participants could select multiple options

Participants reported moderate satisfaction with current EHR systems (median = 4, interquartile range (IQR) = 3-5 on a 7-point scale) (Table 3). Qualitative feedback revealed a complex relationship with existing systems: while participants acknowledged benefits such as legibility and information sharing, they frequently cited interface complexity, excessive clicking, and time-consuming data entry as major pain points.

**Table 3:** Descriptive statistics across tasks

	Information Extraction (IE)		Summarization (SUM)		Speech to text (S2T)	
Question/Task	Mean	Std Dev	Mean	Std Dev	Mean	Std Dev
*How does the above scenario match your day-to-day practice?* (TaskMatch)	5.82	1.29	5.79	1.12	5.13	1.55
*How important do you find addressing the scenario?* (Importance)	5.55	1.16	5.71	0.90	5.21	1.44
*Would you consider using it in your day-to-day practice?* (UseDayToDay)	5.05	1.47	5.08	1.42	4.82	1.71
*How satisfied are you with current EHR systems?* (EHRsatisfaction)	4.14	0.89	4.14	0.89	4.14	0.89
*Could automation improve workflow?* (ImproveWorkflow)	**Count**	**%**	**Count**	**%**	**Count**	**%**
Yes	20	54%	22	59%	21	57%
Maybe	15	40%	10	27%	12	32%
No	3	8%	6	16%	5	14%
*Preferred automation level*	**Count %**		**Count %**		**Count %**	
More automated	20	53%	17	45%	12	32%
Depends on context	7	18%	9	24%	11	29%
Completely automated	6	16%	7	18%	8	21%
Less automated	5	13%	5	13%	7	18%

## Documentation Tasks

### Task Relevance and Perceived Value

**Fig. 2:**
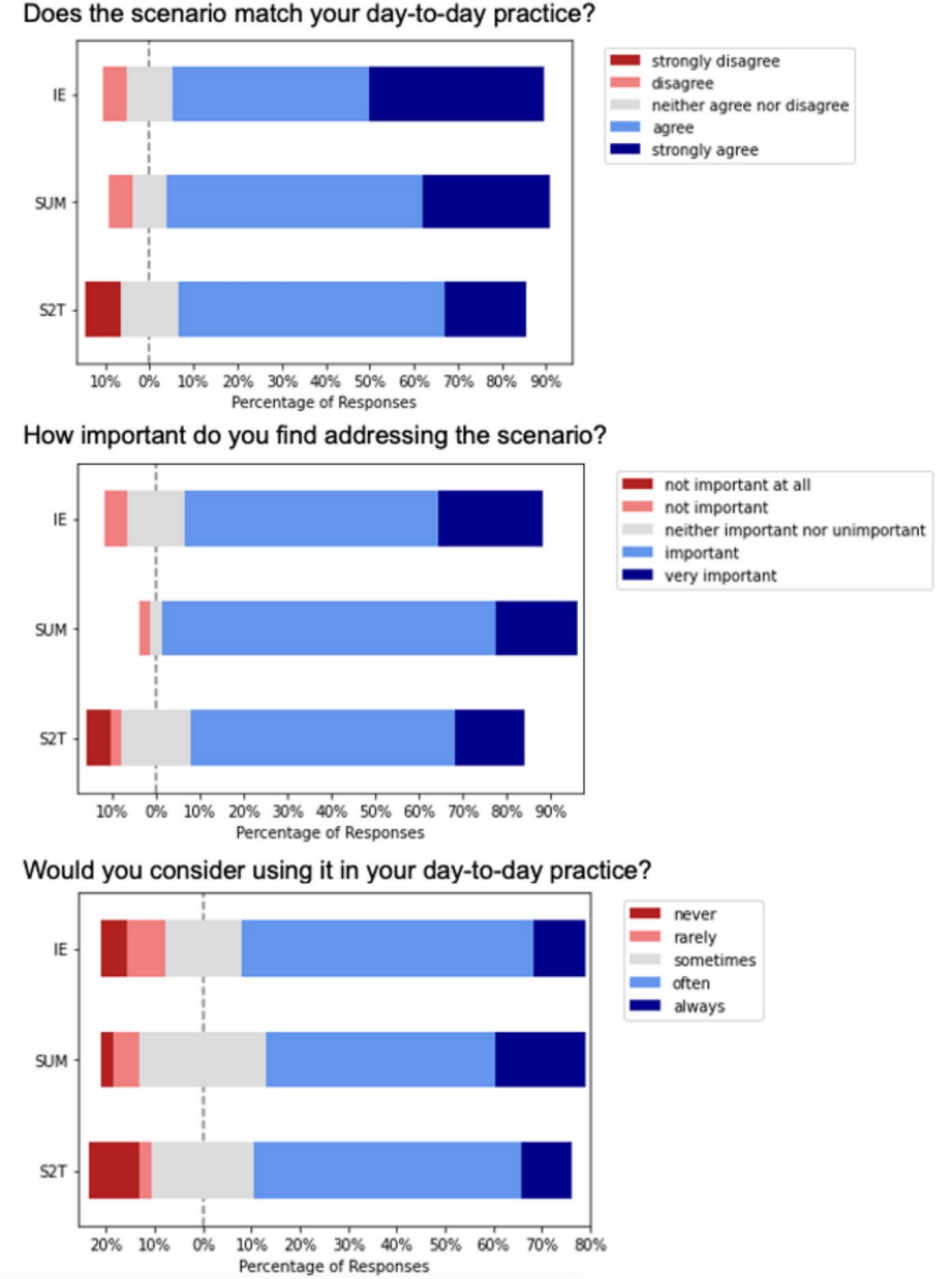
Scenario relevance and perceived benefits of automation

All three documentation tasks demonstrated high perceived relevance to clinical practice. Information Extraction and Summarization tasks received the highest relevance ratings (median = 6), while Speech-to-Text showed slightly lower but still substantial relevance (median = 5). The strong correlation between task relevance and importance across all tasks (Spearman’s $\rho$ range: 0.75-0.82, all $p < 0.001$) suggests participants recognized both the frequency and significance of these documentation activities in their practice (Fig. 2) (Table 4).

**Table 4:** Descriptive statistics for task evaluation metrics

Metric	Information Extraction		Summarization		Speech-to-Text	
	Mean (SD)	Median [IQR]	Mean (SD)	Median [IQR]	Mean (SD)	Median [IQR]
Task Relevance	5.82 (1.29)	6 [5-7]	5.79 (1.12)	6 [5-7]	5.13 (1.55)	5 [4-6]
Importance	5.55 (1.16)	6 [5-6]	5.71 (0.90)	6 [5-6]	5.21 (1.44)	5 [4-6]
Daily Use Intent	5.05 (1.47)	5 [4-6]	5.08 (1.42)	5 [4-6]	4.82 (1.71)	5 [3-6]

When asked to identify the most important task, responses were distributed across all options: 41% considered all tasks equally important, 24% prioritized Speech-to-Text, 24% selected Information Extraction, and 11% chose Summarization. This distribution suggests that documentation needs vary significantly across clinical contexts and individual practice patterns. Participants who believed GenAI could improve clinical workflows rated all tasks as more relevant and important, reflecting different levels of technology receptiveness within the clinical community.

### Automation Preferences and Safety Perceptions

**Fig. 3:**
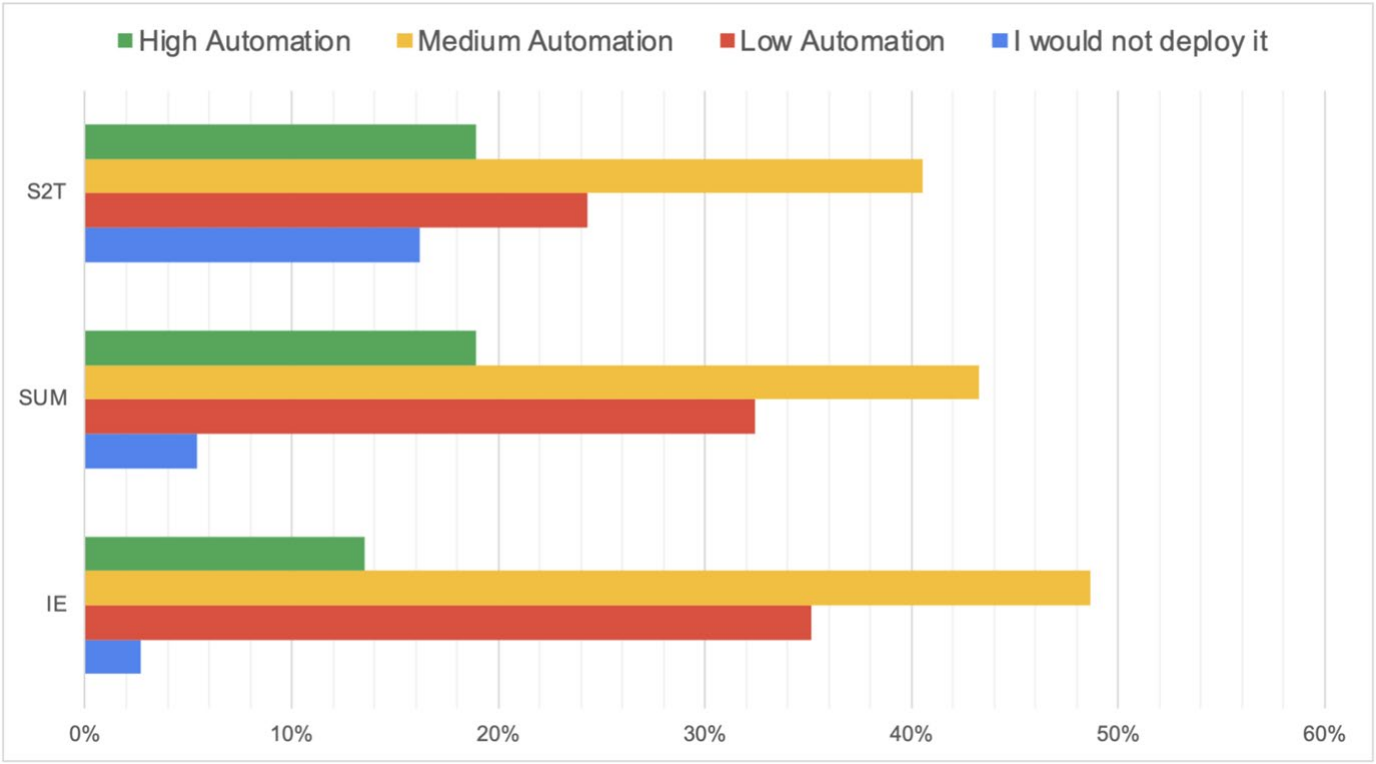
Preferred automation levels by task type. Medium automation consistently emerged as the preferred option, though Speech-to-Text showed the highest acceptance of high automation and the highest rejection of automation overall

Analysis of automation preferences revealed a clear pattern favoring moderate levels of system autonomy. Medium automation emerged as the most preferred option across all tasks, though the strength of this preference varied by task type (Fig. 3).

The relationship between automation level and perceived safety followed a consistent inverse pattern across all tasks. Friedman tests confirmed significant differences in safety perceptions across automation levels for all tasks ($\chi ^2$ = 28.4, $p < 0.001$ for IE; $\chi ^2$ = 31.2, $p < 0.001$ for SUM; $\chi ^2$ = 26.8, $p < 0.001$ for S2T). Post-hoc analyses revealed that each increase in automation level corresponded to a significant decrease in perceived safety (all pairwise comparisons $p < 0.01$).

**Fig. 4:**
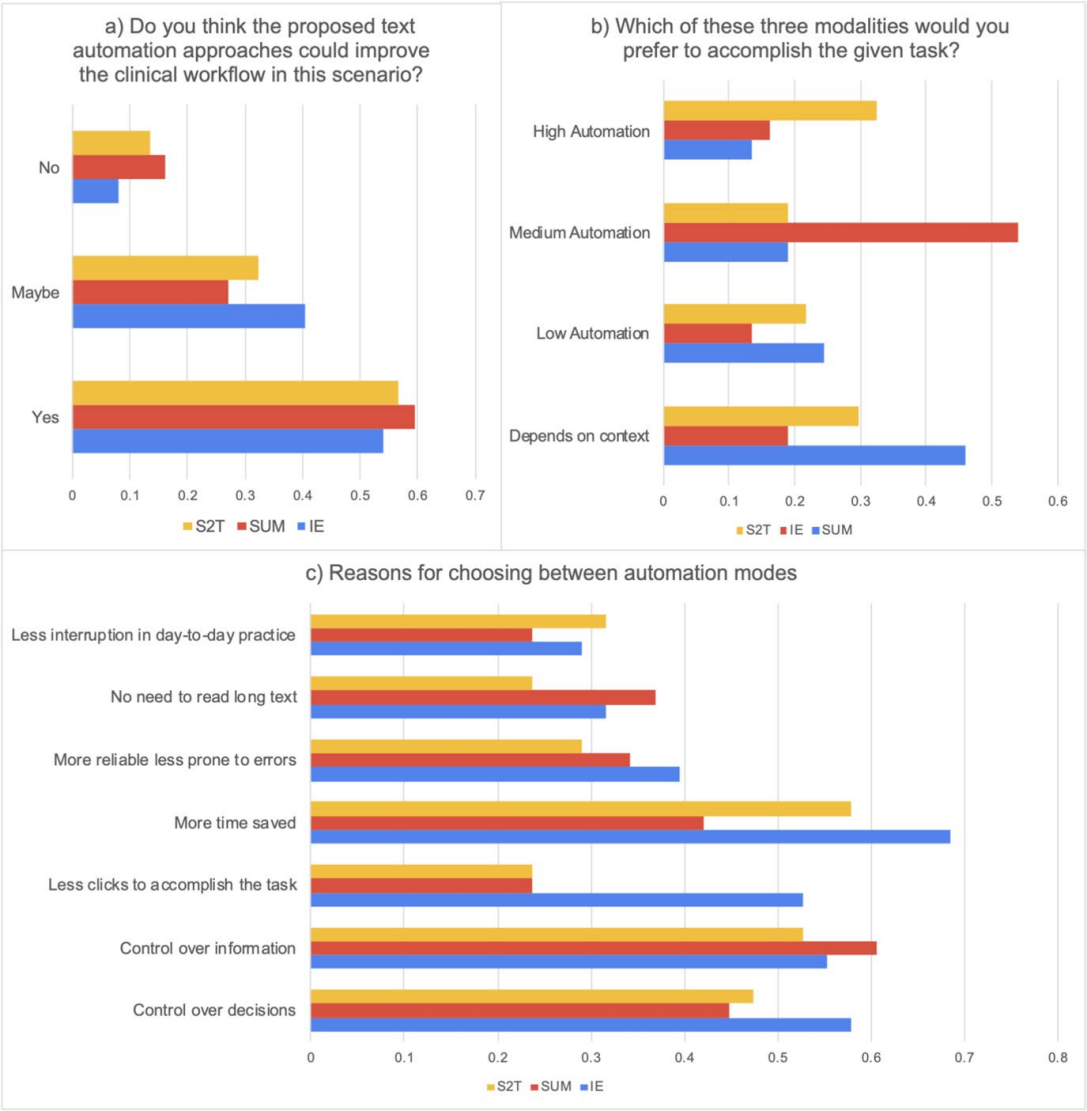
How different scenarios and automation modes were considered

**Fig. 5:**
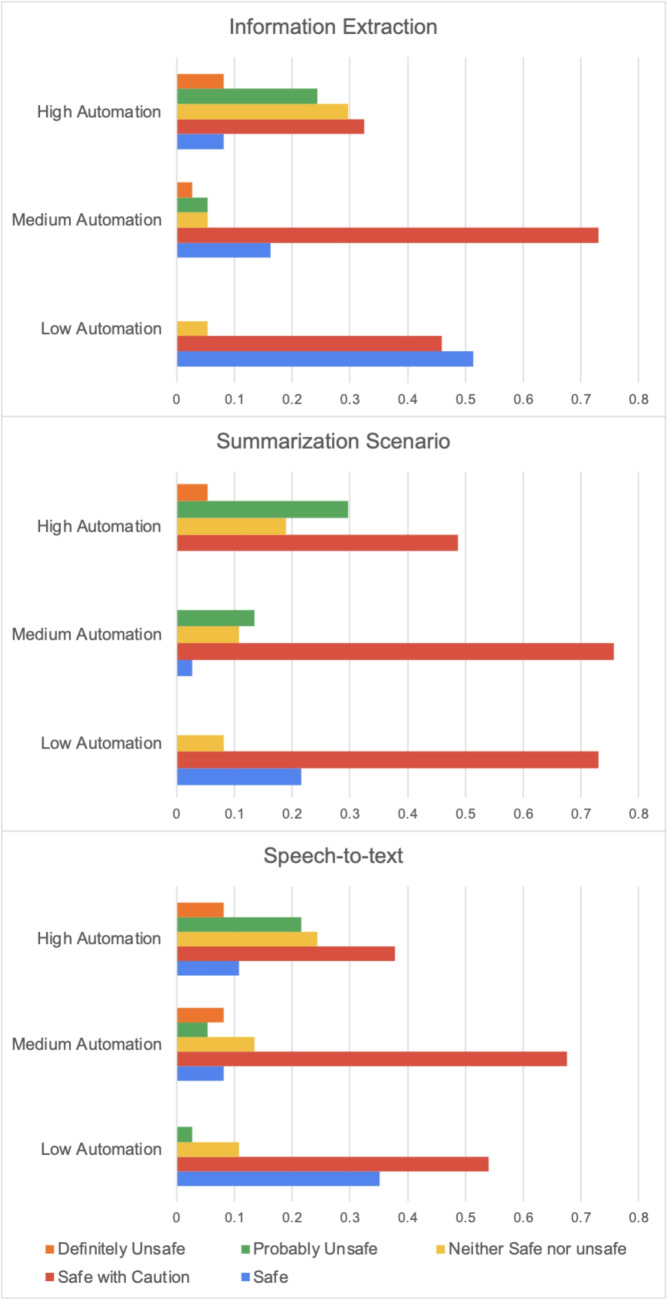
Perceived safety of each level of automation by scenario

Effect sizes for these differences were substantial, with Kendall’s W values ranging from 0.25 to 0.27, indicating moderate to strong agreement among participants regarding safety concerns. Notably, while low automation was consistently rated as “Safe” or “Safe with Caution” by over 70% of participants, high automation received “Probably Unsafe” or “Definitely Unsafe” ratings from 30-35% of participants across tasks (Figs. 4 and 5).

### Factors Influencing Acceptance

Participants who believed GenAI could improve clinical workflows showed significantly higher ratings across multiple metrics. For Speech-to-Text, this effect was particularly pronounced: those anticipating workflow improvements rated task relevance significantly higher (median = 6.0) compared to those uncertain about benefits (median = 4.5, U = 87.5, $p = 0.018$, r = 0.38).

Surprisingly, correlation analyses revealed no significant relationship between current EHR satisfaction and GenAI acceptance metrics (all $p> 0.05$). This independence suggests that enthusiasm for AI-assisted documentation transcends satisfaction with existing systems, potentially representing a distinct dimension of technology acceptance in clinical practice.

### Qualitative Analysis

A thematic analysis of the free-text responses yielded five themes surrounding GenAI automation in EHRs, capturing participants’ perspectives on efficiency and quality improvements, reliability and usefulness, safety, automation limitations, and deployment requirements (Table 5). Participants highlighted the potential for timesaving, better record keeping, and improved information extraction/retrieval. While many participants perceived GenAI as promising, they stressed the need for adequate trialing to ensure reliability and raised concerns about automation bias and the risk that clinicians trust system outputs uncritically.

**Table 5:** Themes and subthemes with supporting quotes from participants

Theme	Subtheme	Supporting quotes
Efficiency and quality improvements	Timesaving	*P6: “Makes the process of converting free text into retrievable information much easier.”*
	Better record keeping	*P15: “The text automation will save time and increase accuracy. I think text automation can be safer than copy-pasting”*
	Improved information extraction and retrieval	*P18: “It is brilliant - it extracts the important information and pulls it together in the Right panel and also imports it into the clinical file from the specialists’ letter - something that is very time-consuming in routine General Practice”*
	Better patient representation and engagement	*P17: “I think it can help you focus more on doctor-patient relation and improve it by making the patient feel more listened”*
Reliability and usefulness	System needs adequate trialing	*P7: “I would need to try it in my clinical practice but theoretically, it has promise”*
	Effortful automation supervision	*P30: “Good to keep an updated record, but multiple steps are time-consuming”*
	Reliance on record quality	*P14: “The quality of the automated extracted data is only as good as the correctness of the medical record and user inputs. Lots of clinicians still don’t appreciate the importance of data hygiene when keeping medical records.”*
	Manual record preference	*P10: “I want to write my own notes.”*
Safety	Clinical oversight required	*P1: “I think that safety will also be influenced by checks and balances implemented by the practitioner.”*
	Medico-legal issues	*P33: “How would it work with consent with the consult essentially being recorded, there are lots of conversations that occur during a consult that aren’t documented or don’t need to be.”*
	Retain control over the record	*P6: “I still want to own and control the synthesis of records”*
	Automation/safety trade-off	*P11: “The more automated an option is, the less input is required which leads to possibility of error”*
Automation Limitations	Automation bias	*P33: “Completely automated approach may bring about clinician complacency in the long term”*
	Misinterpretation and information loss	*P21: “Sometimes just the text would not capture the non-verbal communication that may be really important in the consult”*
	Information clogging	*P23: “Need to check what has been summarized, don’t want to include too much verbatim of the patient or notes become clunky”*
Deployment requirements	Personalization and extraction styles	*P29: “Less is more sometimes. I find dot points of important results/things easier to process.”*
	Interaction checkpoints	*P17: “I would ask a question before adding the information to the note to make sure the professional reads the results.”*
	Adjustable automation	*P2: “Less automation would be useful, more control over the specifics, e.g. I write ’results’ and a ’specific parameter’ to be transferred to the clinical notes.”*
	Other desired features	*P30: “I’m interested in how it handles intangibles, such low energy for investigation, also mental health.”*

## Discussion

Our mixed-methods study provides critical insights into clinician perspectives on GenAI integration in clinical documentation, revealing a nuanced landscape of cautious optimism tempered by legitimate concerns about safety, control, and clinical autonomy. The clear preference for medium automation levels across all documentation tasks represents a pivotal finding that challenges assumptions about the optimal degree of AI assistance in healthcare.

This paper builds upon earlier works exploring the attitudes of primary care doctors towards text automation [9]. Our findings align with previous research, indicating that clinicians are generally receptive to implementing GenAI to streamline their workflow and improve efficiency. By reducing the administrative burden and saving time on documentation tasks, AI may provide an opportunity for doctors to reconnect with their patients by allowing more time for direct patient interaction and care [29], simultaneously enhancing the quality and efficiency of clinical documentation [30].

Recent work demonstrated initial deployment of GenAI into clinical settings. For example, ambient AI was tasked with clinical note generation achieving initial, promising results [7]; GenAI chatbot showed clinical reasoning capability on par with clinicians [31]; or, GenAI deployed to evaluate stroke management adherence to guidelines reaching agreement levels of experts [32]. These studies show the potential to integrate GenAI in tasks going beyond clinical documentation and pave the way to a stream of research on improving other elements of the clinical encounter.

### Theoretical Implications for Human-AI Collaboration

The consistent preference for medium automation aligns remarkably with established models of human-automation interaction. Parasuraman et al.’s [25] framework suggests that intermediate automation levels often provide optimal balance between workload reduction and maintenance of situation awareness. Our findings extend this framework to the clinical documentation context, where the stakes of maintaining awareness are particularly high.

The preference distribution—42% for medium automation in Information Extraction, 32% in Summarization, and 31% in Speech-to-Text—reflects what we term “calibrated trust” in AI systems. This pattern suggests clinicians seek a collaborative relationship with AI rather than replacement or minimal assistance. The significantly higher safety ratings for medium automation (typically rated “Safe with Caution” by 70-80% of participants) compared to high automation (rated “Probably Unsafe” by 30-35%) provides empirical support for this interpretation.

Our findings also contribute to understanding the “automation paradox” in healthcare: while participants acknowledged that higher automation could maximize efficiency, they simultaneously recognized that it might compromise their ability to maintain clinical oversight. This sophisticated understanding challenges simplistic narratives about resistance to technology and instead reveals thoughtful consideration of human-AI collaboration dynamics.

### Addressing the Implementation Challenge

Our findings highlight several critical requirements for successful GenAI implementation in clinical settings:

#### Flexible Automation Architecture

The strong preference for adjustable automation levels suggests that one-size-fits-all approaches will likely fail. Successful systems must allow clinicians to dynamically adjust automation levels based on case complexity, time constraints, and personal comfort. This requirement challenges current AI system design paradigms that typically offer fixed levels of assistance. In this context, flexible automation and moderate levels of oversight are crucial for controlling and mitigating the risk of biases [19].

#### Transparent Clinical Oversight

Participants’ emphasis on maintaining clinical control reflects not just personal preference but professional responsibility. Systems must provide clear mechanisms for healthcare professionals to understand key aspects of AI operations—such as the rationale behind specific outputs, the underlying data sources, and the algorithm’s decision-making logic—rather than requiring complete understanding of all AI decisions. This transparency requirement extends beyond simple explainability to include practical tools for clinical oversight that allow clinicians to validate and modify AI-generated content while maintaining ultimate responsibility for documentation accuracy.

#### Robust Testing and Validation

The repeated emphasis on adequate trialing before deployment reflects clinicians’ understanding of the stakes involved in clinical documentation. Participants wanted evidence of system performance in real-world clinical settings, not just laboratory benchmarks. This suggests that implementation strategies must include extensive pilot testing with clear metrics for safety and effectiveness. GenAI systems must be calibrated to handle diverse populations and account for local nuances, as well as mitigate biases in training data [9, 15].

#### Addressing Medico-Legal Concerns

The emergence of liability and consent issues as major themes indicates that technical solutions alone are insufficient. Successful implementation requires clear policy frameworks addressing responsibility for AI-generated content, patient consent for voice recording, and integration with existing medico-legal structures. These frameworks must be developed collaboratively with clinical, legal, and regulatory stakeholders.

#### Integration with Patient-Centered Care

An important consideration for GenAI implementation is how it facilitates patient access to their own health records. In Australia, the My Health Record system provides patients with digital access to their health information, including discharge summaries, specialist letters, and clinical documents uploaded by healthcare providers [33]. With over 90% of Australians having a My Health Record [33], clinicians are increasingly aware that patients can view the documentation they create. This transparency adds further challenges on top of clinicians’ perspectives on AI-assisted documentation as the user and consumer of these automated clinical notes is no longer clinicians only, but also patients which may have a different set of requirements and views on what should be recorded in EHRs

### Implications for GenAI Design and Deployment

Our findings suggest four design principles for clinical GenAI systems, as outlined in Table 6.

**Table 6:** Design principles for clinical GenAI systems

Design Principle	Description
Graduated Autonomy	Systems should offer seamless transitions between automation levels within workflows, allowing clinicians to increase or decrease assistance as needed.
Clinical Context Preservation	Documentation systems must maintain the narrative and contextual elements that clinicians value, not just extract discrete data points.
Active Collaboration	Rather than passive acceptance of AI suggestions, systems should support active collaboration where clinicians can guide and refine AI outputs.
Workload-Aware Adaptation	Systems might adjust their default automation level based on clinical workload, time of day, or case complexity, while always allowing manual override.

### Strengths and Limitations

Our study has several strengths. To the best of our knowledge, this is the first work studying the implementation of LLM-based approaches in EHR, exploring multiple text-processing tasks and automation levels. These addressed day-to-day problems faced by primary care doctors in their practice, going beyond synthetic NLP benchmarks and hypothetical use cases. Furthermore, the qualitative clinician input, coded thematically and intertwined with quantitative analyses, offers invaluable insight for future research and practical EHR management system development. The sample size of 38 participants aligns with established guidelines for quantitative usability studies in HCI [34, 35], which recommend 40 participants for achieving a 15% margin of error with 95% confidence [35].

Several limitations warrant consideration when interpreting our findings:

First, the relatively small sample size, albeit sufficient for usability studies, and focus on Australian primary care physicians may limit the generalizability of our findings. However, it should be noted that the Australian healthcare system combines elements of both UK-style GP-based care and American-style healthcare delivery with private specialists and reimbursement schemes similar to Medicare, potentially making these insights relevant to multiple healthcare contexts. Nevertheless, rural practitioners, specialists, and clinicians in other healthcare systems may have different perspectives and requirements. The described tasks however are fruit of primary care knowledge and experiences that are likely applicable to multiple contexts and health systems (reviewing letters from specialists, transcribing conversations or summarizing content from the health record, thus our findings are likely to resonate for many clinicians, health systems and different environments.

Second, our usability study using prototype-based evaluation and synthetic patient data represents an essential preliminary step in the health technology development pipeline. While synthetic data cannot capture all nuances of real patient cases, our validation showed that participants rated the scenarios as highly representative of their day-to-day practice (mean ratings above 5 on a 7-point scale across all tasks), suggesting the synthetic cases successfully captured authentic clinical documentation challenges. This approach follows established HCI best practices for understanding user requirements before system implementation. This foundational usability research is a prerequisite for developing systems that will be both acceptable to clinicians and effective in practice.

Third, the cross-sectional design captures only initial impressions. Longitudinal research is essential to understand how perceptions and usage patterns evolve with extended exposure to GenAI systems.

Fourth, our study focused on subjective perceptions rather than objective outcomes. Future research should examine actual documentation quality, time savings, and clinical outcomes associated with different automation levels.

Finally, while our mixed-methods approach provides rich insights, the study would benefit from additional objective measures of usability and performance in actual clinical settings.

## Conclusion

Our findings challenge both techno-optimist visions of fully automated documentation and techno-pessimist fears of clinician resistance. Instead, they reveal sophisticated clinical reasoning about the appropriate role of AI in healthcare. Clinicians seek tools that enhance rather than replace their expertise, that save time without sacrificing quality, and that respect the complexity of clinical communication.

For healthcare organizations and technology developers, these findings provide clear guidance: successful GenAI implementation requires flexible, transparent systems that support rather than supplant clinical judgment. The path forward involves not just technical innovation but careful attention to clinical workflows, professional values, and the fundamental goal of improving patient care.

As healthcare continues its digital transformation, our study suggests that the most successful innovations will be those that recognize clinicians not as obstacles to automation but as essential partners in designing the future of medical documentation. By embracing this collaborative vision, we can harness the power of GenAI while preserving the human elements that remain central to effective healthcare delivery.

## Supplementary Information

Below is the link to the electronic supplementary material.

Supplementary file 1 (pdf 3372 KB)

Supplementary file 2 (pdf 123 KB)

Supplementary file 3 (pdf 143 KB)

## Data Availability

No datasets were generated or analysed during the current study.

## Appendix A: Supplementary File 1: Full Experiment Screens

This supplementary file contains complete screenshots and interface elements for all three tasks (Information Extraction, Summarization, and Speech-to-Text) across the three automation levels (Low, Medium, High). The detailed interface views demonstrate the step-by-step interaction flow and visual design elements that participants encountered during the prototype evaluation.

## Appendix B: Supplementary File 2: Complete Questionnaire

This supplementary file provides the complete questionnaire used in the study, including all questions evaluating task relevance, perceived importance, opinions regarding automation and safety, and demographic information. The questionnaire encompasses both structured rating scales and open-ended response fields that enabled comprehensive data collection.

## Appendix C: Supplementary File 3: Analysis Code and Scripts

This supplementary file contains the complete Python analysis code and scripts used for statistical analyses, including non-parametric tests (Kruskal-Wallis, Dunn’s post-hoc, Friedman’s test, Wilcoxon signed-rank), correlation analyses (Spearman), and qualitative thematic analysis procedures. The scripts enable full reproducibility of the study results.
