# Supplementary material for: Understanding Clinician Perceptions of GenAI: A Mixed Methods Analysis of Clinical Documentation Tasks
Source: J Med Syst. 2025 Aug 2;49(1):101. doi: 10.1007/s10916-025-02234-8 (PMC12317912; doi:10.1007/s10916-025-02234-8)
Supplement: Supplementary file 1 — (pdf 3372 KB) [file 10916_2025_2234_MOESM1_ESM.pdf]

Welcome to MagicGP!

I will show you how you can use or Patient record interface to perform easily your day-to-day tasks

Click NEXT when you are ready

Next

Mrs. Jane Doe – 34 years – Pt Number: 360877 – MagicGP version 0.1

Jane Doe | DOB: 01/01/1988 (34 years) | Occupation: Marketing | Address: 109 Kirribilli Av, Kirribilli NSW 2061 Ph: (02) 1234 5678

Allergies: None | Smoking Hx: Never smoked | ATSI: Neither Aboriginal nor Torres Strait Islander

Warnings:

Progress note – 27/01/2022

Mrs. Doe is c/o of a sore throat f[illegible] couple of days. COVID-19 RAT te[illegible] negative. Fever the last 24h, pre[illegible] episodes.

She also has seen her endocrino[illegible] week and came to review results[illegible] management.

Examination:
Temp 37.5ºC HR: 95 BPM BP: 127/72
Neck: Swollen submandibular lymph nodes

This is **MagicGP** main interface… It should resemble a bit your day-to-day *MedicalRecord* or your local Electronic Health Record Software.

We will begin exploring basic elements of the interface.

Next

Dipstick:

Activity Panel

Treatments

Levothyroxine 50

Desogestrel 75

Bilastine 20

Labs, Referrals & Others

Pending Labs: 0

Referrals: 1

Ultrasound Scan: 1

Highlight | Extract | Extract ALL | Add to EHR | Edit | Add ALL | Confirm | Cancel

Mrs. Jane Doe – 34 years – Pt Number: 360877 – MagicGP version 0.1

Jane Doe | DOB: 01/01/1988 (34 years) | Occupation: Marketing | Address: 109 Kirribilli Av, Kirribilli NSW 2061 Ph: (02) 1234 5678

Allergies: None | Smoking Hx: Never smoked | ATSI: Neither Aboriginal nor Torres Strait Islander

Warnings: | Recalls

Progress note – 27/01/2022

Mrs. Doe is c/o of a sore throat for the last couple of days. COVID-19 RAT testing negative. Fever the last 24h, previous similar episodes.

She also has seen her endocrinologist last week and came to review results and discuss management.

Examination:
Temp 37.5ºC HR: 95 BPM BP: 127/72
Neck: Swollen submandibular lymph nodes

Previous visits

Date	Recorded by:	Reason for contact
**01/21/2021**	**Dr. Smith**	**Urinary Tract Infection**
03/04/2021	Dr. Singh	Sprained ankle
14/07/2021	Dr. Cheng	Sleeping problems
05/10/2021	Mrs. Johnson	Pap Smear

C/O pain when passing uri[illegible] frequency for the last 36 h[illegible]

No fever, back pain or oth[illegible]

Dipstick:

Activity Panel

Treatments
Levothyroxine 50
Desogestrel 75
Bilastine 20

This is the Window Title

Next

Highlight | Extract | Extract ALL | Add to EHR | Edit

Mrs. Jane Doe – 34 years – Pt Number: 360877 – MagicGP version 0.1

Jane Doe | DOB: 01/01/1988 (34 years) | Occupation: Marketing | Address: 109 Kirribilli Av, Kirribilli NSW 2061 Ph: (02) 1234 5678

Allergies: None | Smoking Hx: Never smoked | ATSI: Neither Aboriginal nor Torres Strait Islander

Warnings: | Recalls

Progress note – 27/01/2022

Mrs. Doe is c/o of a sore throat for the last couple of days. COVID-19 RAT testing negative. Fever the last 24h, previous similar episodes.

She also has seen her endocrinologist last week and came to review results and discuss management.

Examination:
Temp 37.5ºC HR: 95 BPM BP: 127/72
Neck: Swollen submandibular lymph nodes

Previous visits

Date	Recorded by:	Reason for contact
**01/21/2021**	**Dr. Smith**	**Urinary Tract Infection**
03/04/2021	Dr. Singh	Sprained ankle
14/07/2021	Dr. Cheng	Sleeping prob[illegible]
05/10/2021	Mrs. Johnson	Pap Smear

C/O pain when passing urine, [illegible] frequency for the last 36 hour[illegible]

No fever, back pain or other sy[illegible]

Dipstick:

Activity Panel

Treatments
Levothyroxine 50

These are buttons

Next

Highlight | Extract | Extract ALL | Add to EHR | Edit | Add ALL | Confirm | Cancel

Mrs. Jane Doe – 34 years – Pt Number: 360877 – MagicGP version 0.1

Jane Doe | DOB: 01/01/1988 (34 years) | Occupation: Marketing | Address: 109 Kirribilli Av, Kirribilli NSW 2061 Ph: (02) 1234 5678

Allergies: None | Smoking Hx: Never smoked | ATSI: Neither Aboriginal nor Torres Strait Islander

Warnings: | Recalls

Progress note – 27/01/2022

Mrs. Doe is c/o of a sore throat for the last couple of days. COVID-19 RAT testing negative. Fever the last 24h, previous similar episodes.

She also has seen her endocrinologist last week and came to review results and discuss management.

Examination:
Temp 37.5ºC HR: 95 BPM BP: 127/72
Neck: Swollen submandibular lymph nodes

Previous visits

Date	Recorded by:	Reason for contact
**01/21/2021**	**Dr. Smith**	**Urinary Tract Infection**
03/04/2021	Dr. Singh	Sprained ankle
14/07/2021	Dr. Cheng	Sleeping problems
05/10/2021	Mrs. Johnson	Pap Smear

C/O pain when passing urine frequency for the last 36 hou

No fever, back pain or other

Dipstick:

Activity Panel

Treatments
Levothyroxine 50
Desogestrel 75
Bilastine 20

This is the patient administrative data area

Next

Highlight | Extract | Extract ALL | Add to EHR | Edit

Mrs. Jane Doe – 34 years – Pt Number: 360877 – MagicGP version 0.1

Jane Doe	DOB: 01/01/1988 (34 years)	Occupation: Marketing	Address: 109 Kirribilli Av, Kirribilli NSW 2061 Ph: (02) 1234 5678
Allergies: None			Smoking Hx: Never smoked ATSI: Neither Aboriginal nor Torres Strait Islander
Warnings:			Recalls

Progress note – 27/01/2022

Mrs. Doe is c/o of a sore throat for the last couple of days. COVID-19 RAT testing negative. Fever the last 24h, previous similar episodes.

She also has seen her endocrinologist last week and came to review results and discuss management.

Examination:
Temp 37.5ºC HR: 95 BPM BP: 127/72
Neck: Swollen submandibular lymph nodes

Previous visits

Date	Recorded by:	Reason for contact
**01/21/2021**	**Dr. Smith**	**Urinary Tract Infection**
03/04/2021	Dr. Singh	Sprained ankle
14/07/2021	Dr. Cheng	Sleeping problems
05/10/2021	Mrs. Johnson	Pap Smear

C/O pain when passing urine, increased frequency for the last 36 hours.

No fever, back pain or other symptoms

Dipstick:

Activity Panel

Treatments
Levothyroxine 50
Desogestrel 75
Bilastine 20

Labs, Referrals & Others
Pending Labs: 0
Referrals: 1
Ultrasound Scan: 1

Highlight | Extract | Extract ALL | Add to EHR | Edit | Add ALL | Confirm | Cancel

Mrs. Jane Doe – 34 years – Pt Number: 360877 – MagicGP version 0.1

Jane Doe DOB: 01/01/1988 (34 years) Occupation: Marketing Address: 109 Kirribilli Av, Kirribilli NSW 2061 Ph: (02) 1234 5678

Allergies: None Smoking Hx: Never smoked ATSI: Neither Aboriginal nor Torres Strait Islander

Warnings: Recalls

Progress note – 27/01/2022

Mrs. Doe is c/o of a sore throat for the last couple of days. COVID-19 RAT testing negative. Fever the last 24h, previous similar episodes.

She also has seen her endocrinologist last week and came to review results and discuss management.

Examination:
Temp 37.5ºC HR: 95 BPM BP: 127/72
Neck: Swollen submandibular lymph nodes

Highlight | Extract | Extract ALL

Previous visits

Date	Recorded by:	Reason for contact
**0[illegible]/21/2021**	**Dr. Smith**	**Urinary Tract Infection**
03/04/2021	Dr. Singh	Sprained ankle
14/07/2021	Dr. Cheng	Sleeping problems
05/10/2021	Mrs. Johnson	Pap Smear

[illegible]/O pain when passing urine, increased [illegible]requency for the last 36 hours.

[illegible]o fever, back pain or other sy[illegible]

[illegible]ipstick:

Add to EHR | Edit

Activity Panel

Treatments
Levothyroxine 50
Desogestrel 75
Bilastine 20

Labs, Referrals & Others
Pending Labs: 0

This is the note writing and letter reading area

Next

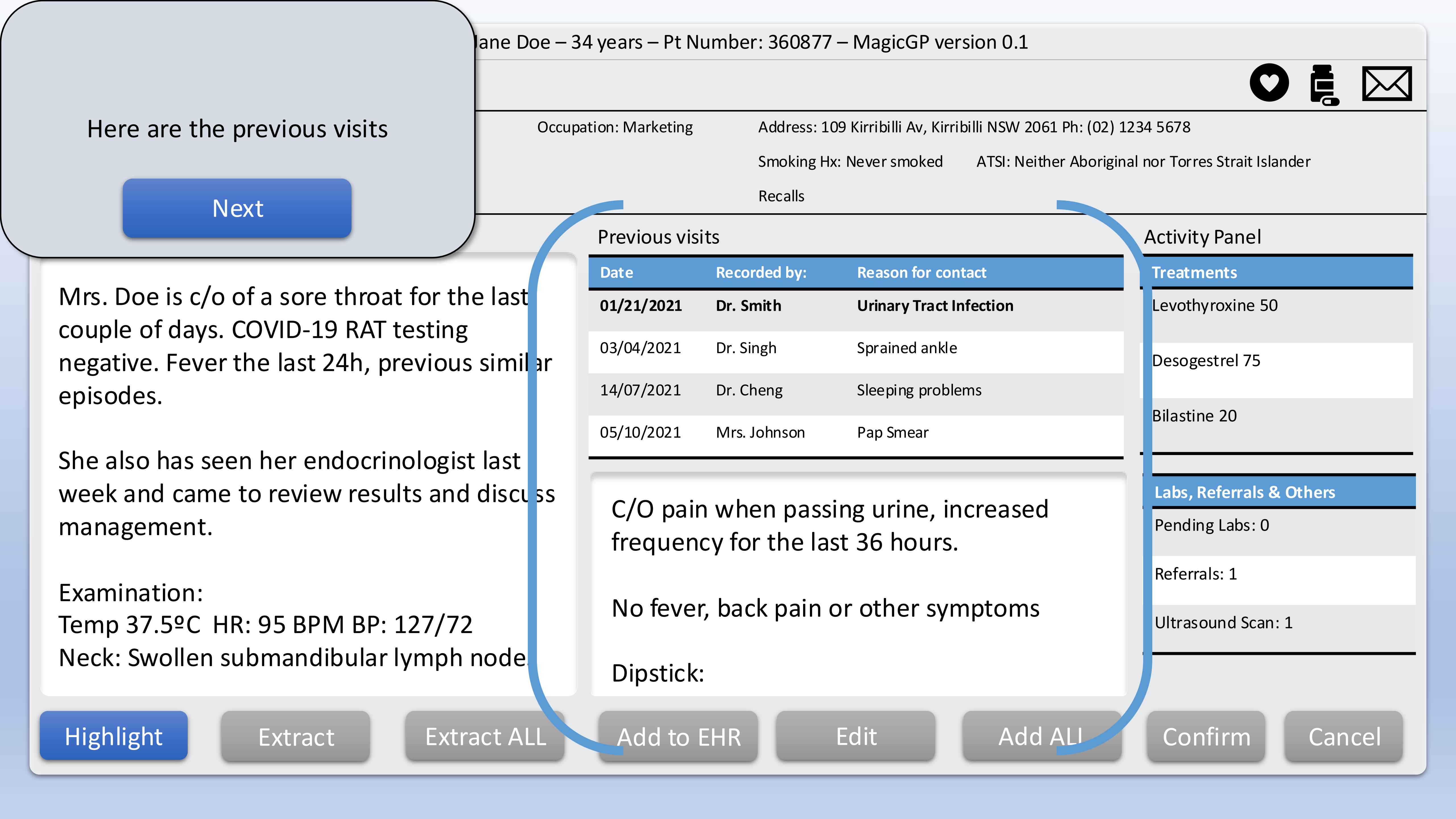
Here are the previous visits
Next
Jane Doe – 34 years – Pt Number: 360877 – MagicGP version 0.1
Occupation: Marketing
Address: 109 Kirribilli Av, Kirribilli NSW 2061 Ph: (02) 1234 5678
Smoking Hx: Never smoked
ATSI: Neither Aboriginal nor Torres Strait Islander
Recalls
Mrs. Doe is c/o of a sore throat for the last couple of days. COVID-19 RAT testing negative. Fever the last 24h, previous similar episodes.
She also has seen her endocrinologist last week and came to review results and discuss management.
Examination:
Temp 37.5ºC HR: 95 BPM BP: 127/72
Neck: Swollen submandibular lymph node
Previous visits
Date
Recorded by:
Reason for contact
01/21/2021
Dr. Smith
Urinary Tract Infection
03/04/2021
Dr. Singh
Sprained ankle
14/07/2021
Dr. Cheng
Sleeping problems
05/10/2021
Mrs. Johnson
Pap Smear
C/O pain when passing urine, increased frequency for the last 36 hours.
No fever, back pain or other symptoms
Dipstick:
Activity Panel
Treatments
Levothyroxine 50
Desogestrel 75
Bilastine 20
Labs, Referrals & Others
Pending Labs: 0
Referrals: 1
Ultrasound Scan: 1
Highlight
Extract
Extract ALL
Add to EHR
Edit
Add ALL
Confirm
Cancel

Mrs. Jane Doe – 34 years – Pt Number: 360877 – MagicGP version 0.1

Jane Doe DOB: 01/01/1988 (34 years) Occupation: Marketing Address: 109 Kirribilli Av, Kirribilli NSW 2061 Ph: (02) 1234 5678

Allergies: None Smoking Hx: Never smoked ATSI: Neither Aboriginal nor Torres Strait Islander

Warnings: Recalls

Progress note – 27/01/2022

Mrs. Doe is c/o of a sore throat for the last couple of days. COVID-19 RAT testing negative. Fever the last 24h, previous similar episodes.

She also has seen her endocrinologist last week and came to review results and discuss management.

[illegible]nodes

Previous visits

Date	Recorded by:	Reason for contact
**01/21/2021**	**Dr. Smith**	**Urinary Tract Infection**
03/04/2021	Dr. Singh	Sprained ankle
14/07/2021	Dr. Cheng	Sleeping problems
05/10/2021	Mrs. Johnson	Pap Smear

C/O pain when passing urine, increased frequency for the last 36 hours.

No fever, back pain or other symptoms

Dipstick:

Activity Panel

Treatments
Levothyroxine 50
Desogestrel 75
Bilastine 20

Labs, Referrals & Others
Pending Labs: 0
Referrals: 1
Ultrasound Scan: 1

[illegible]ct ALL | Add to EHR | Edit | Add ALL | Confirm | Cancel

This area shows the active treatments and requests

Next

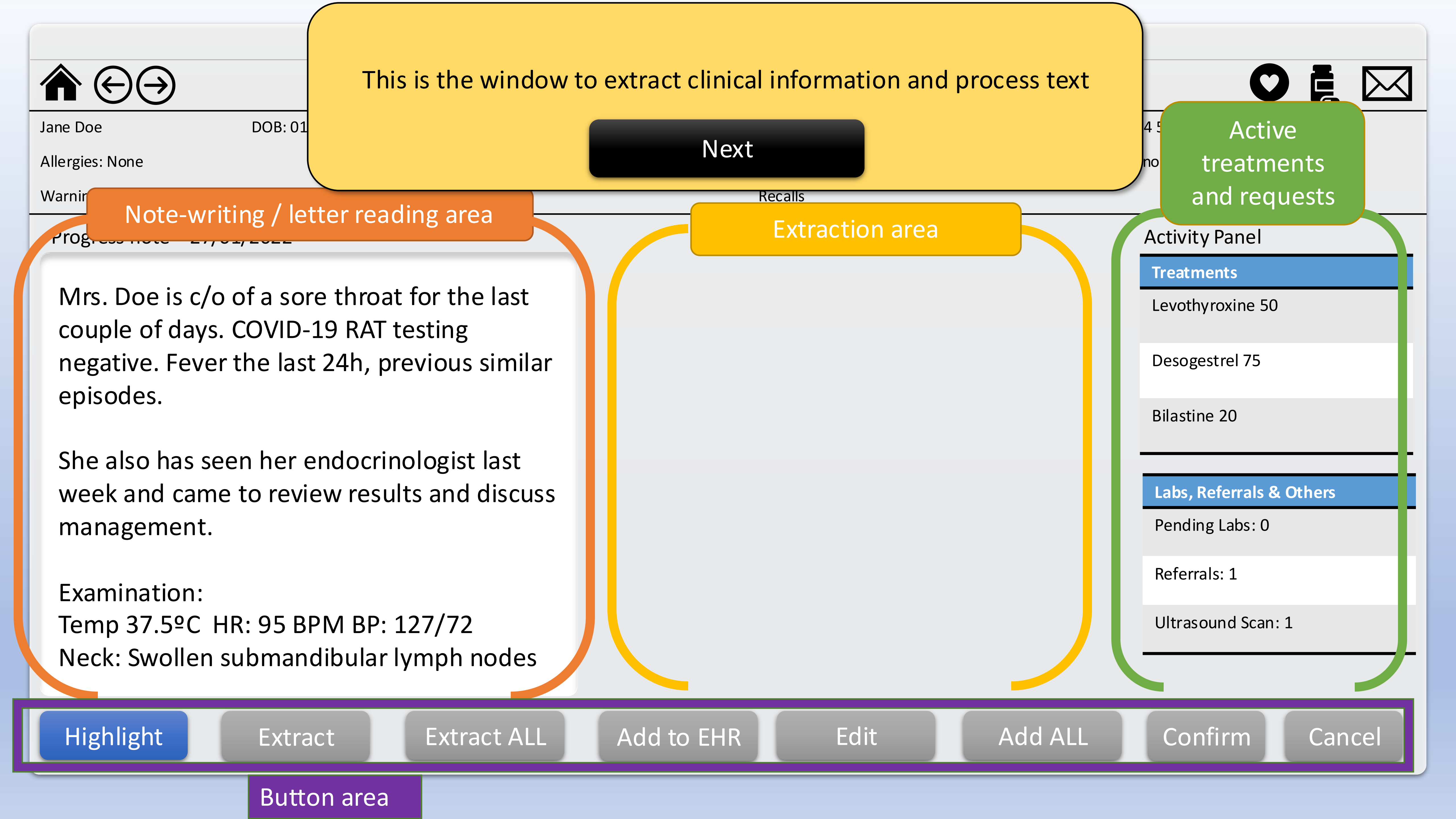
This is the window to extract clinical information and process text
Next
Jane Doe
DOB: 01
Allergies: None
Warnin
Recalls
Active treatments and requests
Note-writing / letter reading area
Extraction area
Mrs. Doe is c/o of a sore throat for the last couple of days. COVID-19 RAT testing negative. Fever the last 24h, previous similar episodes.
She also has seen her endocrinologist last week and came to review results and discuss management.
Examination:
Temp 37.5ºC HR: 95 BPM BP: 127/72
Neck: Swollen submandibular lymph nodes
Activity Panel
Treatments
Levothyroxine 50
Desogestrel 75
Bilastine 20
Labs, Referrals & Others
Pending Labs: 0
Referrals: 1
Ultrasound Scan: 1
Highlight
Extract
Extract ALL
Add to EHR
Edit
Add ALL
Confirm
Cancel
Button area

Mrs. Jane Doe – 34 years – Pt Number: 360877 – MagicGP version 0.1

Jane Doe DOB: 01/01/1988 (34 yea[illegible] [illegible]illi NSW 2061 Ph: (02) 1234 5678

Allergies: None ATSI: Neither Aboriginal nor Torres Strait Islander

Warnings:

Progress note – 27/01/2022

Mrs. Doe is c/o of a sore throat f[illegible] couple of days. COVID-19 RAT te[illegible] negative. Fever the last 24h, pre[illegible] episodes.

She also has seen her endocrino[illegible] week and came to review results[illegible] management.

Examination:
Temp 37.5ºC HR: 95 BPM BP: 12[illegible]
Neck: Swollen submandibular ly[illegible]

Alright, that was intense!

Whenever you are ready click
next so we will begin
With our first scenario

Next

Activity Panel

Treatments

Levothyroxine 50

Desogestrel 75

Bilastine 20

Labs, Referrals & Others

Pending Labs: 0

Referrals: 1

Ultrasound Scan: 1

Highlight | Extract | Extract ALL | Add to EHR | Edit | Add ALL | Confirm | Cancel

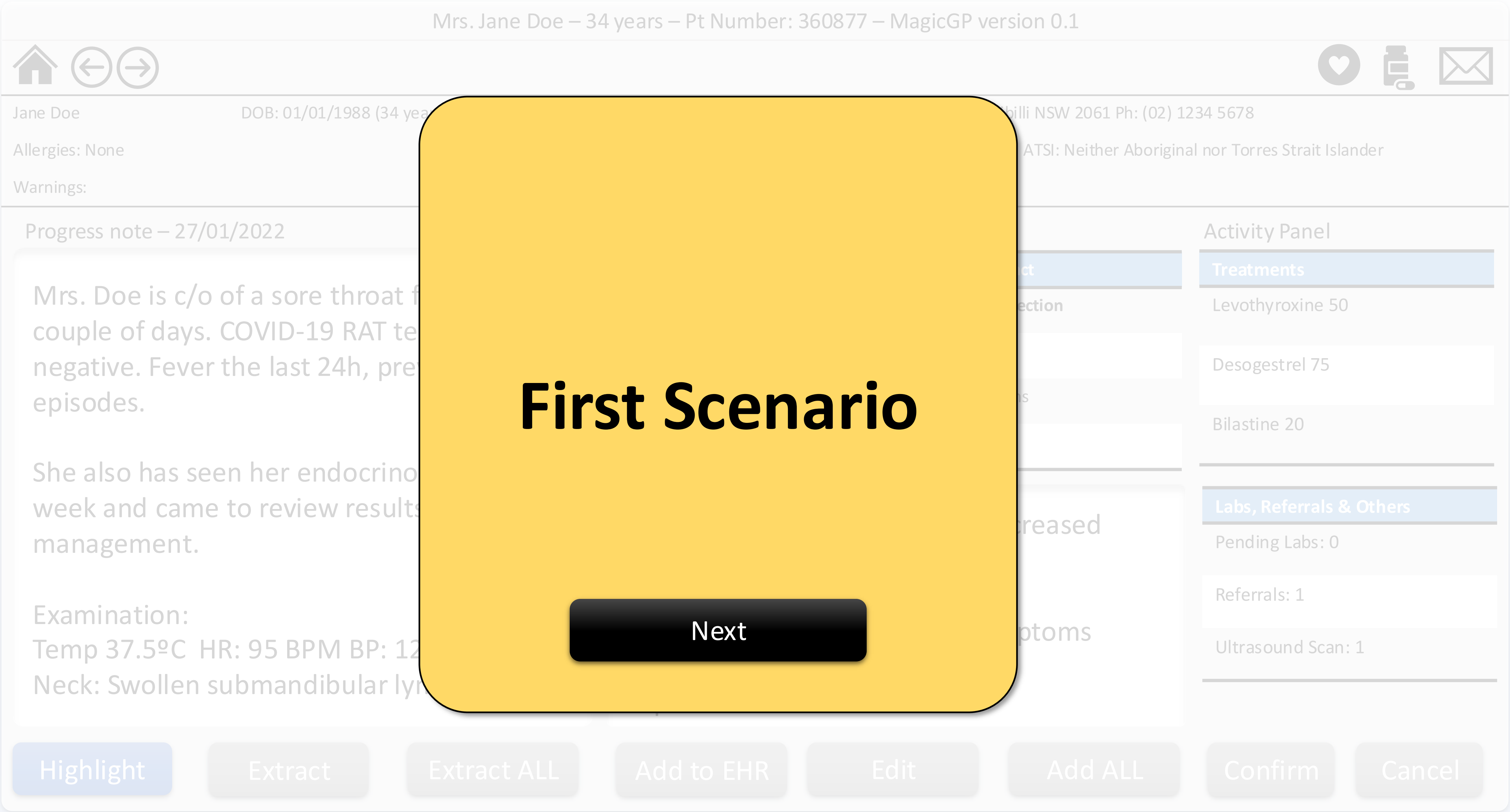
Mrs. Jane Doe – 34 years – Pt Number: 360877 – MagicGP version 0.1
Jane Doe
DOB: 01/01/1988 (34 yea
billi NSW 2061 Ph: (02) 1234 5678
Allergies: None
ATSI: Neither Aboriginal nor Torres Strait Islander
Warnings:
Progress note – 27/01/2022
Mrs. Doe is c/o of a sore throat f
couple of days. COVID-19 RAT te
negative. Fever the last 24h, pre
episodes.
She also has seen her endocrino
week and came to review results
management.
Examination:
Temp 37.5ºC HR: 95 BPM BP: 12
Neck: Swollen submandibular lyr
First Scenario
Next
Activity Panel
Treatments
Levothyroxine 50
Desogestrel 75
Bilastine 20
Labs, Referrals & Others
Pending Labs: 0
Referrals: 1
Ultrasound Scan: 1
Highlight
Extract
Extract ALL
Add to EHR
Edit
Add ALL
Confirm
Cancel

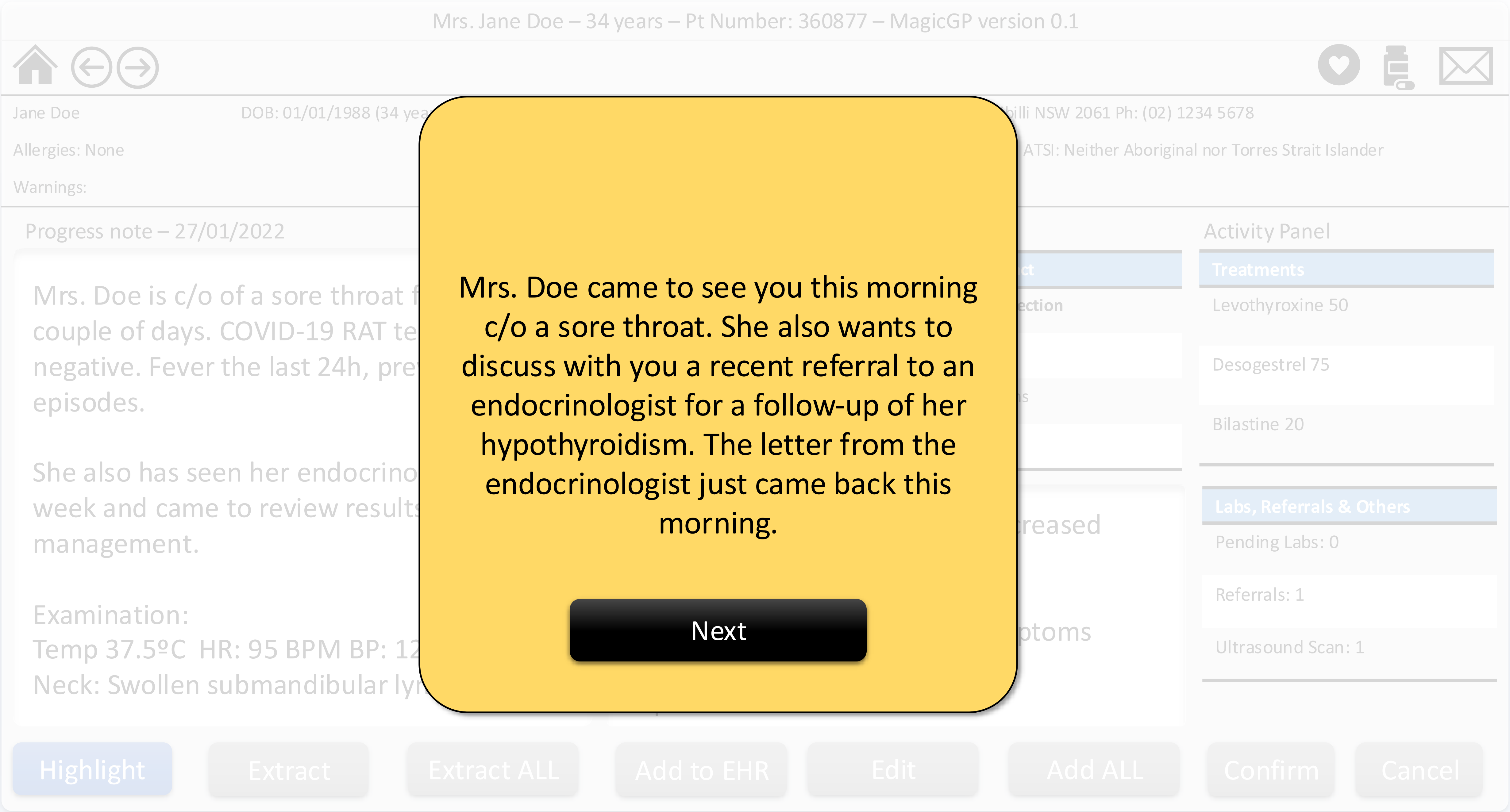
Mrs. Jane Doe – 34 years – Pt Number: 360877 – MagicGP version 0.1
Jane Doe
DOB: 01/01/1988 (34 yea
billi NSW 2061 Ph: (02) 1234 5678
Allergies: None
ATSI: Neither Aboriginal nor Torres Strait Islander
Warnings:
Progress note – 27/01/2022
Mrs. Doe is c/o of a sore throat f couple of days. COVID-19 RAT te negative. Fever the last 24h, pre episodes.
She also has seen her endocrino week and came to review results management.
Examination:
Temp 37.5ºC HR: 95 BPM BP: 12
Neck: Swollen submandibular ly
Mrs. Doe came to see you this morning c/o a sore throat. She also wants to discuss with you a recent referral to an endocrinologist for a follow-up of her hypothyroidism. The letter from the endocrinologist just came back this morning.
Next
Activity Panel
Treatments
Levothyroxine 50
Desogestrel 75
Bilastine 20
Labs, Referrals & Others
Pending Labs: 0
Referrals: 1
Ultrasound Scan: 1
Highlight
Extract
Extract ALL
Add to EHR
Edit
Add ALL
Confirm
Cancel

Mrs. Jane Doe – 34 years – Pt Number: 360877 – MagicGP version 0.1

Jane Doe | DOB: 01/01/1988 (34 years) | Occupation: Marketing | Address: 109 Kirribilli Av, Kirribilli NSW 2061 Ph: (02) 1234 5678

Allergies: None | Smoking Hx: Never smoked | ATSI: Neither Aboriginal nor Torres Strait Islander

Warnings: | Recalls

Progress note – 27/01/2022

Mrs. Doe is c/o of a sore throat for the last couple of days. COVID-19 RAT testing negative. Fever the last 24h, previous similar episodes.

She also has seen her endocrinologist last week and came to review results and discuss management.

Examination:
Temp 37.5ºC HR: 95 BPM BP: 127/72
Neck: Swollen submandibular lymph nodes

Previous visits

Date	Recorded by:	Reason for contact
01/21/2[illegible]		
03/04/2[illegible]		
14/07/2[illegible]		
05/10/2[illegible]		

C/O[illegible]
freq[illegible]

No fever, back pain or other symptoms

Dipstick: +++ Nitrites , Blood ++, Rest Nil

It seems they haven't uploaded the letter yet… but let's see what MAGIC GP do.

Next

Activity Panel

Treatments

Levothyroxine 50

Desogestrel 75

Bilastine 20

Labs, Referrals & Others

Pending Labs: 0

Referrals: 1

Ultrasound Scan: 1

Highlight | Extract | Extract ALL | Add to EHR | Edit | Add ALL | Confirm | Cancel

Mrs. Jane Doe – 34 years – Pt Number: 360877 – MagicGP version 0.1

Jane Doe | DOB: 01/01/1988 (34 years) | Occupation: Marketing | Address: 109 Kirribilli Av, Kirribilli NSW 2061 Ph: (02) 1234 5678

Allergies: None | Smoking Hx: Never smoked | ATSI: Neither Aboriginal nor Torres Strait Islander

Warnings: | Recalls

Progress note – 27/01/2022

Mrs. Doe is c/o of a sore throat for the last couple of days. COVID-19 RAT testing negative. Fever the last 24h, previous similar episodes.

She also has seen her endocrinologist last week and came to review results and discuss management.

Examination:
Temp 37.5ºC HR: 95 BPM BP: 127/72
Neck: Swollen submandibular lymph nodes

Date	Recorded by:	Reason for contact
**01/21/2021**	**Dr. Smith**	**Urinary Tract Infection**
03/04/2021	Dr. Singh	Sprained ankle
14/07/2021	Dr. Cheng	Sleeping problems
05/10/2[illegible]	[illegible]	[illegible]

Click Highlight

C/O [illegible] frequency for the last 36 hours.

No fever, back pain or other symptoms

Dipstick: +++ Nitrites , Blood ++, Rest Nil

Activity Panel

Treatments
Levothyroxine 50
Desogestrel 75
Bilastine 20

Labs, Referrals & Others
Pending Labs: 0
Referrals: 1
Ultrasound Scan: 1

Highlight | Extract | Extract ALL | Add to EHR | Edit | Add ALL | Confirm | Cancel

Mrs. Jane Doe – 34 years – Pt Number: 360877 – MagicGP version 0.1

Jane Doe | DOB: 01/01/1988 (34 years) | Occupation: Marketing | Address: 109 Kirribilli Av, Kirribilli NSW 2061 Ph: (02) 1234 5678

Allergies: None | Smoking Hx: Never smoked | ATSI: Neither Aboriginal nor Torres Strait Islander

Warnings: | Recalls

Progress note – 27/01/2022

Mrs. Doe is c/o of a sore throat for the last couple of days. COVID-19 RAT testing negative. Fever the last 24h, previous similar episodes.

She also has seen her endocrinologist last week and came to review results and discuss management.

Examination:
Temp 37.5ºC HR: 95 BPM BP: 127/72
Neck: Swollen submandibular lymph nodes

Date	Recorded by:	Reason for contact
01/21/2[illegible]		
03/04/2[illegible]		
14/07/2[illegible]		
05/10/2[illegible]		

Now let's select one of the text findings.

Click "COVID-19 RAT"

C/O pain when passing urine, increased frequency for the last 36 hours.

No fever, back pain or other symptoms

Dipstick: +++ Nitrites , Blood ++, Rest Nil

Activity Panel

Treatments
Levothyroxine 50
Desogestrel 75
Bilastine 20

Labs, Referrals & Others
Pending Labs: 0
Referrals: 1
Ultrasound Scan: 1

Highlight | Extract | Extract ALL | Add to EHR | Edit | Add ALL | Confirm | Cancel

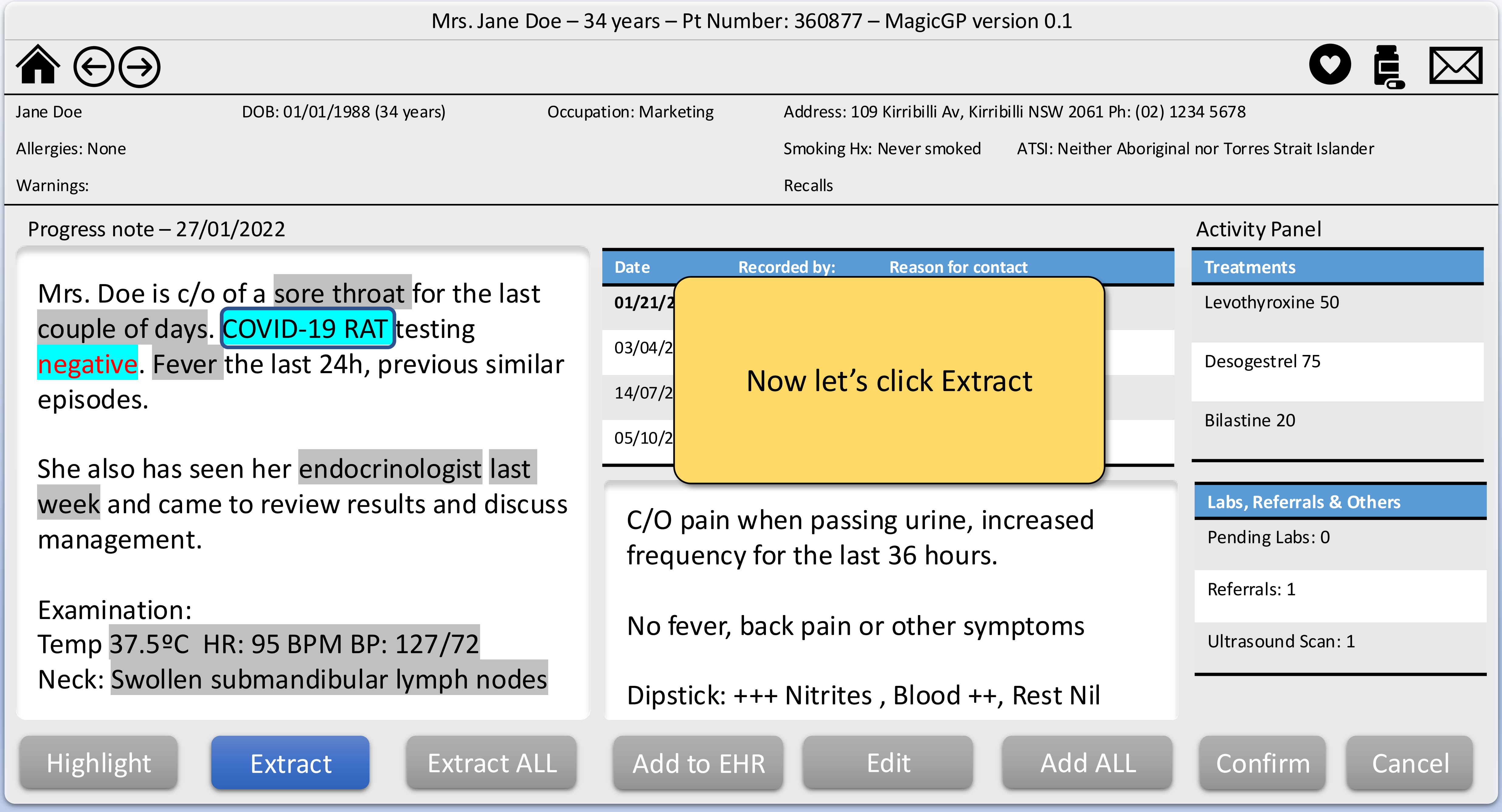

Mrs. Jane Doe – 34 years – Pt Number: 360877 – MagicGP version 0.1
Jane Doe
DOB: 01/01/1988 (34 years)
Occupation: Marketing
Address: 109 Kirribilli Av, Kirribilli NSW 2061 Ph: (02) 1234 5678
Allergies: None
Smoking Hx: Never smoked
ATSI: Neither Aboriginal nor Torres Strait Islander
Warnings:
Recalls
Progress note – 27/01/2022
Mrs. Doe is c/o of a sore throat for the last couple of days. COVID-19 RAT testing negative. Fever the last 24h, previous similar episodes.
She also has seen her endocrinologist last week and came to review results and discuss management.
Examination:
Temp 37.5ºC HR: 95 BPM BP: 127/72
Neck: Swollen submandibular lymph nodes
Date
Recorded by:
Reason for contact
01/21/2
03/04/2
14/07/2
05/10/2
Now let’s click Extract
C/O pain when passing urine, increased frequency for the last 36 hours.
No fever, back pain or other symptoms
Dipstick: +++ Nitrites , Blood ++, Rest Nil
Activity Panel
Treatments
Levothyroxine 50
Desogestrel 75
Bilastine 20
Labs, Referrals & Others
Pending Labs: 0
Referrals: 1
Ultrasound Scan: 1
Highlight
Extract
Extract ALL
Add to EHR
Edit
Add ALL
Confirm
Cancel

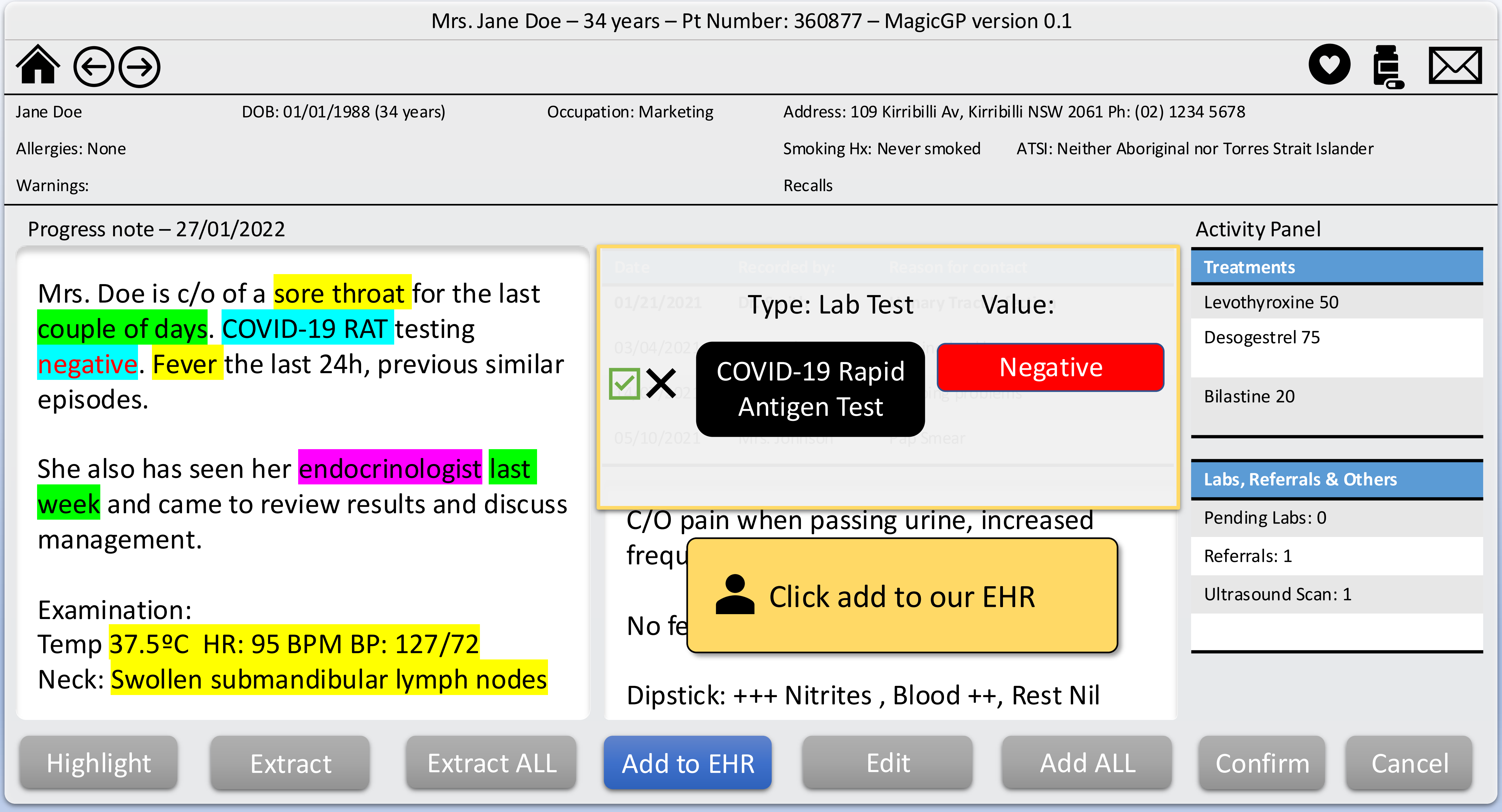

Mrs. Jane Doe – 34 years – Pt Number: 360877 – MagicGP version 0.1
Jane Doe
DOB: 01/01/1988 (34 years)
Occupation: Marketing
Address: 109 Kirribilli Av, Kirribilli NSW 2061 Ph: (02) 1234 5678
Allergies: None
Smoking Hx: Never smoked
ATSI: Neither Aboriginal nor Torres Strait Islander
Warnings:
Recalls
Progress note – 27/01/2022
Mrs. Doe is c/o of a sore throat for the last couple of days. COVID-19 RAT testing negative. Fever the last 24h, previous similar episodes.
She also has seen her endocrinologist last week and came to review results and discuss management.
Examination:
Temp 37.5ºC HR: 95 BPM BP: 127/72
Neck: Swollen submandibular lymph nodes
Type: Lab Test
Value:
COVID-19 Rapid Antigen Test
Negative
C/O pain when passing urine, increased frequ
No fe
Click add to our EHR
Dipstick: +++ Nitrites , Blood ++, Rest Nil
Activity Panel
Treatments
Levothyroxine 50
Desogestrel 75
Bilastine 20
Labs, Referrals & Others
Pending Labs: 0
Referrals: 1
Ultrasound Scan: 1
Highlight
Extract
Extract ALL
Add to EHR
Edit
Add ALL
Confirm
Cancel

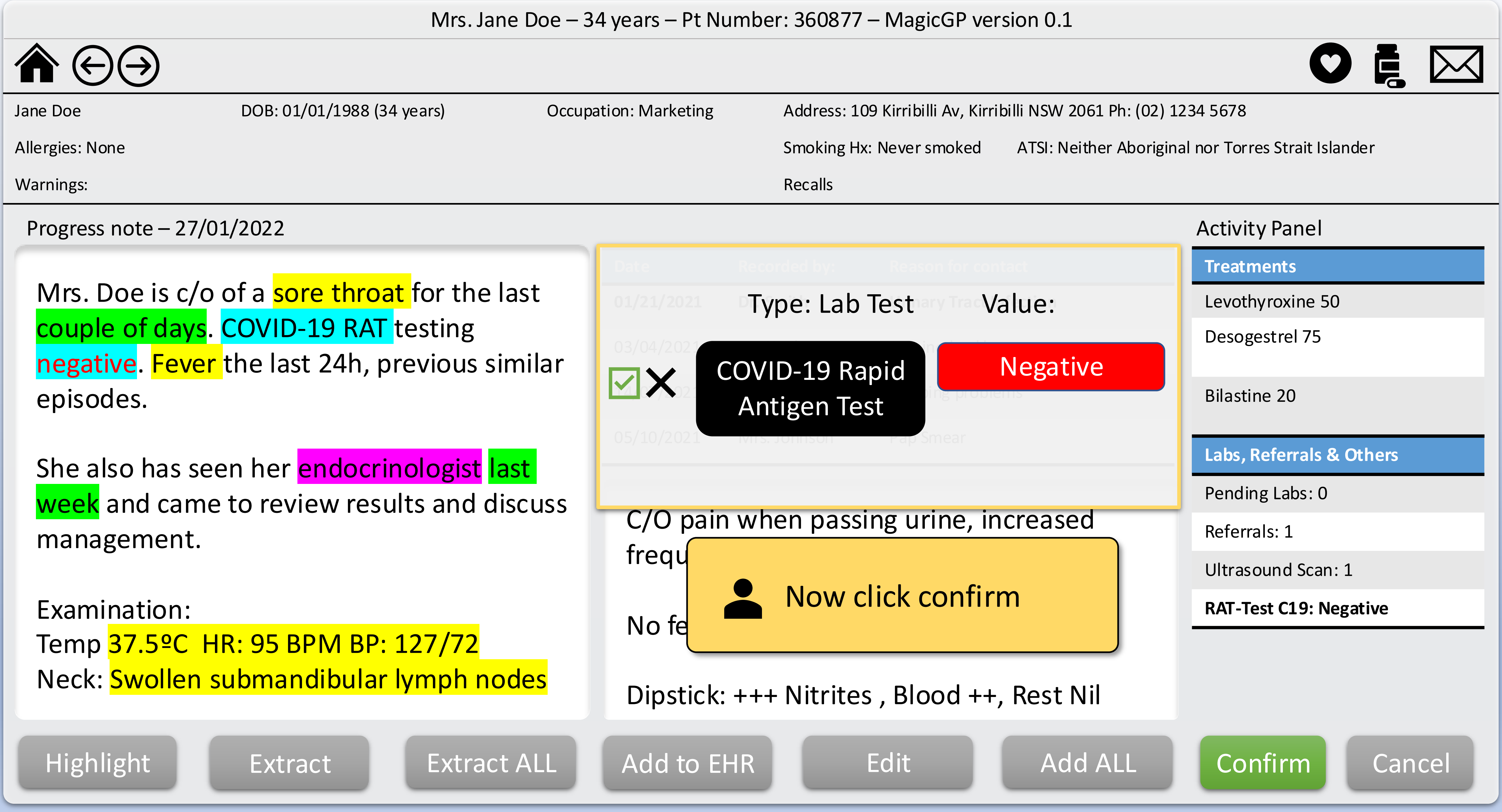
Mrs. Jane Doe – 34 years – Pt Number: 360877 – MagicGP version 0.1
Jane Doe
DOB: 01/01/1988 (34 years)
Occupation: Marketing
Address: 109 Kirribilli Av, Kirribilli NSW 2061 Ph: (02) 1234 5678
Allergies: None
Smoking Hx: Never smoked
ATSI: Neither Aboriginal nor Torres Strait Islander
Warnings:
Recalls
Progress note – 27/01/2022
Mrs. Doe is c/o of a sore throat for the last couple of days. COVID-19 RAT testing negative. Fever the last 24h, previous similar episodes.
She also has seen her endocrinologist last week and came to review results and discuss management.
Examination:
Temp 37.5ºC HR: 95 BPM BP: 127/72
Neck: Swollen submandibular lymph nodes
Type: Lab Test
Value:
COVID-19 Rapid Antigen Test
Negative
C/O pain when passing urine, increased
Now click confirm
Dipstick: +++ Nitrites , Blood ++, Rest Nil
Activity Panel
Treatments
Levothyroxine 50
Desogestrel 75
Bilastine 20
Labs, Referrals & Others
Pending Labs: 0
Referrals: 1
Ultrasound Scan: 1
RAT-Test C19: Negative
Highlight
Extract
Extract ALL
Add to EHR
Edit
Add ALL
Confirm
Cancel

Mrs. Jane Doe – 34 years – Pt Number: 360877 – MagicGP version 0.1

You got a new letter!

1

Jane Doe	DOB: 01/01/1988 (34 years)	Occupation: Marketing	Address: 109 Kirribilli Av, Kirribilli NSW 2061 Ph: (02) 1234 5678
Allergies: None			Smoking Hx: Never smoked ATSI: Neither Aboriginal nor Torres Strait Islander
Warnings:			Recalls

Progress note – 27/01/2022

Mrs. Doe is c/o of a sore throat for the last couple of days. COVID-19 RAT testing negative. Fever the last 24h, previous similar episodes.

She also has seen her endocrinologist last week and came to review results and discuss management.

Examination:
Temp 37.5ºC HR: 95 BPM BP: 127/72
Neck: Swollen submandibular lymph nodes

Previous visits

Date	Recorded by:	Reason for contact
**01/21/2021**	**Dr. Smith**	**Urinary Tract Infection**
03/04/2021	Dr. Singh	Sprained ankle
14/07/2021	Dr. Cheng	Sleeping problems
05/10/2021	Mrs. Johnson	Pap Smear

C/O pain when passing urine, increased frequency for the last 36 hours.

No fever, back pain or other symptoms

Dipstick:

Activity Panel

Treatments
Levothyroxine 50
Desogestrel 75
Bilastine 20

Labs, Referrals & Others
Pending Labs: 0
Referrals: 1
Ultrasound Scan: 1
**RAT-Test C19: Negative**

Highlight | Extract | Extract ALL | Add to EHR | Edit | Add ALL | Confirm | Cancel

Mrs. Jane Doe – 34 years – Pt Number: 360877 – MagicGP version 0.1

Jane Doe | DOB: 01/01/1988 (34 years) | Occupation: Marketing | Address: 109 Kirribilli Av, Kirribilli NSW 2061 Ph: (02) 1234 5678

Allergies: None | Smoking Hx: Never smoked | ATSI: Neither Aboriginal nor Torres Strait Islander

Warnings: | Recalls

New Email – Received 27/01/2022

Dr. Riviera

Endocrinology Clinic
Sydney CBD

I reviewed Mrs. Doe primary hypothyroidism today. She felt she's been more tired the last four weeks and thinks she has gained some weight

Weight: 85 Kg (previous 80)

Tests: TSH: 14 mIU/L , Total T4: 0.2 µg/dL

Diagnosis: Undertreated primary hypothyroidism.

Plan: Increasing dose of Eutroxsig to 75 mcg OD, review in 6 weeks.

Mail Inbox

Date	From	Title
**27/01/2022**	**Endocrinology Cl**	**Mrs. Doe Review – Dr Riviera**
03/12/2021	Sydney Labs	Lab Results
14/07/2021	ED St. Vincent	Acute Care - Back pain
05/10/2021	Sydney Labs	Pap Smear

OK let's try this again here
Highlight

Activity Panel

Treatments
Levothyroxine 50
Desogestrel 75
Bilastine 20

Labs, Referrals & Others
Pending Labs: 0
Referrals: 1
Ultrasound Scan: 1

Highlight | Extract | Extract ALL | Add to EHR | Edit | Add ALL | Confirm | Cancel

Mrs. Jane Doe – 34 years – Pt Number: 360877 – MagicGP version 0.1

Jane Doe (she/her)	DOB: 01/01/1988 (34 years)	Occupation: Marketing	Address: 109 Kirribilli Av, Kirribilli NSW 2061 Ph: (02) 1234 5678
Allergies: None			Smoking Hx: Never smoked ATSI: Neither Aboriginal nor Torres Strait Islander
Warnings:			Recalls

New Email – Received 27/01/2022

Dr. Riviera

Endocrinology Clinic
Sydney CBD

I reviewed Mrs. Doe primary hypothyroidism today. She felt she's been more tired the last four weeks and thinks she has gained some weight

Weight: 85 Kg (previous 80)

Tests: TSH: 14 mIU/L , Total T4: 0.2 µg/dL

Diagnosis: Undertreated primary hypothyroidism.

Plan: Increasing dose of Eutroxsig to 75 mcg OD, review in 6 weeks.

Mail Inbox

Date	From	Title
**27/01/2022**	**Endocrinology Cl**	**Mrs. Doe Review – Dr Riviera**
03/12/2021	Sydney Labs	Lab Results
14/07/2021	ED St. Vincent	Acute Care - Back pain
05/10/2021	Sydney Labs	Pap Smear

Now click on TSH and T4

Activity Panel

Treatments
Levothyroxine 50
Desogestrel 75
Bilastine 20

Labs, Referrals & Others
Pending Labs: 0
Referrals: 1
Ultrasound Scan: 1

Highlight | Extract | Extract ALL | Add to EHR | Edit | Add ALL | Confirm | Cancel

Mrs. Jane Doe – 34 years – Pt Number: 360877 – MagicGP version 0.1

Jane Doe | DOB: 01/01/1988 (34 years) | Occupation: Marketing | Address: 109 Kirribilli Av, Kirribilli NSW 2061 Ph: (02) 1234 5678

Allergies: None | Smoking Hx: Never smoked | ATSI: Neither Aboriginal nor Torres Strait Islander

Warnings: | Recalls

New Email – Received 27/01/2022

Dr. Riviera — Endocrinology Clinic Sydney CBD

I reviewed Mrs. Doe primary hypothyroidism today. She felt she's been more tired the last four weeks and thinks she has gained some weight

Weight: 85 Kg (previous 80)

Tests: TSH: 14 mIU/L, Total T4: 0.2 µg/dL

Diagnosis: Undertreated primary hypothyroidism.

Plan: Increasing dose of Eutroxsig to 75 mcg OD, review in 6 weeks.

Mail Inbox

Date	From	Title
**27/01/2022**	**Endocrinology Cl**	**Mrs. Doe Review – Dr Riviera**
03/12/2021	Sydney Labs	Lab Results
14/07/2021	ED St. Vincent	Acute Care - Back pain
05/10/2021	Sydney Labs	Pap Smear

and T4

Activity Panel

Treatments
Levothyroxine 50
Desogestrel 75
Bilastine 20

Labs, Referrals & Others
Pending Labs: 0
Referrals: 1
Ultrasound Scan: 1

Highlight | Extract | Extract ALL | Add to EHR | Edit | Add ALL | Confirm | Cancel

Mrs. Jane Doe – 34 years – Pt Number: 360877 – MagicGP version 0.1

Jane Doe DOB: 01/01/1988 (34 years) Occupation: Marketing Address: 109 Kirribilli Av, Kirribilli NSW 2061 Ph: (02) 1234 5678

Allergies: None Smoking Hx: Never smoked ATSI: Neither Aboriginal nor Torres Strait Islander

Warnings: Recalls

New Email – Received 27/01/2022

Dr. Riviera

Endocrinology Clinic
Sydney CBD

I reviewed Mrs. Doe primary hypothyroidism today. She felt she's been more tired the last four weeks and thinks she has gained some weight

Weight: 85 Kg (previous 80)

Tests: TSH: 14 mIU/L, Total T4: 0.2 µg/dL

Diagnosis: Undertreated primary hypothyroidism.

Plan: Increasing dose of Eutroxsig to 75 mcg OD, review in 6 weeks.

Mail Inbox

Date	From	Title
**27/01/2022**	**Endocrinology Cl**	**Mrs. Doe Review – Dr Riviera**
03/12/2021	Sydney Labs	Lab Results
14/07/2021	ED St. Vincent	Acute Care - Back pain
05/10/2021	Sydney Labs	Pap Smear

Click Extract

Activity Panel

Treatments
Levothyroxine 50
Desogestrel 75
Bilastine 20

Labs, Referrals & Others
Pending Labs: 0
Referrals: 1
Ultrasound Scan: 1

Highlight | Extract | Extract ALL | Add to EHR | Edit | Add ALL | Confirm | Cancel

Mrs. Jane Doe – 34 years – Pt Number: 360877 – MagicGP version 0.1

Jane Doe	DOB: 01/01/1988 (34 years)	Occupation: Marketing	Address: 109 Kirribilli Av, Kirribilli NSW 2061 Ph: (02) 1234 5678
Allergies: None			Smoking Hx: Never smoked ATSI: Neither Aboriginal nor Torres Strait Islander
Warnings:			Recalls

Progress note – 27/01/2022

Dr. Riviera

Endocrinologist clinic
Sydney CBD

I reviewed Mrs. Doe primary hypothyroidism today. She felt she's been more tired the last four weeks and thinks she has gained some weight
Weight: 85 Kg (previous 80)
Tests: TSH: 14 mIU/L , Total T4: 0.2 µg/dL
Diagnosis: Undertreated primary hypothyroidism.
Plan: Increasing dose of Eutroxsig to 75 mcg OD, review in 6 weeks.

Type: Test	Value:
TSH	14 IU/L ↑
T4	0.2 µg/dL ↓

Now add to EHR

Activity Panel

Treatments
- Levothyroxine 50
- Desogestrel 75
- Bilastine 20

Labs, Referrals & Others
- Pending Labs: 0
- Referrals: 1
- Ultrasound Scan: 1

Highlight | Extract | Extract ALL | Add to EHR | Edit | Add ALL | Confirm | Cancel

Mrs. Jane Doe – 34 years – Pt Number: 360877 – MagicGP version 0.1

Jane Doe | DOB: 01/01/1988 (34 years) | Occupation: Marketing | Address: 109 Kirribilli Av, Kirribilli NSW 2061 Ph: (02) 1234 5678

Allergies: None | Smoking Hx: Never smoked | ATSI: Neither Aboriginal nor Torres Strait Islander

Warnings: | Recalls

Progress note – 27/01/2022

Dr. Riviera

Endocrinologist clinic
Sydney CBD

I reviewed Mrs. Doe primary hypothyroidism today. She felt she's been more tired the last four weeks and thinks she has gained some weight

Weight: 85 Kg (previous 80)

Tests: TSH: 14 mIU/L , Total T4: 0.2 µg/dL

Diagnosis: Undertreated primary hypothyroidism.

Plan: Increasing dose of Eutroxsig to 75 mcg OD, review in 6 weeks.

Type: Test	Value:
TSH	14 IU/L ↑
T4	0.2 µg/dL ↓

And... Confirm

Activity Panel

Treatments

Levothyroxine 50

Desogestrel 75

Bilastine 20

Labs, Referrals & Others

Lab results: TSH(new) T4(new)

Referrals: 1

Ultrasound Scan: 1

Highlight | Extract | Extract ALL | Add to EHR | Edit | Add ALL | Confirm | Cancel

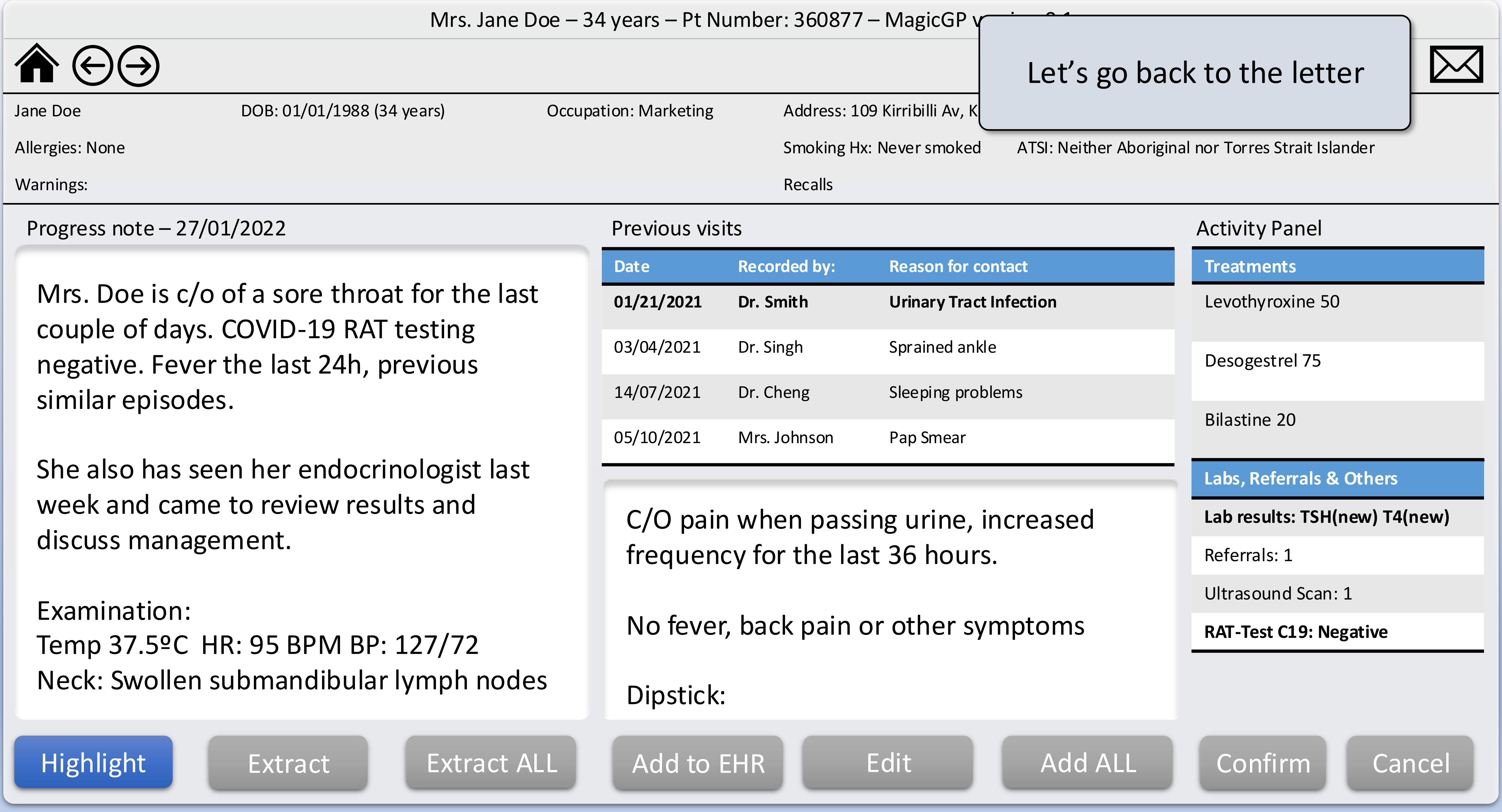
Mrs. Jane Doe – 34 years – Pt Number: 360877 – MagicGP v
Let’s go back to the letter
Jane Doe
DOB: 01/01/1988 (34 years)
Occupation: Marketing
Address: 109 Kirribilli Av, K
Allergies: None
Smoking Hx: Never smoked
ATSI: Neither Aboriginal nor Torres Strait Islander
Warnings:
Recalls
Progress note – 27/01/2022
Mrs. Doe is c/o of a sore throat for the last couple of days. COVID-19 RAT testing negative. Fever the last 24h, previous similar episodes.
She also has seen her endocrinologist last week and came to review results and discuss management.
Examination:
Temp 37.5ºC HR: 95 BPM BP: 127/72
Neck: Swollen submandibular lymph nodes
Previous visits
Date
Recorded by:
Reason for contact
01/21/2021 Dr. Smith Urinary Tract Infection
03/04/2021 Dr. Singh Sprained ankle
14/07/2021 Dr. Cheng Sleeping problems
05/10/2021 Mrs. Johnson Pap Smear
C/O pain when passing urine, increased frequency for the last 36 hours.
No fever, back pain or other symptoms
Dipstick:
Activity Panel
Treatments
Levothyroxine 50
Desogestrel 75
Bilastine 20
Labs, Referrals & Others
Lab results: TSH(new) T4(new)
Referrals: 1
Ultrasound Scan: 1
RAT-Test C19: Negative
Highlight
Extract
Extract ALL
Add to EHR
Edit
Add ALL
Confirm
Cancel

Mrs. Jane Doe – 34 years – Pt Number: 360877 – MagicGP version 0.1

Jane Doe | DOB: 01/01/1988 (34 years) | Occupation: Marketing | Address: 109 Kirribilli Av, Kirribilli NSW 2061 Ph: (02) 1234 5678

Allergies: None | Smoking Hx: Never smoked | ATSI: Neither Aboriginal nor Torres Strait Islander

Warnings: | Recalls

New Email – Received 27/01/2022

Dr. Riviera

Endocrinology Clinic
Sydney CBD

I reviewed Mrs. Doe primary hypothyroidism today. She felt she's been more tired the last four weeks and thinks she has gained some weight

Weight: 85 Kg (previous 80)

Tests: TSH: 14 mIU/L , Total T4: 0.2 µg/dL

Diagnosis: Undertreated primary hypothyroidism.

Plan: Increasing dose of Eutroxsig to 75 mcg OD, review in 6 weeks.

Mail Inbox

Date	From	Title
**27/01/2022**	**Endocrinology Cl**	**Mrs. Doe Review – Dr Riviera**
03/12/2021	Sydney Labs	Lab Results
14/07/2021	ED St. Vincent	Acute Care - Back pain
05/10/2021	Sydney Labs	Pap Smear

Now let's click Extract All instead

Activity Panel

Treatments
Levothyroxine 50
Desogestrel 75
Bilastine 20

Labs, Referrals & Others
Pending Labs: 0
Referrals: 1
Ultrasound Scan: 1

Highlight | Extract | Extract ALL | Add to EHR | Edit | Add ALL | Confirm | Cancel

Mrs. Jane Doe – 34 years – Pt Number: 360877 – MagicGP version 0.1

Jane Doe | DOB: 01/01/1988 (34 years) | Occupation: Marketing | Address: 109 Kirribilli Av, Kirribilli NSW 2061 Ph: (02) 1234 5678

Allergies: None | Smoking Hx: Never smoked | ATSI: Neither Aboriginal nor Torres Strait Islander

Warnings: | Recalls

New Email – Received 27/01/2022

Dr. Riviera — Endocrinologist clinic Sydney CBD

I re[illegible] idism
toc[illegible] e last
fou[illegible] ome
we[illegible]
We[illegible]
Tes[illegible] dL
Diagnosis: Undertreated primary hypothyroidism.
Plan: Increasing dose of Eutroxsig to 75 mcg OD, review in 6 weeks.

> Now we want to ADD ☑ the tests and the diagnosis but not the symptom (tiredness) Click Select ALL

	Type: Test	Value:
☑ ✕	TSH	14 IU/L ↑
☑ ✕	T4	0.2 µg/dL ↓
☑ ✕	Weight	85 Kg ↑
	**Type: Symptom**	
☑ ✕	Tiredness	Previous
	**Type: Diagnosis & Treatment**	
☑ ✕	Primary Hypothyroidism	Update
☑ ✕	Eutroxsig 75 mcg OD	Modify

Activity Panel

Treatments

Levothyroxine 50

Desogestrel 75

Bilastine 20

Labs, Referrals & Others

Pending Labs: 0

Referrals: 1

Ultrasound Scan: 1

Highlight | Extract | Extract ALL | Add to EHR | Edit | Select ALL | Confirm | Cancel

Mrs. Jane Doe – 34 years – Pt Number: 360877 – MagicGP version 0.1

Jane Doe | DOB: 01/01/1988 (34 years) | Occupation: Marketing | Address: 109 Kirribilli Av, Kirribilli NSW 2061 Ph: (02) 1234 5678

Allergies: None | Smoking Hx: Never smoked | ATSI: Neither Aboriginal nor Torres Strait Islander

Warnings: | Recalls

New Email – Received 27/01/2022

Dr. Riviera | Endocrinologist clinic Sydney CBD

I re[illegible] idism
toc[illegible] e last
fou[illegible] ome
we[illegible]
We[illegible]
Tes[illegible] dL
Diagnosis: Undertreated primary hypothyroidism.
Plan: Increasing dose of Eutroxsig to 75 mcg OD, review in 6 weeks.

Now unselect TSH & T4 because we have added them already
Click ✕ next to it

Type: Test	Value:
TSH	14 IU/L ↑
T4	0.2 µg/dL ↓
Weight	85 Kg ↑

Type: Symptom	
Tiredness	Previous

Type: Diagnosis & Treatment	
Primary Hypothyroidism	Update
Eutroxsig 75 mcg OD	Modify

Activity Panel

Treatments
- Levothyroxine 50
- Desogestrel 75
- Bilastine 20

Labs, Referrals & Others
- Pending Labs: 0
- Referrals: 1
- Ultrasound Scan: 1

Highlight | Extract | Extract ALL | Add to EHR | Edit | Select ALL | Confirm | Cancel

Mrs. Jane Doe – 34 years – Pt Number: 360877 – MagicGP version 0.1

Jane Doe DOB: 01/01/1988 (34 years) Occupation: Marketing Address: 109 Kirribilli Av, Kirribilli NSW 2061 Ph: (02) 1234 5678

Allergies: None Smoking Hx: Never smoked ATSI: Neither Aboriginal nor Torres Strait Islander

Warnings: Recalls

New Email – Received 27/01/2022

Dr. Riviera Endocrinologist clinic Sydney CBD

I re[illegible] idism
toc[illegible] e last
fou[illegible] ome
we[illegible]
We[illegible]
Tes[illegible] dL
Diagnosis: Undertreated primary hypothyroidism.
Plan: Increasing dose of Eutroxsig to 75 mcg OD, review in 6 weeks.

Now unselect TSH & T4 because we have added them already
Click ✕ next to it

Type: Test	Value:
~~TSH~~	~~14 IU/L~~ ↑
T4	0.2 µg/dL ↓
Weight	85 Kg ↑
**Type: Symptom**	
Tiredness	Previous
**Type: Diagnosis & Treatment**	
Primary Hypothyroidism	Update
Eutroxsig 75 mcg OD	Modify

Activity Panel

Treatments

Levothyroxine 50

Desogestrel 75

Bilastine 20

Labs, Referrals & Others

Pending Labs: 0

Referrals: 1

Ultrasound Scan: 1

Highlight | Extract | Extract ALL | Add to EHR | Edit | Select ALL | Confirm | Cancel

Mrs. Jane Doe – 34 years – Pt Number: 360877 – MagicGP version 0.1

Jane Doe | DOB: 01/01/1988 (34 years) | Occupation: Marketing | Address: 109 Kirribilli Av, Kirribilli NSW 2061 Ph: (02) 1234 5678

Allergies: None | Smoking Hx: Never smoked | ATSI: Neither Aboriginal nor Torres Strait Islander

Warnings: | Recalls

New Email – Received 27/01/2022

Dr. Riviera

Endocrinologist clinic
Sydney CBD

I re[illegible] idism
tod[illegible] e last
fou[illegible] ome
we[illegible]
We[illegible]
Tes[illegible] dL
Diagnosis: Undertreated primary hypothyroidism.
Plan: Increasing dose of Eutroxsig to 75 mcg OD, review in 6 weeks.

Now unselect TSH & T4 because we have added them already
Click ✕ next to it

	Type: Test	Value:
☑ ✕	~~TSH~~	~~14 IU/L~~
☑ ✕	~~T4~~	~~0.2 µg/dL~~
☑ ✕	Weight	85 Kg ↑

	Type: Symptom	
☑ ✕	Tiredness	Previous

	Type: Diagnosis & Treatment	
☑ ✕	Primary Hypothyroidism	Update
☑ ✕	Eutroxsig 75 mcg OD	Modify

Activity Panel

Treatments

Levothyroxine 50

Desogestrel 75

Bilastine 20

Labs, Referrals & Others

Pending Labs: 0

Referrals: 1

Ultrasound Scan: 1

Highlight | Extract | Extract ALL | Add to EHR | Edit | Select ALL | Confirm | Cancel

Mrs. Jane Doe – 34 years – Pt Number: 360877 – MagicGP version 0.1

Jane Doe DOB: 01/01/1988 (34 years) Occupation: Marketing Address: 109 Kirribilli Av, Kirribilli NSW 2061 Ph: (02) 1234 5678

Allergies: None Smoking Hx: Never smoked ATSI: Neither Aboriginal nor Torres Strait Islander

Warnings: Recalls

New Email – Received 27/01/2022

Dr. Riviera Endocrinologist clinic Sydney CBD

I re[illegible] [illegible]idism
to[illegible] [illegible]e last
fo[illegible] [illegible]ome
we[illegible]
We[illegible]
Tes[illegible] [illegible]dL
Diagnosis: Undertreated primary hypothyroidism.
Plan: Increasing dose of Eutroxsig to 75 mcg OD, review in 6 weeks.

Now Add to EHR your selection

Type: Test Value:

~~TSH~~ ~~14 IU/L~~

~~T4~~ ~~0.2 µg/dL~~

Weight 85 Kg

Type: Symptom

Tiredness Previous

Type: Diagnosis & Treatment

Primary Hypothyroidism Update

Eutroxsig 75 mcg OD Modify

Activity Panel

Treatments

Levothyroxine 50

Desogestrel 75

Bilastine 20

Labs, Referrals & Others

Pending Labs: 0

Referrals: 1

Ultrasound Scan: 1

Highlight Extract Extract ALL Add to EHR Edit Select ALL Confirm Cancel

Mrs. Jane Doe – 34 years – Pt Number: 360877 – MagicGP version 0.1

Jane Doe | DOB: 01/01/1988 (34 years) | Occupation: Marketing | Address: 109 Kirribilli Av, Kirribilli NSW 2061 Ph: (02) 1234 5678

Allergies: None | Smoking Hx: Never smoked | ATSI: Neither Aboriginal nor Torres Strait Islander

Warnings: | Recalls

New Email – Received 27/01/2022

Dr. Riviera | Endocrinologist clinic Sydney CBD

I re[illegible]idism
too[illegible]e last
fou[illegible]ome
we[illegible]
We[illegible]
Tes[illegible]dL

Confirm

Diagnosis: Undertreated primary hypothyroidism.
Plan: Increasing dose of Eutroxsig to 75 mcg OD, review in 6 weeks.

Type: Test | Value:

~~TSH~~ | ~~14 IU/L~~

~~T4~~ | ~~0.2 µg/dL~~

Weight | 85 Kg

Type: Symptom

Tiredness | Previous

Type: Diagnosis & Treatment

Primary Hypothyroidism | Update

Eutroxsig 75 mcg OD | Modify

Activity Panel

Treatments

Levothyroxine 75 (changed)

Desogestrel 75

Bilastine 20

Labs, Referrals & Others

Weight(new)

Symptom – Tiredness – update

Diagnoses: Primary Hypothyroid. - update

Highlight | Extract | Extract ALL | Add to EHR | Edit | Select ALL | Confirm | Cancel

Mrs. Jane Doe – 34 years – Pt Number: 360877 – MagicGP version 0.1

Jane Doe | DOB: 01/01/1988 (34 years) | Occupation: Marketing | Address: 109 Kirribilli Av, Kirribilli NSW 2061 Ph: (02) 1234 5678

Allergies: None | Smoking Hx: Never smoked | ATSI: Neither Aboriginal nor Torres Strait Islander

Warnings: | Recalls

Progress note – 27/01/2022

Mrs. Doe is c/o of a sore throat for the last couple of days. COVID-19 RAT testing negative. Fever the last 24h, previous similar episodes.

She also has seen her endocrinologist last week and came to review results and discuss management.

Examination:
Temp 37.5ºC HR: 95 BPM BP: 127/72
Neck: Swollen submandibular lymph nodes

Previous visits

Date	Recorded by:	Reason for contact
**01/21/2021**	**Dr. Smith**	**Urinary Tract Infection**
03/04/2021	Dr. Singh	Sprained ankle
14/07/2021	Dr. Cheng	Sleeping problems
05/10/2021	Mrs. Johnson	Pap Smear

C/O pain when passing urine, increased frequency for the last 36 hours.

No fever, back pain or other symptoms

Dipstick:

Activity Panel

Treatments
**Levothyroxine 75 (changed)**
Desogestrel 75
Bilastine 20

Labs, Referrals & Others
**Weight(new)**
**Symptom – Tiredness – update**
**Diagnoses: Primary Hypothyroid. - update**

Highlight | Extract | Extract ALL | Add to EHR | Edit | Add ALL | Confirm | Cancel

Mrs. Jane Doe – 34 years – Pt Number: 360877 – MagicGP version 0.1

Jane Doe | DOB: 01/01/1988 (34 years) | Occupation: Marketing | Address: 109 Kirribilli Av, Kirribilli NSW 2061 Ph: (02) 1234 5678

Allergies: None | Smoking Hx: Never smoked | ATSI: Neither Aboriginal nor Torres Strait Islander

Warnings: | Recalls

Progress note – 27/01/2022

Examination:
Temp 37.5ºC HR: 95 BPM BP: 127/72
Neck: Swollen submandibular lymph nodes
ENT: Swollen purulent tonsils, ears nil
D: Tonsillitis
Plan: Penicillin 500mg BID for 10 days plus Panadol as required.
Review again if worsening.

Previous visits

Dat
01/
03/
14/
05/

Now let's do something different...
Click "Add ALL"

C/O pain when passing urine, increased frequency for the last 36 hours.

No fever, back pain or other symptoms

Dipstick:

Activity Panel

Treatments
**Levothyroxine 75 (changed)**
Desogestrel 75
Bilastine 20

Labs, Referrals & Others
**Weight(new)**
**Symptom – Tiredness – update**
**Diagnoses: Primary Hypothyroid. - update**

Highlight | Extract | Extract ALL | Add to EHR | Edit | Add ALL | Confirm | Cancel

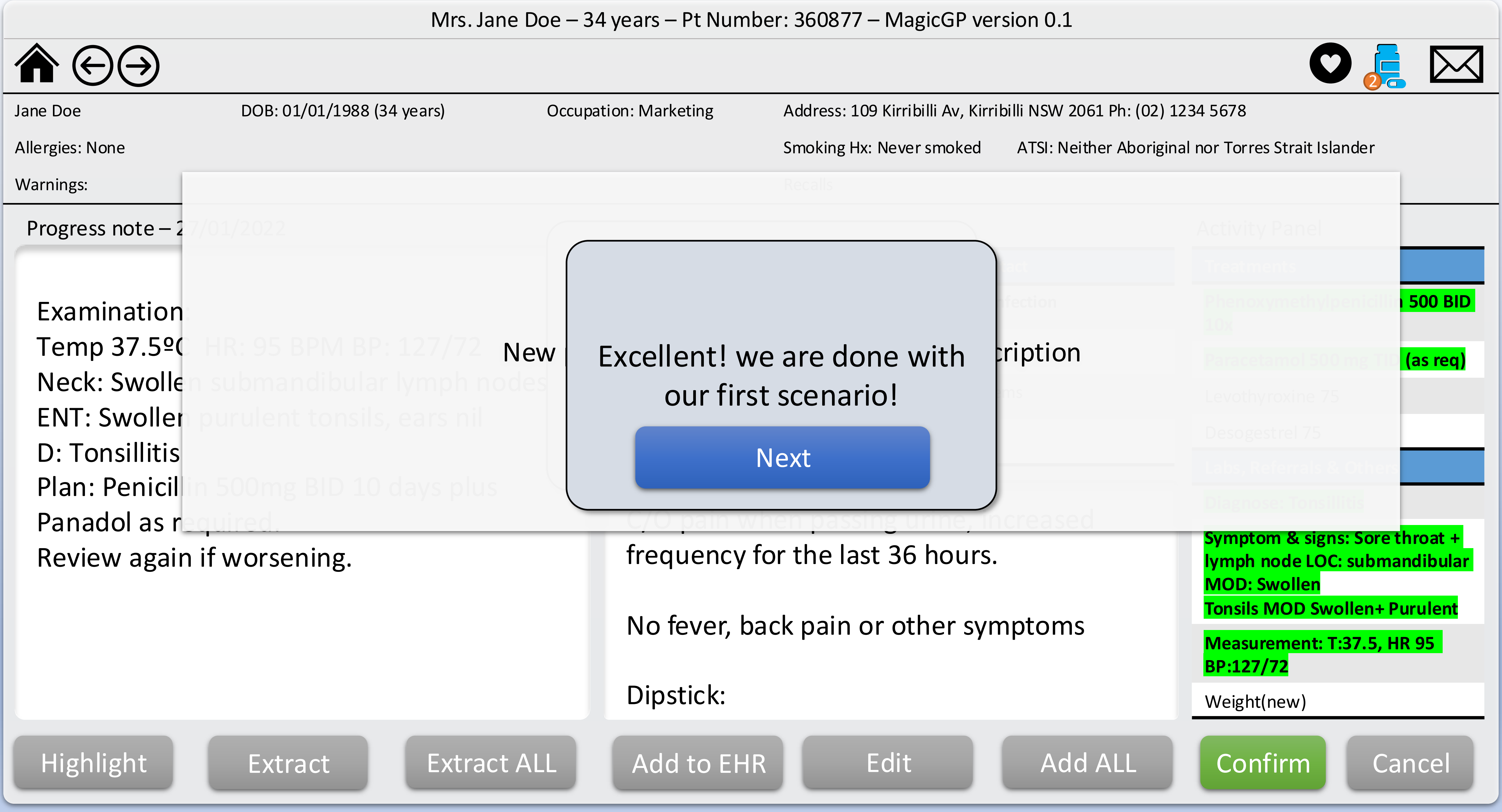
Mrs. Jane Doe – 34 years – Pt Number: 360877 – MagicGP version 0.1
2
Jane Doe
DOB: 01/01/1988 (34 years)
Occupation: Marketing
Address: 109 Kirribilli Av, Kirribilli NSW 2061 Ph: (02) 1234 5678
Allergies: None
Smoking Hx: Never smoked
ATSI: Neither Aboriginal nor Torres Strait Islander
Warnings:
Progress note – 2
Examination
Temp 37.5ºC
Neck: Swolle
ENT: Swollen
D: Tonsillitis
Plan: Penicil
Panadol as r
Review again if worsening.
New
cription
Excellent! we are done with our first scenario!
Next
frequency for the last 36 hours.
No fever, back pain or other symptoms
Dipstick:
500 BID
(as req)
Symptom & signs: Sore throat + lymph node LOC: submandibular MOD: Swollen
Tonsils MOD Swollen+ Purulent
Measurement: T:37.5, HR 95 BP:127/72
Weight(new)
Highlight
Extract
Extract ALL
Add to EHR
Edit
Add ALL
Confirm
Cancel

Second Scenario

You are a Locum Dr. You have been working in several clinics the last months. This is one of your usual ones. Today you are reviewing a chronic patient that usually is followed by another Dr. in the clinic who isn't there.

Mr. Morris Scott – 67 years – Pt Number: 441947 – MagicGP version 0.1

Morris Scott | DOB: 03/03/1955 (67years) | Occupation: Marketing | Address: 109 Kirribilli Av, Kirribilli NSW 2061 Ph: (02) 2345 6789

Allergies: Penicillin | Smoking Hx: Ex-Smoker | ATSI: Neither Aboriginal nor Torres Strait Islander

Warnings: Noncompliant with treatment | Recalls

Progress note – 14/06/2022

Mr. Morris is c/o tiredness that has been ongoing for the last 4 months, he has seen a number of GPs and have made adjustments to his treatments, but he feels as tired or perhaps even more. He has not lost appetite or weight. He says he has also done some bloods recently and would like to see results.

Previous visits

Date	Recorded by:	Reason for contact
**01/21/2021**	**Dr. Smith**	**Urinary Tract Infection**
03/04/2021	Dr. Singh	Sprained ankle
14/07/2021	Dr. Cheng	Sleeping problems
05/10/2021	Mrs. Johnson	Pap Smear

Activity Panel

Treatments
Inhaler
Antihiper
Metformin 850mg 1-1-1

Labs, Referrals & Others
Weight(new)
Symptom – Tiredness – update
Diagnoses: Primary Hypothyroid. - update

Highlight | Extract | Extract ALL | Add to EHR | Edit | Add ALL | Confirm | Cancel

Mr. Morris Scott – 67 years – Pt Number: 441947 – MagicGP version 0.1

Let's click "results" in the text

Morris Scott

Allergies: Penicillin

Warnings: Noncom

pation: Marketing

Address: 109 Kirribilli Av, Kirribilli NSW 2061 Ph: (02) 2345 6789

Smoking Hx: Ex-Smoker

ATSI: Neither Aboriginal nor Torres Strait Islander

Recalls

Progress note

Mr. Morris is c/o tiredness that has been ongoing for the last 4 months, he has seen a number of GPs and have made adjustments to his treatments, but he feels as tired or perhaps even more. He has not lost appetite or weight. He says he has also done some bloods recently and would like to see **results**.

Previous visits

Date	Recorded by:	Reason for contact
**01/21/2021**	**Dr. Smith**	**Urinary Tract Infection**
03/04/2021	Dr. Singh	Sprained ankle
14/07/2021	Dr. Cheng	Sleeping problems
05/10/2021	Mrs. Johnson	Pap Smear

Activity Panel

Treatments
Inhaler
Antihiper
Metformin 850mg 1-1-1

Labs, Referrals & Others
Weight(new)
Symptom – Tiredness – update
Diagnoses: Primary Hypothyroid. - update

Highlight | Extract | Extract ALL | Add to EHR | Edit | Add ALL | Confirm | Cancel

Mr. Morris Scott – 67 years – Pt Number: 441947 – MagicGP version 0.1

Let's click "results" in the text

Morris Scott

pation: Marketing

Address: 109 Kirribilli Av, Kirribilli NSW 2061 Ph: (02) 2345 6789

Allergies: Penicillin

Smoking Hx: Ex-Smoker

ATSI: Neither Aboriginal nor Torres Strait Islander

Warnings: Noncom

Recalls

Progress note

Mr. Morris is c/o tiredness that has been ongoing for the last 4 months, he has seen a number of GPs and have made adjustments to his treatments, but he feels as tired or perhaps even more. He has not lost appetite or weight. He says he has also done some bloods recently and would like to see **results**.

Previous visits

Date	Recorded by:	Reason for contact
**01/21/2021**	**Dr. Smith**	**Urinary Tract Infection**
03/04/2021	Dr. Singh	Sprained ankle
14/07/2021	Dr. Cheng	Sleeping problems
05/10/2021	Mrs. Johnson	Pap Smear

Activity Panel

Treatments
Inhaler
Antihiper
Metformin 850mg 1-1-1

Labs, Referrals & Others
Weight(new)
Symptom – Tiredness – update
Diagnoses: Primary Hypothyroid. - update

Select | Find | Summarize | SUM ALL | Edit | Add ALL | Confirm | Cancel

Mr. Morris Scott – 67 years – Pt Number: 441947 – MagicGP version 0.1

Morris Scott

...pation: Marketing

Address: 109 Kirribilli Av, Kirribilli NSW 2061 Ph: (02) 2345 6789

Allergies: Penicillin

Smoking Hx: Ex-Smoker

ATSI: Neither Aboriginal nor Torres Strait Islander

Warnings: Noncom...

Recalls

Let’s click “results” in the text

Progress note...

Mr. Morris is c/o tiredness that has been ongoing for the last 4 months, he has seen a number of GPs and have made adjustments to his treatments, but he feels as tired or perhaps even more. He has not lost appetite or weight. He says he has also done some bloods recently and would like to see **results**.

Previous visits

Date	Recorded by:	Reason for contact
01/21/2021	Dr. Smith	Urinary Tract Infection
03/04/2021	Dr. Singh	Sprained ankle
14/07...		Slee...
05/10/2021	Mrs. Johnson	Pap Smear

Found **3 new** “results” and 34 viewed “results”

View new | View all

Summarize new | Summarize all

Activity Panel

Treatments

- Symbicort Turbuhaler 200/6 1-0-1
- Enalapril 10mg 1-0-1
- Metformin 850mg 1-1-1

Labs, Referrals & Others

- Weight(new)
- Symptom – Tiredness – update
- Diagnoses: Primary Hypothyroid. - update

Select | Find | Summarize | SUM ALL | Edit | Add ALL | Confirm | Cancel

Mr. Morris Scott – 67 years – Pt Number: 441947 – MagicGP version 0.1

Morris Scott | DOB: 03/03/1955 (67years) | Occupation: Marketing | Address: 109 Kirribilli Av, Kirribilli NSW 2061 Ph: (02) 2345 6789

Allergies: Penicillin | Smoking Hx: Ex-Smoker | ATSI: Neither Aboriginal nor Torres Strait Islander

Warnings: Noncompliant with treatment | Recalls

Respiratory Clinic– 13/06/2022

Respiratory Clinic Sydney CBD

Test results for Mr. Scott:

Test	Trial 1	Trial 2	Trial 3	Best Test
FVC	5.20	5.30	5.35	5.35
FEV_1	4.41	4.35	4.36	4.41
FEV_1/FVC	85	82	82	82
FEF_{25-75}	3.87	3.92	3.94	3.94
FEF_{50}	3.99	3.95	3.41	3.41
FEF_{25}	1.97	1.95	1.89	1.89
PEF	8.39	9.89	9.44	9.89

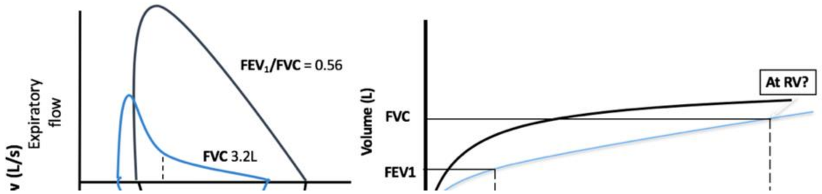

Results

Date	Provider	Test Name
**13/06/2022**	**Respiratory Clinic**	**Spirometry Results**
**14/0**		
**14/0**		
05/1		

Now let's click Summarize

Activity Panel

Treatments

- Symbicort Turbuhaler 200/6 1-0-1
- Enalapril 10mg 1-0-1
- Metformin 850mg 1-1-1

Labs, Referrals & Others

- Weight(new)
- Symptom – Tiredness – update
- Diagnoses: Primary Hypothyroid. - update

Select | Find | Summarize | SUM ALL | Edit | Add ALL | Confirm | Cancel

Mr. Morris Scott – 67 years – Pt Number: 441947 – MagicGP version 0.1

Morris Scott | DOB: 03/03/1955 (67years) | Occupation: Marketing | Address: 109 Kirribilli Av, Kirribilli NSW 2061 Ph: (02) 2345 6789

Allergies: Penicillin | Smoking Hx: Ex-Smoker | ATSI: Neither Aboriginal nor Torres Strait Islander

Warnings: Noncompliant with treatment | Recalls

Respiratory Clinic– 13/06/2022

Respiratory Clinic Sydney CBD
Test results for Mr. Scott:

Test	Trial 1	Trial 2	Trial 3	Best Test
FVC	5.20	5.30	5.35	5.35
FEV_1	4.41	4.35	4.36	4.41
FEV_1/FVC	85	82	82	82
FEF_{25-75}	3.87	3.92	3.94	3.94
FEF_{50}	3.99	3.95	3.41	3.41
FEF_{25}	1.97	1.95	1.89	1.89
PEF	8.39	9.89	9.44	9.89

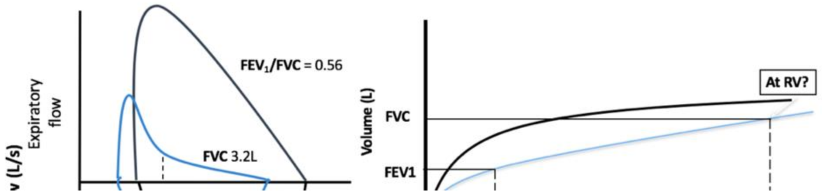

Mr. Scott spirometry shows a decrease in FEV1 compared to previous one from 12/02/2015. It currently qualifies as severe COPD following GOLD criteria.

Add to progress note | Lay | Cancel

Activity Panel

Treatments

- Symbicort Turbuhaler 200/6 1-0-1
- Enalapril 10mg 1-0-1
- Metformin 850mg 1-1-1

Now let's click Summarize

Select | Find | Summarize | SUM ALL | Edit | Add ALL | Confirm | Cancel

Mr. Morris Scott – 67 years – Pt Number: 441947 – MagicGP version 0.1

Morris Scott DOB: 03/03/1955 (67years) Occupation: Retired Plumber Address: 109 Kirribilli Av, Kirribilli NSW 2061 Ph: (02) 2345 6789

Allergies: Penicillin Smoking Hx: Ex-Smoker ATSI: Neither Aboriginal nor Torres Strait Islander

Warnings: Noncompliant with treatment Recalls

Progress note – 14/06/2022

Mr. Scott is c/o tiredness that has been ongoing for the last 4 months, he has seen a number of GPs and have made adjustments to his treatments, but he feels as tired or perhaps even more. He has not lost appetite or weight. He says he has also done some bloods recently and would like to see **results**.

Mr. Scott spirometry (13/06/2022) shows a decrease in FEV1 (45%) compared to previous one (-12%) (12/02/2015). It currently qualifies as severe COPD following GOLD criteria.

Previous visits

Date	Recorded by:	Reason for contact
**01/21/2021**	**Dr. Smith**	**Urinary Tract Infection**
03/04		
14/07		
05/10		

Let's click "results" in the text

Activity Panel

Treatments
Inhaler
Antihiper
Metformin 850mg 1-1-1

Labs, Referrals & Others
Weight(new)
Symptom – Tiredness – update
Diagnoses: Primary Hypothyroid. - update

Select Find Summarize SUM ALL Edit Add ALL Confirm Cancel

Mr. Morris Scott – 67 years – Pt Number: 441947 – MagicGP version 0.1

Morris Scott (he/him) DOB: 03/03/1955 (67years) Occupation: Retired Plumber Address: 109 Kirribilli Av, Kirribilli NSW 2061 Ph: (02) 2345 6789

Allergies: Penicillin Smoking Hx: Ex-Smoker ATSI: Neither Aboriginal nor Torres Strait Islander

Warnings: Noncompliant with treatment Recalls

Progress note – 14/06/2022

Mr. Scott is c/o tiredness that has been ongoing for the last 4 months, he has seen a number of GPs and have made adjustments to his treatments, but he feels as tired or perhaps even more. He has not lost appetite or weight. He says he has also done some bloods recently and would like to see **results**.

Mr. Scott spirometry (13/06/2022) shows a decrease in FEV1 (45%) compared to previous one (-12%) (12/02/2015). It currently qualifies as severe COPD following GOLD criteria.

Previous visits

Date	Recorded by:	Reason for contact
**01/21/2021**	**Dr. Smith**	**Urinary Tract Infection**
03/04[illegible]		
14/07[illegible]		
05/10[illegible]		

Mr. Scott was wondering about his “other” results and wonder if he can switch back to previous antihypertensive, the one he used years ago…

Activity Panel

Treatments

Inhaler

Enalapril 20mg 1-0-1

Metformin 850mg 1-1-1

Labs, Referrals & Others

Weight(new)

Symptom – Tiredness – update

Diagnoses: Primary Hypothyroid. - update

Select | Find | Summarize | SUM ALL | Edit | Add ALL | Confirm | Cancel

Mr. Morris Scott – 67 years – Pt Number: 441947 – MagicGP version 0.1

Morris Scott (he/him) DOB: 03/03/1955 (67years) Occupation: Retired Plumber Address: 109 Kirribilli Av, Kirribilli NSW 2061 Ph: (02) 2345 6789

Allergies: Penicillin Smoking Hx: Ex-Smoker ATSI: Neither Aboriginal nor Torres Strait Islander

Warnings: Noncompliant with treatment Recalls

Progress note – 14/06/2022

Mr. Scott is c/o tiredness that has been ongoing for the last 4 months, he has seen a number of GPs and have made adjustments to his treatments, but he feels as tired or perhaps even more. He has not lost appetite or weight. He says he has also done some bloods recently and would like to see **results**.

Mr. Scott spirometry (13/06/2022) shows a decrease in FEV1 (45%) compared to previous one (-12%) (12/02/2015). It currently qualifies as severe COPD following GOLD criteria.

Previous visits

Date	Recorded by:	Reason for contact
**01/21/2021**	**Dr. Smith**	**Urinary Tract Infection**
03/04[illegible]		
14/07[illegible]		
05/10[illegible]		

Let's click again in "results" and now also click in "enalapril"

Activity Panel

Treatments
Inhaler
Enalapril 20mg 1-0-1
Metformin 850mg 1-1-1

Labs, Referrals & Others
Weight(new)
Symptom – Tiredness – update
Diagnoses: Primary Hypothyroid. - update

Select | Find | Summarize | SUM ALL | Edit | Add ALL | Confirm | Cancel

Mr. Morris Scott – 67 years – Pt Number: 441947 – MagicGP version 0.1

Morris Scott (he/him) DOB: 03/03/1955 (67years) Occupation: Retired Plumber Address: 109 Kirribilli Av, Kirribilli NSW 2061 Ph: (02) 2345 6789

Allergies: Penicillin Smoking Hx: Ex-Smoker ATSI: Neither Aboriginal nor Torres Strait Islander

Warnings: Noncompliant with treatment Recalls

Progress note – 14/06/2022

Mr. Scott is c/o tiredness that has been ongoing for the last 4 months, he has seen a number of GPs and have made adjustments to his treatments, but he feels as tired or perhaps even more. He has not lost appetite or weight. He says he has also done some bloods recently and would like to see **results**.

Mr. Scott spirometry (13/06/2022) shows a decrease in FEV1 (45%) compared to previous one (-12%) (12/02/2015). It currently qualifies as severe COPD following GOLD criteria.

Previous visits

Date	Recorded by:	Reason for contact
**01/21/2021**	**Dr. Smith**	**Urinary Tract Infection**
03/04[illegible]		
14/07[illegible]		
05/10[illegible]		

Let's click again in "results" and now also click in "enalapril"

Activity Panel

Treatments

- Inhaler
- **Enalapril 20mg 1-0-1**
- Metformin 850mg 1-1-1

Labs, Referrals & Others

- Weight(new)
- Symptom – Tiredness – update
- Diagnoses: Primary Hypothyroid. - update

Select | Find | Summarize | SUM ALL | Edit | Add ALL | Confirm | Cancel

Mr. Morris Scott – 67 years – Pt Number: 441947 – MagicGP version 0.1

Morris Scott (he/him) DOB: 03/03/1955 (67years) Occupation: Retired Plumber Address: 109 Kirribilli Av, Kirribilli NSW 2061 Ph: (02) 2345 6789

Allergies: Penicillin Smoking Hx: Ex-Smoker ATSI: Neither Aboriginal nor Torres Strait Islander

Warnings: Noncompliant with treatment Recalls

Progress note – 14/06/2022

Mr. Scott is c/o tiredness that has been ongoing for the last 4 months, he has seen a number of GPs and have made adjustments to his treatments, but he feels as tired or perhaps even more. He has not lost appetite or weight. He says he has also done some bloods recently and would like to see **results**.

Mr. Scott spirometry (13/06/2022) shows a decrease in FEV1 (45%) compared to previous one (-12%) (12/02/2015). It currently qualifies as severe COPD following GOLD criteria.

Previous visits

Date	Recorded by:	Reason for contact
**01/21/2021**	**Dr. Smith**	**Urinary Tract Infection**
03/04[illegible]		
14/07[illegible]		
05/10[illegible]		

Now let's click summarize

Activity Panel

Treatments

Inhaler

Enalapril 20mg 1-0-1

Metformin 850mg 1-1-1

Labs, Referrals & Others

Weight(new)

Symptom – Tiredness – update

Diagnoses: Primary Hypothyroid. - update

Select Find Summarize SUM ALL Edit Add ALL Confirm Cancel

Mr. Morris Scott – 67 years – Pt Number: 441947 – MagicGP version 0.1

Morris Scott (he/him) DOB: 03/03/1955 (67years) Occupation: Retired Plumber Address: 109 Kirribilli Av, Kirribilli NSW 2061 Ph: (02) 2345 6789

Allergies: Penicillin Smoking Hx: Ex-Smoker ATSI: Neither Aboriginal nor Torres Strait Islander

Warnings: Noncompliant with treatment

Progress note – 14/06/2022

Mr. Scott is c/o tiredness that has been ongoing for the last 4 months, he has seen a number of GPs and have made adjustments to his treatments, but he feels as tired or perhaps even more. He has not lo[illegible] or weight. He says he has also done some bloods recently and would like to see results.

Mr. Scott spirometry (13/06/2022) shows a decrease in FEV1 (45%) compared to previous one (-12%) (12/02[illegible] currently qualifies as severe COPD following GOLD criteria.

Here is a summary of **2 new "results"** and 35 previous results.
Showing **new "results"** summary:

- Blood test shows a slight decrease of haemoglobin (11.4) with increase MCV and a total cholesterol of 180 (previous 171)
- Results from Colorectal Cancer screening were **negative**

Add to progress note | Summarize all results

There are no new changes to "enalapril" do you want to summarize past changes to enalapril or to hypertension medication instead?

past – enalapril | past – hypertension

Activity Panel

Treatments

Inhaler

Enalapril 20mg 1-0-1

Metformin 850mg 1-1-1

Labs, Referrals & Others

Weight(new)

Symptom – Tiredness – update

Diagnoses: Primary Hypothyroid. - update

Select | Find | Summarize | SUM ALL | Edit | Add ALL | Confirm | Cancel

Let's first add the rest of new results to progress note

Mr. Morris Scott – 67 years – Pt Number: 441947 – MagicGP version 0.1

1. Here is a summary of **2 new "results"** and 35 previous results.
Showing **new "results"** summary:

- Blood test shows a slight decrease of haemoglobin (11.4) with increase MCV and a total cholesterol of 180 (previous 171)
- Results from Colorectal Cancer screening were **negative**

Add to progress note | Summarize all results

2. There are no new changes to "enalapril" do you want to summarize past changes to enalapril or to hypertension medication instead?

past – enalapril | past – hypertension

Mr. Scott is c/o tiredness that has been ongoing for the last 4 months, he has seen a number of GPs and have made adjustments to his treatments, but he feels as tired or perhaps even more. He has not lo[illegible] or weight. He says he has also done some bloods recently and would like to see results.

Mr. Scott spirometry (13/06/2022) shows a decrease in FEV1 (45%) compared to previous one (-12%) (12/02[illegible] currently qualifies as severe COPD following GOLD criteria.

Activity Panel

Treatments

Inhaler

Enalapril 20mg 1-0-1

Metformin 850mg 1-1-1

Labs, Referrals & Others

Weight(new)

Symptom – Tiredness – update

Diagnoses: Primary Hypothyroid. - update

Select | Find | Summarize | SUM ALL | Edit | Add ALL | Confirm | Cancel

Then we are going to investigate further past medication…

Mr. Morris Scott – 67 years – Pt Number: 441947 – MagicGP version 0.1

5 (67years) Occupation: Retired Plumber Address: 109 Kirribilli Av, Kirribilli NSW 2061 Ph: (02) 2345 6789

Smoking Hx: Ex-Smoker ATSI: Neither Aboriginal nor Torres Strait Islander

Recalls

1. Here is a summary of **no new "results"** and 37 previous results.
   - Blood test shows a slight decrease of haemoglobin (11.4) with increase MCV and a total cholesterol of 180 (previous 171)
   - Results from Colorectal Cancer screening were **negative**

Add to progress note | Summarize all results

2. There are no new changes to "enalapril" do you want to summarize past changes to enalapril or to hypertension medication instead?

past – enalapril | past – hypertension

that has been ongoing for the last 4 months, he has seen a but Mr. Scott spirometry (13/06/2022) shows a decrease in FEV1 (45%) compared to previous one (-12%) (12/02/20… currently qualifies as severe COPD following GOLD criteria

Blood test shows a slight decrease of haemoglobin (11.4) with increase MCV and a total cholesterol of 180 (pre…

Results from Colorectal Cancer screening were negative

Previous visits

Date	Recorded by:	Reason for contact
01/21/2021	Dr. Smith	Urinary Tract Infection
03/04/2021	Dr. Singh	Sprained ankle
	Dr. Cheng	
05/10/2021	Mrs. Johnson	Pap Smear

Activity Panel

Treatments

Inhaler

Enalapril 20mg 1-0-1

Metformin 850mg 1-1-1

Labs, Referrals & Others

Weight(new)

Symptom – Tiredness – update

Diagnoses: Primary Hypothyroid. - update

Select | Find | Summarize | SUM ALL | Edit | Add ALL | Confirm | Cancel

Then we are going to investigate further past medication…

Mr. Morris Scott – 67 years – Pt Number: 441947 – MagicGP version 0.1

5 (67years) Occupation: Retired Plumber Address: 109 Kirribilli Av, Kirribilli NSW 2061 Ph: (02) 2345 6789

Smoking Hx: Ex-Smoker ATSI: Neither Aboriginal nor Torres Strait Islander

Recalls

Showing **past – hypertension** medication:

- **Enalapril 20mg BD** started on 20/06/2021 an increase dose from
- **Enalapril 10 mg BD** started on 12/04/2019 change from previous drug **Atenolol 50 mg daily** (reason for change worsening COPD and insufficient blood pressure control)
- **Atenolol 50 mg** daily was started on 04/03/2012 and increase from previous dose
- **Atenolol 25 mg** started on 20/05/1999 (first diagnose of hypertension on record)

Free-text summary | Add to progress note

Print | Medication | Cancel

Mr. Scott is c/o tiredness that has been ongoing for the last 4 months, he has seen a number of GPs and have made adjustments to his treatments, but he feels as tired or perhaps even more. He has not lost appetite or weight. He says he has also done some bloods recently and would like to see results.

Mr. Scott spirometry (13/06/2022) shows a decrease in FEV1 (45%) compared to previous one (-12%) (12/02/2015). It currently qualifies as severe COPD following GOLD criteria.

Activity Panel

Treatments

Inhaler

Enalapril 20mg 1-0-1

Metformin 850mg 1-1-1

Labs, Referrals & Others

Weight(new)

Symptom – Tiredness – update

Diagnoses: Primary Hypothyroid. - update

Select | Find | Summarize | SUM ALL | Edit | Add ALL | Confirm | Cancel

Then we are going to investigate further past medication…

Mr. Morris Scott – 67 years – Pt Number: 441947 – MagicGP version 0.1

5 (67years) Occupation: Retired Plumber Address: 109 Kirribilli Av, Kirribilli NSW 2061 Ph: (02) 2345 6789

Smoking Hx: Ex-Smoker ATSI: Neither Aboriginal nor Torres Strait Islander

Recalls

Showing **past – hypertension** medication (free-text summary):

Mr. Scott currently takes Enalapril 20mg twice daily started the 20th June 2021. Dose was increased from previous Enalapril 10 mg twice daily due to poor control which was started on 12th April 2019. Previous to enalapril Mr. Scott was taking Atenolol 50mg daily and the reason for change was due to worsening COPD and poor hypertension control. Atenolol 50 mg was started 4th March 2012, an increase from previous dose Atenolol 25mg which was started 20th May 1999 and is the first treatment for hypertension in the record.

Switch to tabulated text | Add to progress note | Copy

Other… | Print | Medication | Cancel

Mr. Scott is c/o tiredness that has been ongoing for the last 4 months, he has seen a number of GPs and have made adjustments to his treatments, but he feels as tired or perhaps even more. He has not lost appetite or weight. He says he has also done some bloods recently and would like to see results.

Mr. Scott spirometry (13/06/2022) shows a decrease in FEV1 (45%) previous one (-12%) (12/02/2015). It currently qualifies as severe GOLD criteria.

Date | Recorded by: | Reason for contact

03/04/2021 Dr. Singh Sprained ankle

14/07/2021 Dr. Cheng Sleeping problems

Activity Panel

Treatments

...er

Enalapril 20mg 1-0-1

Metformin 850mg 1-1-1

Labs, Referrals & Others

Weight(new)

Symptom – Tiredness – update

Diagnoses: Primary Hypothyroid. update

Select | Find | Summarize | SUM ALL | Edit | Add ALL | Confirm | Cancel

Now that you have explained to Mr. Scott why you can't change him back to his previous medications hit cancel to get back to the main window

Mr. Morris Scott – 67 years – Pt Number: 441947 – MagicGP version 0.1

Mr. Scott is c/o tiredness that has been ongoing for the last 4 months, he has seen a number of GPs and have made adjustments to his treatments, but he feels as tired or perhaps even more. He has not lost appetite or weight. He says he has also done some bloods recently and would like to see results.

Mr. Scott spirometry (13/06/2022) shows a decrease in FEV1 (45%) previous one (-12%) (12/02/2015). It currently qualifies as severe GOLD criteria.

Showing **past – hypertension** medication (free-text summary):

Mr. Scott currently takes Enalapril 20mg twice daily started the 20th June 2021. Dose was increased from previous Enalapril 10 mg twice daily due to poor control which was started on 12th April 2019. Previous to enalapril Mr. Scott was taking Atenolol 50mg daily and the reason for change was due to worsening COPD and poor hypertension control. Atenolol 50 mg was started 4th March 2012, an increase from previous dose Atenolol 25mg which was started 20th May 1999 and is the first treatment for hypertension in the record.

Switch to tabulated text | Add to progress note | Copy

Other… | Print | Medication | Cancel

Occupation: Retired Plumber | Address: 109 Kirribilli Av, Kirribilli NSW 2061 Ph: (02) 2345 6789

Smoking Hx: Ex-Smoker | ATSI: Neither Aboriginal nor Torres Strait Islander

Recalls

Activity Panel

Treatments

Enalapril 20mg 1-0-1

Metformin 850mg 1-1-1

Referrals & Others

Weight(new)

Symptom – Tiredness – update

Diagnoses: Primary Hypothyroid. update

Select | Find | Summarize | SUM ALL | Edit | Add ALL | Confirm | Cancel

Mr. Morris Scott – 67 years – Pt Number: 441947 – MagicGP version 0.1

Morris Scott (he/him) DOB: 03/03/1955 (67years) Occupation: Retired Plumber Address: 109 Kirribilli Av, Kirribilli NSW 2061 Ph: (02) 2345 6789

Allergies: Penicillin Smoking Hx: Ex-Smoker ATSI: Neither Aboriginal nor Torres Strait Islander

Warnings: Noncompliant with treatment Recalls

Progress note – 14/06/2022

Mr. Scott spirometry (13/06/2022) shows a decrease in FEV1 (45%) compared to previous one (-12%) (12/02/2015). It currently qualifies as severe COPD following GOLD criteria.

Blood test shows a slight decrease of haemoglobin (11.4) with increase in MCV and a total cholesterol of 180 (previous 171)

Results from Colorectal Cancer screening were negative

Previous visits

Date	Recorded by:	Reason for contact
**01/21/2021**	**Dr. Smith**	**Urinary Tract Infection**
03/04/2021	Dr. Singh	Sprained ankle
14/07/2021	Dr. Cheng	Sleeping problems
05/10/2021	Mrs. Johnson	Pap Smear

You continue seeing Mr. Scott an do the adjustments to his treatment that he requires but before he leaves…

Activity Panel

Treatments
Inhaler
**Enalapril 20mg 1-0-1**
Metformin 850mg 1-1-1

Labs, Referrals & Others
Weight(new)
Symptom – Tiredness – update
Diagnoses: Primary Hypothyroid. - update

Select Find Summarize SUM ALL Edit Add ALL Confirm Cancel

Mr. Morris Scott – 67 years – Pt Number: 441947 – MagicGP version 0.1

Morris Scott (he/him) DOB: 03/03/1955 (67years) Occupation: Retired Plumber Address: 109 Kirribilli Av, Kirribilli NSW 2061 Ph: (02) 2345 6789

Allergies: Penicillin Smoking Hx: Ex-Smoker ATSI: Neither Aboriginal nor Torres Strait Islander

Warnings: Noncompliant with treatment Recalls

Progress note – 14/06/2022

Mr. Scott spirometry (13/06/2022) shows a decrease in FEV1 (45%) compared to previous one (-12%) (12/02/2015). It currently qualifies as severe COPD following GOLD criteria.

Blood test shows a slight decrease of haemoglobin (11.4) with increase in MCV and a total cholesterol of 180 (previous 171)

Results from Colorectal Cancer screening were negative

Previous visits

Date	Recorded by:	Reason for contact
**01/21/2021**	**Dr. Smith**	**Urinary Tract Infection**
03/04/2021	Dr. Singh	Sprained ankle
14/07/2021	Dr. Cheng	Sleeping problems
05/10/2021	Mrs. Johnson	Pap Smear

He remembers that he is going on a Pacific Cruise, he’s leaving tomorrow and needs to carry a letter with his past medical history …

Activity Panel

Treatments
Inhaler
**Enalapril 20mg 1-0-1**
Metformin 850mg 1-1-1

Labs, Referrals & Others
Weight(new)
Symptom – Tiredness – update
Diagnoses: Primary Hypothyroid. - update

Select | Find | Summarize | SUM ALL | Edit | Add ALL | Confirm | Cancel

Mr. Morris Scott – 67 years – Pt Number: 441947 – MagicGP version 0.1

Morris Scott (he/him) DOB: 03/03/1955 (67years) Occupation: Retired Plumber Address: 109 Kirribilli Av, Kirribilli NSW 2061 Ph: (02) 2345 6789

Allergies: Penicillin Smoking Hx: Ex-Smoker ATSI: Neither Aboriginal nor Torres Strait Islander

Warnings: Noncompliant with treatment Recalls

Progress note – 14/06/2022

Mr. Scott spirometry (13/06/2022) shows a decrease in FEV1 (45%) compared to previous one (-12%) (12/02/2015). It currently qualifies as severe COPD following GOLD criteria.

Blood test shows a slight decrease of haemoglobin (11.4) with increase in MCV and a total cholesterol of 180 (previous 171)

Results from Colorectal Cancer screening were negative

Previous visits

Date	Recorded by:	Reason for contact
**01/21/2021**	**Dr. Smith**	**Urinary Tract Infection**
03/04/2021	Dr. Singh	Sprained ankle
14/07/2021	Dr. Cheng	Sleeping problems
05/10/2021	Mrs. Johnson	Pap Smear

You are already delayed with the long consultation from Mr. Scott, but less see if we can help him get on the cruise

Activity Panel

Treatments
Inhaler
**Enalapril 20mg 1-0-1**
Metformin 850mg 1-1-1

Labs, Referrals & Others
Weight(new)
Symptom – Tiredness – update
Diagnoses: Primary Hypothyroid. - update

Select | Find | Summarize | SUM ALL | Edit | Add ALL | Confirm | Cancel

Mr. Morris Scott – 67 years – Pt Number: 441947 – MagicGP version 0.1

Morris Scott (he/him) DOB: 03/03/1955 (67years) Occupation: Retired Plumber Address: 109 Kirribilli Av, Kirribilli NSW 2061 Ph: (02) 2345 6789

Allergies: Penicillin Smoking Hx: Ex-Smoker ATSI: Neither Aboriginal nor Torres Strait Islander

Warnings: Noncompliant with treatment Recalls

Progress note – 14/06/2022

Mr. Scott spirometry (13/06/2022) shows a decrease in FEV1 (45%) compared to previous one (-12%) (12/02/2015). It currently qualifies as severe COPD following GOLD criteria.

Blood test shows a slight decrease of haemoglobin (11.4) with increase in MCV and a total cholesterol of 180 (previous 171)

Results from Colorectal Cancer screening were negative

Previous visits

Date	Recorded by:	Reason for contact
**01/21/2021**	**Dr. Smith**	**Urinary Tract Infection**
03/04/2021	Dr. Singh	Sprained ankle
14/07/2021	Dr. Cheng	Sleeping problems
05/10/2021	Mrs. Johnson	Pap Smear

Click “SUM ALL”

Activity Panel

Treatments
Inhaler
**Enalapril 20mg 1-0-1**
Metformin 850mg 1-1-1

Labs, Referrals & Others
Weight(new)
Symptom – Tiredness – update
Diagnoses: Primary Hypothyroid. - update

Select Find Summarize SUM ALL Edit Add ALL Confirm Cancel

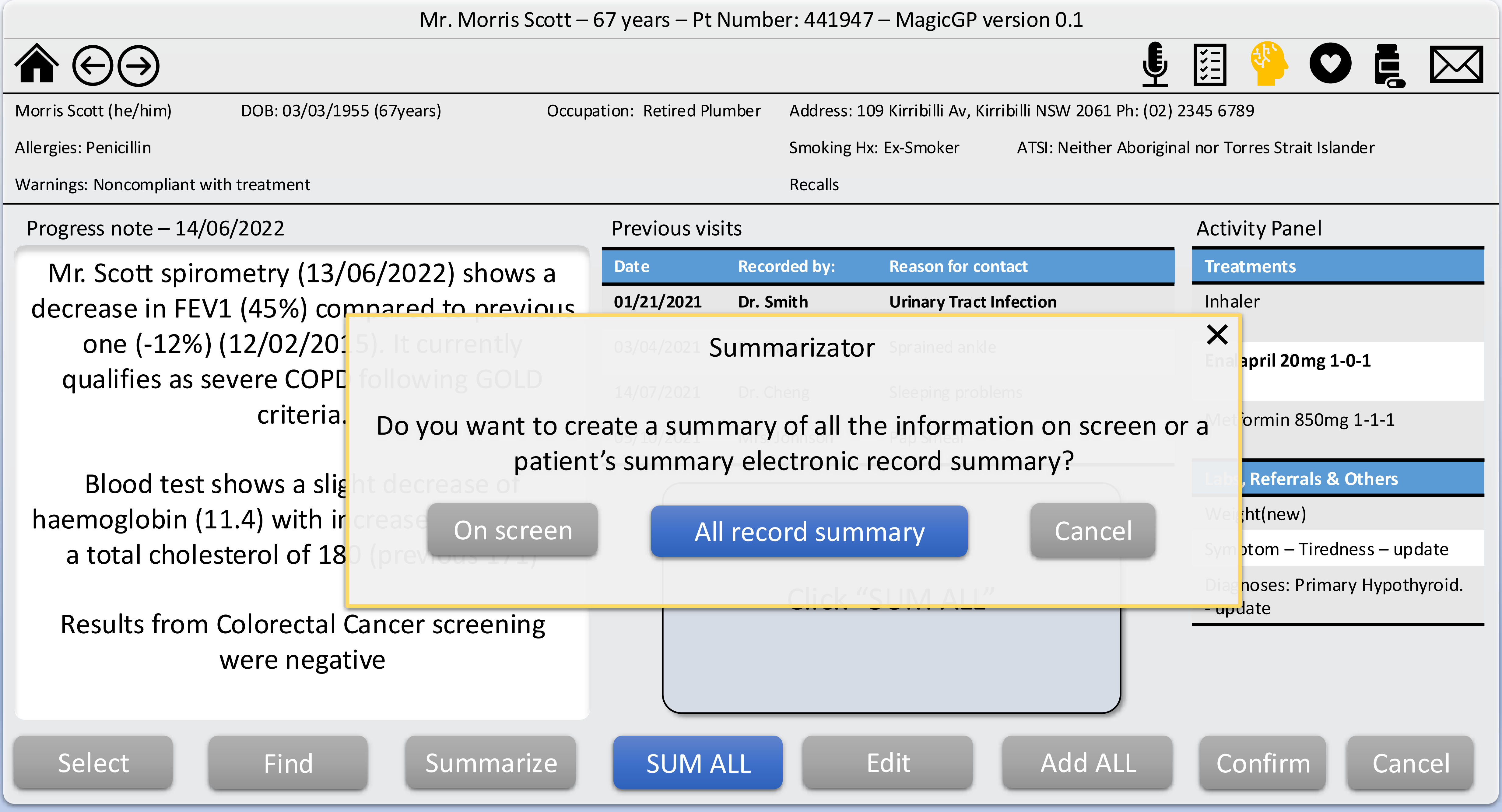
Mr. Morris Scott – 67 years – Pt Number: 441947 – MagicGP version 0.1
Morris Scott (he/him)
DOB: 03/03/1955 (67years)
Occupation: Retired Plumber
Address: 109 Kirribilli Av, Kirribilli NSW 2061 Ph: (02) 2345 6789
Allergies: Penicillin
Smoking Hx: Ex-Smoker
ATSI: Neither Aboriginal nor Torres Strait Islander
Warnings: Noncompliant with treatment
Recalls
Progress note – 14/06/2022
Mr. Scott spirometry (13/06/2022) shows a decrease in FEV1 (45%) compared to previous one (-12%) (12/02/2015). It currently qualifies as severe COPD following GOLD criteria.
Blood test shows a slight decrease of haemoglobin (11.4) with increase a total cholesterol of 180 (previous 171)
Results from Colorectal Cancer screening were negative
Previous visits
Date
Recorded by:
Reason for contact
01/21/2021
Dr. Smith
Urinary Tract Infection
03/04/2021
Sprained ankle
14/07/2021
Dr. Cheng
Sleeping problems
Mrs. Johnson
Pap smear
Click "SUM ALL"
Activity Panel
Treatments
Inhaler
Enalapril 20mg 1-0-1
Metformin 850mg 1-1-1
Labs, Referrals & Others
Weight(new)
Symptom – Tiredness – update
Diagnoses: Primary Hypothyroid. - update
Summarizator
Do you want to create a summary of all the information on screen or a patient's summary electronic record summary?
On screen
All record summary
Cancel
Select
Find
Summarize
SUM ALL
Edit
Add ALL
Confirm
Cancel

Here is the all-record summary letter:

Mr. Morris Scott – 67 years – Pt Number: 441947 – MagicGP version 0.1

Sydney, 14th June 2022 (automated summary generated by MAGIC GP 0.1)

Sydney CBD – GP Clinic
Dr. Alexander Fleming GP

To whom it may concern:

I reviewed Mr. Scott records today. It is important that you are aware he is **allergic to penicillin**. He is a 67-year-old patient and currently its main concerns are a worsening COPD, hypertension and a type 2 Diabetes. He currently receives the following treatments: Symbicort Turbuhaler 200/g twice daily, Enalapril 20mg twice daily and Metformin 850 mg three times a day and Spiriva Respimat once daily that was started today.

In his past visits he has been complaining of more tiredness which may improve with the new inhaler (Spiriva).

His past medical history includes 2 admissions to hospital in the past 3 years due to COPD exacerbations. He had one surgery (appendicectomy) when he was 22 years old.

He is allergic to penicillin and has also the following minor episodes through the past year including a minor ear infection, and cellulitis on one finger after a cut.

Your sincerely,
Dr. Fleming

Switch to tabular view | Edit | Copy | Print | Back

Here is the all-record summary letter:

Mr. Morris Scott – 67 years – Pt Number: 441947 – MagicGP version 0.1

Sydney, 14th June 2022 (automated summary generated by MAGIC GP 0.1)

Sydney CBD – GP Clinic
Dr. Alexander Fleming GP

To whom it may concern:

I reviewed Mr. Scott records today. It is important that you are aware he is **allergic to penicillin**. He is a 67-year-old patient and currently its main concerns are a worsening COPD, hypertension and a type 2 Diabetes. He currently receives the following treatments: Symbicort Turbuhaler 200/g twice daily, Enalapril 20mg twice daily and Metformin 850 mg three times a day and Spiriva Respimat once dai[illegible]

New summary letter has been sent to printer

In his past visits he has been complaining of more tiredness which may improve with the new inhaler (Spiriva).

His past medical history includes 2 admissions to hospital in the past 3 years due to COPD exacerbations. He had one surgery (appendicectomy) when he was 22 years old.

He is allergic to penicillin and has also the following minor episodes through the past year including a minor ear infection, and cellulitis on one finger after a cut.

Your sincerely,
Dr. Fleming

Switch to tabular view | Edit | Copy | Print | Back

Here is the all-record summary letter:

Mr. Morris Scott – 67 years – Pt Number: 441947 – MagicGP version 0.1

Sydney, 14th June 2022 (automated summary generated by MAGIC GP 0.1)

Sydney CBD – GP Clinic
Dr. Alexander Fleming GP

To whom it may concern:

I reviewed Mr. Scott records today. It is imp [illegible] ic to penicillin. He is a 67-year-old patient and currently its main concerns are a worse [illegible] 2 Diabetes. He currently receives the following treatments: Symbicort Turbuhaler [illegible] ice daily and Metformin 850 mg three times a day and Spiriva Respimat once dai [illegible]

In his past visits he has been complaining o [illegible] with the new inhaler (Spiriva).

His past medical history includes 2 admissio [illegible] to COPD exacerbations. He had one surgery (appendicectomy) when he was 22 years old.

He is allergic to penicillin and has also the following minor episodes through the past year including a minor ear infection, and cellulitis on one finger after a cut.

Your sincerely,
Dr. Fleming

×

N [illegible] printer

Excellent! we are done with our second scenario, one more to go!

Switch to tabular view | Edit | Copy | Print | Back

Third Scenario

You are visiting Mrs. Spears, she just moved recently but has already made several visits with various complains and complex needs. Your consultation is very full today and as you are visiting the patient the nurse called you because someone passed out in the waiting room.

Mrs Brittany Spears– 40 years – Pt Number: 771801 – MagicGP version 0.1

Brittany Spears DOB: 02/12/1981 (40 years) Occupation: Musician Address: 1 Barangaroo Ave, Barangaroo NSW 2000

Allergies: Penicillin Smoking Hx: Smoker ATSI: Neither Aboriginal nor Torres Strait Islander

Warnings: Temporary Resident Recalls

Progress note – 14/06/2022

Mrs. Spears came today to discuss her men state after her recent split up with her boyfriend she also wants to rev

Previous visits

contact

ems

refill

hology visit

Emergency in room 2 !

Activity Panel

Treatments

Mirtazapine

Valium

Concerta

Labs, Referrals & Others

Weight(new)

Symptom – Tiredness – update

Diagnoses: Primary Hypothyroid. - update

Highlight | Extract | Extract ALL | Add to EHR | Edit | Add ALL | Confirm | Cancel

You spent 5 minutes with the other patient which was low on sugar… now back to consultation

Mrs. Brittany Spears– 40 years – Pt Number: 771801 – MagicGP version 0.1

Brittany Spears (she/her) DOB: 02/12/1981 (40 years) Occupation: Musician Address: 1 Barangaroo Ave, Barangaroo NSW 2000

Allergies: Shellfish, lactose intolerance, Smoking Hx: Current Smoker ATSI: Neither Aboriginal nor Torres Strait Islander

Warnings: Temporary Resident Recalls: 1

Progress note – 14/06/2022

Mrs. Spears came today to discuss her men
state after her recent split up with her
boyfriend she also wants to rev

Previous visits

contact

ems

refill

hology visit

Phone call from Ambulance services regarding one of your patients

Activity Panel

Treatments

Mirtazapine

Valium

Concerta

Labs, Referrals & Others

Weight(new)

Symptom – Tiredness – update

Diagnoses: Primary Hypothyroid.
- update

Highlight Extract Extract ALL Add to EHR Edit Add ALL Confirm Cancel

Mrs. Brittany Spears– 40 years – Pt Number: 771801 – MagicGP version 0.1

Brittany Spears (she/her) DOB: 02/12/1981 (40 years) Occupation: Musician Address: 1 Barangaroo Ave, Barangaroo NSW 2000

Allergies: Shellfish, lactose intolerance,

Smoking Hx: Current Smoker ATSI: Neither Aboriginal nor Torres Strait Islander

Warnings: Temporary Resident

Recalls: 1

Progress note – 14/06/2022

Mrs. Spears came today to discuss her men state after her recent split up with her boyfriend she also wants to rev

Previous visits

contact

ems

refill

hology visit

After various interruptions you finished the consultation with Brittany but forgot the initial details and haven't redacted the progress note

Activity Panel

Treatments

Mirtazapine

Valium

Concerta

Labs, Referrals & Others

Weight(new)

Symptom – Tiredness – update

Diagnoses: Primary Hypothyroid.
- update

Highlight | Extract | Extract ALL | Add to EHR | Edit | Add ALL | Confirm | Cancel

Mrs. Brittany Spears– 40 years – Pt Number: 771801 – MagicGP version 0.1

Brittany Spears (she/her) DOB: 02/12/1981 (40 years) o Ave, Barangaroo NSW 2000

Allergies: Shellfish, lactose intolerance, moker ATSI: Neither Aboriginal nor Torres Strait Islander

Warnings: Temporary Resident

Let's activate the speech-to-text record.

Progress note – 14/06/2022

Mrs. Spears came today to discuss her mental state after her recent split up with her boyfriend she also wants to rev

for contact

08/06/2022	Dr. Singh	Sleep problems
25/05/2022	Dr. Cheng	Prescription refill
17/05/2022	Mrs. Johnson	Clinical Psychology visit

Activity Panel

Treatments

Mirtazapine

Valium

Concerta

Labs, Referrals & Others

Weight(new)

Symptom – Tiredness – update

Diagnoses: Primary Hypothyroid. - update

Highlight | Extract | Extract ALL | Add to EHR | Edit | Add ALL | Confirm | Cancel

Mrs. Brittany Spears– 40 years – Pt Number: 771801 – MagicGP version 0.1

Brittany S

Allergies:

Warnings:

Occupation: Musician

Address: 1 Barangaroo Ave, Barangaroo NSW 2000

Smoking Hx: Current Smoker

ATSI: Neither Aboriginal nor Torres Strait Islander

Recalls: 1

Let's activate the speech-to-text record.

Progre

Mrs. Spears came today to discuss her mental state after her recent split up with her boyfriend she also wants to rev

TRANSCRIPT

Brittany: Hi Doc! I'm very upset today
Dr. Fleming: Hi Brittany, please take a sit.
Brittany: I have been very upset the last couple of days and it's very hard to sleep once again, I have been using Valium more than I should, I think.
Dr. Fleming: Oh, I see, what is going on? What has been making you upset?
Brittany: Like you know same as always, but this time it has been definitely worst. You know Jake called me the other day, and you know I'm very stupid, but I took the call.
Dr. Fleming: Sorry, can you remind me who is Jake?
Brittany: He is my boyfriend, but it's complicated like in reality we are separated know more or less.

Activity Panel

Treatments

Mirtazapine

Valium

Concerta

Labs, Referrals & Others

Weight(new)

Symptom – Tiredness – update

Diagnoses: Primary Hypothyroid. - update

Play Audio record | See Transcript | Select | Summarize | Sum ALL | Confirm | Cancel

Mrs. Brittany Spears– 40 years – Pt Number: 771801 – MagicGP version 0.1

Now we are selecting and pasting part of the text into our record.

Brittany Spear[illegible]

Allergies: Shel[illegible]

Warnings: Ten[illegible]

Occupation: Musician

Address: 1 Barangaroo Ave, Barangaroo NSW 2000

Smoking Hx: Current Smoker

ATSI: Neither Aboriginal nor Torres Strait Islander

Recalls: 1

Progress n[illegible]

Mrs. Spears came today to discuss her mental state after her recent split up with her boyfriend she also wants to rev

TRANSCRIPT

Brittany: Hi Doc! I'm very upset today
Dr. Fleming: Hi Brittany, please take a sit.
Brittany: I have been very upset the last couple of days and it's very hard to sleep once again, I have been using Valium more than I should, I think.
Dr. Fleming: Oh, I see, what is going on? What has been making you upset?
Brittany: Like you know same as always, but this time it has been definitely worst. You know Jake called me the other day, and you know I'm very stupid, but I took the call.
Dr. Fleming: Sorry, can you remind me who is Jake?
Brittany: He is my boyfriend, but it's complicated like in reality we are separated know more or less.

Activity Panel

Treatments

Mirtazapine

Valium

Concerta

Labs, Referrals & Others

Weight(new)

Symptom – Tiredness – update

Diagnoses: Primary Hypothyroid.
- update

Play Audio record | See Transcript | Select | Summarize | Sum ALL | Confirm | Cancel

Mrs. Brittany Spears– 40 years – Pt Number: 771801 – MagicGP version 0.1

Brittany Spear

Allergies: Shel

Warnings: Ten

Occupation: Musician

Address: 1 Barangaroo Ave, Barangaroo NSW 2000

Smoking Hx: Current Smoker ATSI: Neither Aboriginal nor Torres Strait Islander

Recalls: 1

Now we are selecting and pasting part of the text into our record.

Progress n

Mrs. Spears came today to discuss her mental state after her recent split up with her boyfriend she also wants to rev

TRANSCRIPT

Brittany: Hi Doc! I'm very upset today
Dr. Fleming: Hi Brittany, please take a sit.
Brittany: I have been very upset the last couple of days and it's very hard to sleep once again, I have been using Valium more than I should, I think.
Dr. Fleming: Oh, I see, what is going on? What has been making you upset?
Brittany: Like you know same as always, but this time it has been definitely worst. You know Jake called me the other day, and you know I'm very stupid, but I took the call.
Dr. Fleming: Sorry, can you remind me who is Jake?
Brittany: He is my boyfriend, but it's complicated like in reality we are separated know more or less.

Activity Panel

Treatments

Mirtazapine

Valium

Concerta

Labs, Referrals & Others

Weight(new)

Symptom – Tiredness – update

Diagnoses: Primary Hypothyroid. - update

Play Audio record | See Transcript | Select | Summarize | Sum ALL | Copy | Paste

Mrs. Brittany Spears– 40 years – Pt Number: 771801 – MagicGP version 0.1

Now we are selecting and pasting part of the text into our record.

Brittany Spear

Allergies: Shel

Warnings: Tem

Occupation: Musician

Address: 1 Barangaroo Ave, Barangaroo NSW 2000

Smoking Hx: Current Smoker

ATSI: Neither Aboriginal nor Torres Strait Islander

Recalls: 1

Progress n

Mrs. Spears came today to discuss her mental state after her recent split up with her boyfriend she also wants to re**view her medication. She states:**

"Brittany: I have been very upset the last couple of days and it's very hard to sleep once again, I have been using Valium more than I should, I think."

TRANSCRIPT

Brittany: Hi Doc! I'm very upset today
Dr. Fleming: Hi Brittany, please take a sit.
Brittany: I have been very upset the last couple of days and it's very hard to sleep once again, I have been using Valium more than I should, I think.
Dr. Fleming: Oh, I see, what is going on? What has been making you upset?
Brittany: Like you know same as always, but this time it has been definitely worst. You know Jake called me the other day, and you know I'm very stupid, but I took the call.
Dr. Fleming: Sorry, can you remind me who is Jake?
Brittany: He is my boyfriend, but it's complicated like in reality we are separated know more or less.

Activity Panel

Treatments

Mirtazapine

Valium

Concerta

Labs, Referrals & Others

Weight(new)

Symptom – Tiredness – update

Diagnoses: Primary Hypothyroid. - update

Play Audio record | See Transcript | Select | Summarize | Sum ALL | Copy | Paste

Mrs. Brittany Spears– 40 years – Pt Number: 771801 – MagicGP version 0.1

Brittany Spear[illegible] Occupation: Musician Address: 1 Barangaroo Ave, Barangaroo NSW 2000

Allergies: Shel[illegible] Smoking Hx: Current Smoker ATSI: Neither Aboriginal nor Torres Strait Islander

Warnings: Tem[illegible] Recalls: 1

Now instead we are selecting a larger chunk of text and hitting summarize

Progress n[illegible]

Mrs. Spears came today to discuss her mental state after her recent split up with her boyfriend she also wants to rev**iew her medication. She states:**

"Brittany: I have been very upset the last couple of days and it's very hard to sleep once again, I have been using Valium more than I should, I think."

TRANSCRIPT

Brittany: Hi Doc! I'm very upset today
Dr. Fleming: Hi Brittany, please take a sit.
Brittany: I have been very upset the last couple of days and it's very hard to sleep once again, I have been using Valium more than I should, I think.
Dr. Fleming: Oh, I see, what is going on? What has been making you upset?
Brittany: Like you know same as always, but this time it has been definitely worst. You know Jake called me the other day, and you know I'm very stupid, but I took the call.
Dr. Fleming: Sorry, can you remind me who is Jake?
Brittany: He is my boyfriend, but it's complicated like in reality we are separated know more or less.

Activity Panel

Treatments

Mirtazapine

Valium

Concerta

Labs, Referrals & Others

Weight(new)

Symptom – Tiredness – update

Diagnoses: Primary Hypothyroid. - update

Play Audio record | See Transcript | Select | Summarize | Sum ALL | Copy | Paste

Mrs. Brittany Spears– 40 years – Pt Number: 771801 – MagicGP version 0.1

Now instead we are selecting a larger chunk of text and hitting summarize

Brittany Spear[illegible] Occupation: Musician Address: 1 Barangaroo Ave, Barangaroo NSW 2000

Allergies: Shel[illegible] Smoking Hx: Current Smoker ATSI: Neither Aboriginal nor Torres Strait Islander

Warnings: Tem[illegible] Recalls: 1

Progress n[illegible]

Mrs. Spears came today to discuss her mental state after her recent split up with her boyfriend she also wants to review **her medication. She states:**

"Brittany: I have been very upset the last couple of days and it's very hard to sleep once again, I have been using Valium more than I should, I think."

TRANSCRIPT

Brittany: Hi Doc! I'm very upset today
Dr. Fleming: Hi Brittany, please take a sit.
Brittany: I have been very upset the last couple of days and it's very hard to sleep once again, I have been using Valium more than I should, I think.
Dr. Fleming: Oh, I see, what is going on? What has been making you upset?
Brittany: Like you know same as always, but this time it has been definitely worst. You know Jake called me the other day, and you know I'm very stupid, but I took the call.
Dr. Fleming: Sorry, can you remind me who is Jake?
Brittany: He is my boyfriend, but it's complicated like in reality we are separated know more or less.

Activity Panel

Treatments

Mirtazapine

Valium

Concerta

Labs, Referrals & Others

Weight(new)

Symptom – Tiredness – update

Diagnoses: Primary Hypothyroid. - update

Play Audio record | See Transcript | Select | Summarize | Sum ALL | Copy | Paste

Mrs. Brittany Spears– 40 years – Pt Number: 771801 – MagicGP version 0.1

Brittany Spears (she/her) DOB: 02/12/1981 (40 years) Occupation: Musician Address: 1 Barangaroo Ave, Barangaroo NSW 2000

Allergies: Shellfish, lactose intolerance, Smoking Hx: Current Smoker ATSI: Neither Aboriginal nor Torres Strait Islander

Warning: [illegible] Recalls: 1

Progr[illegible]

Mrs [illegible] state after her recent split up with her boyfri[illegible] her medication. She states:

"[illegible] couple of days and it's very hard to sleep once again, I have been using Valium more than I should, I think."

Brittany complains of sleep problems for the last couple of days after discussing with her ex-boyfriend. She has been taking extra valium for that.

Add to progress notes

Copy Cancel

TRANSC[illegible]

Brittan[illegible]
Dr. Fle[illegible]
Brittan[illegible] of days a[illegible] ve been [illegible]
Dr. Fle[illegible] as been [illegible]
Brittany: Like you know same as always, but this time it has been definitely worst. You know Jake called me the other day, and you know I'm very stupid, but I took the call.
Dr. Fleming: Sorry, can you remind me who is Jake?
Brittany: He is my ex, but it's complicated like in reality we are separated know more or less.

Now let's add the new summarized text to our notes

Activity Panel

Treatments

Mirtazapine

Valium

Concerta

Labs, Referrals & Others

Weight(new)

Symptom – Tiredness – update

Diagnoses: Primary Hypothyroid. - update

Play Audio record | See Transcript | Select | Summarize | Sum ALL | Copy | Paste

Mrs. Brittany Spears– 40 years – Pt Number: 771801 – MagicGP version 0.1

Brittany Spears (she/her) DOB: 02/12/1981 (40 years) Occupation: Musician Address: 1 Barangaroo Ave, Barangaroo NSW 2000

Allergies: Shellfish, lactose intolerance, Smoking Hx: Current Smoker ATSI: Neither Aboriginal nor Torres Strait Islander

Warnings: Temporary Resident Recalls: 1

Progress note – 14/06/2022

Now let's go with our third option. Hit Sum ALL

TRANSCRIPT

Brittany: Hi Doc! I'm very upset today
Dr. Fleming: Hi Brittany, please take a sit.
Brittany: I have been very upset the last couple of days and it's very hard to sleep once again, I have been using Valium more than I should, I think.
Dr. Fleming: Oh, I see, what is going on? What has been making you upset?
Brittany: Like you know same as always, but this time it has been definitely worst. You know Jake called me the other day, and you know I'm very stupid, but I took the call.
Dr. Fleming: Sorry, can you remind me who is Jake?
Brittany: He is my ex, but it's complicated like in reality we are separated know more or less.

Activity Panel

Treatments

Mirtazapine

Valium

Concerta

Labs, Referrals & Others

Weight(new)

Symptom – Tiredness – update

Diagnoses: Primary Hypothyroid. - update

Play Audio record | See Transcript | Select | Summarize | Sum ALL | Copy | Paste

Mrs. Brittany Spears– 40 years – Pt Number: 771801 – MagicGP version 0.1

Brittany Spears (she/her) DOB: 02/12/1981 (40 years) Occupation: Musician Address: 1 Barangaroo Ave, Barangaroo NSW 2000

Allergies: Shellfish, lactose intolerance, Smoking Hx: Current Smoker ATSI: Neither Aboriginal nor Torres Strait Islander

Warnings: Temporary Resident Recalls: 1

Progress note – 14/06/2022

Brittany complains of sleep problems for the last couple of days after discussing with her ex-boyfriend. She has been taking extra valium for that. Her psychiatrist recently adjusted her medication (Mirtazapine, Valium and Concerta) but she still has not found any improvement. We discussed her concerns about her new medication, and she will try to decrease doses of Valium progressively. She also trying to cut her smoking, but she is afraid that will make her increase her weight.

Plan: Adjusted Mirtazapine and Valium Dosage, review in 2 weeks.

Add to progress notes | Copy | Cancel

TRANSCRIPT

[illegible]c! I'm very upset today
[illegible]i Brittany, please take a sit.
[illegible]e been very upset the last couple of [illegible]ery hard to sleep once again, I have [illegible]lium more than I should, I think.
[illegible]h, I see, what is going on? What has [illegible]ou upset?
[illegible]ou know same as always, but this [illegible]en definitely worst. You know Jake [illegible] other day, and you know I'm very [illegible]ok the call.
[illegible]orry, can you remind me who is Jake?
[illegible] my ex, but it's complicated like in [illegible] separated know more or less.

Summarize | Sum ALL | Copy | Paste

Activity Panel

Treatments

- Mirtazapine
- Valium
- Concerta

Labs, Referrals & Others

- Weight(new)
- Symptom – Tiredness – update
- Diagnoses: Primary Hypothyroid. - update

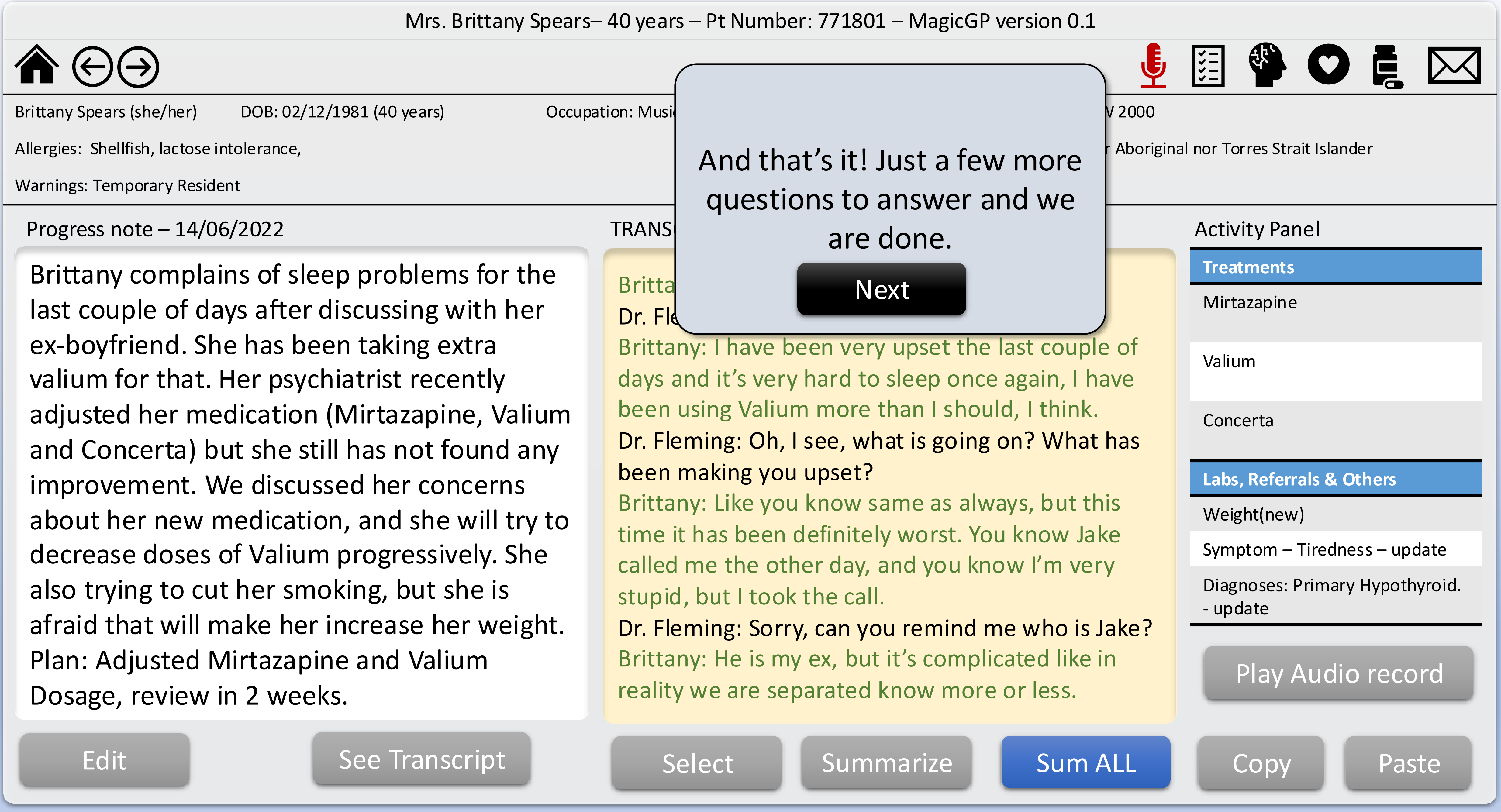
Mrs. Brittany Spears– 40 years – Pt Number: 771801 – MagicGP version 0.1
Brittany Spears (she/her)
DOB: 02/12/1981 (40 years)
Occupation: Musi
V 2000
Allergies: Shellfish, lactose intolerance,
r Aboriginal nor Torres Strait Islander
Warnings: Temporary Resident
Progress note – 14/06/2022
Brittany complains of sleep problems for the last couple of days after discussing with her ex-boyfriend. She has been taking extra valium for that. Her psychiatrist recently adjusted her medication (Mirtazapine, Valium and Concerta) but she still has not found any improvement. We discussed her concerns about her new medication, and she will try to decrease doses of Valium progressively. She also trying to cut her smoking, but she is afraid that will make her increase her weight. Plan: Adjusted Mirtazapine and Valium Dosage, review in 2 weeks.
TRANS
Britta
Dr. Fle
Brittany: I have been very upset the last couple of days and it's very hard to sleep once again, I have been using Valium more than I should, I think.
Dr. Fleming: Oh, I see, what is going on? What has been making you upset?
Brittany: Like you know same as always, but this time it has been definitely worst. You know Jake called me the other day, and you know I'm very stupid, but I took the call.
Dr. Fleming: Sorry, can you remind me who is Jake?
Brittany: He is my ex, but it's complicated like in reality we are separated know more or less.
And that's it! Just a few more questions to answer and we are done.
Next
Activity Panel
Treatments
Mirtazapine
Valium
Concerta
Labs, Referrals & Others
Weight(new)
Symptom – Tiredness – update
Diagnoses: Primary Hypothyroid. - update
Play Audio record
Edit
See Transcript
Select
Summarize
Sum ALL
Copy
Paste
