# Supplementary material for: Understanding Clinician Perceptions of GenAI: A Mixed Methods Analysis of Clinical Documentation Tasks
Source: J Med Syst. 2025 Aug 2;49(1):101. doi: 10.1007/s10916-025-02234-8 (PMC12317912; doi:10.1007/s10916-025-02234-8)
Supplement: Supplementary file 2 — (pdf 123 KB) [file 10916_2025_2234_MOESM2_ESM.pdf]

Final questions

*Required

1. Rank the above 3 scenarios presented according to their relevance to your day-to-day practice. Which one is more important to you? *

Mark only one oval.

- Information Extraction Scenario
- Summarization Scenario
- Speech to text scenario
- All are equally relevant

2. Can you provide a reason for your previous answer?

3. What levels of automation would you be comfortable with, if the above scenarios were deployed in your practice? *

Mark only one oval per row.

	Less Automated	More Automated	Completely Automated	I would not deploy any automation in this scenario
**Scenario 1: Extract Information**	⬭	⬭	⬭	⬭
**Scenario 2: Produce Summaries**	⬭	⬭	⬭	⬭
**Scenario 3: Speech Recognition**	⬭	⬭	⬭	⬭

4. Why did you choose these particular levels of automation for each scenario?

_____

5. Additional comments and feedback:

_____

About you

Tell us more about yourself. This section is optional and you can click the 'Submit' button

below if you are not willing to fill it.

6. Years of primary care experience *

Mark only one oval.

- Less than 5 years
- Between 5 and 10
- Between 10 and 20
- More than 20 years

7. How often do you use and access electronic health records and clinical notes in your day-to-day practice? *

Mark only one oval.

- In every consultation
- Most of the consultations
- Some of the consultations
- Few consultations
- None or almost none of the consultations

8. What context describes your day-to-day activity? You can select more than one option *

Tick all that apply.

- [ ] Primary Care
- [ ] Aged Care facilities
- [ ] Emergency department
- [ ] Home visiting
- [ ] Tele-consultation
- [ ] Other: ____________________

9. Do you work in urban, rural or remote areas? You can select more than one option *

Tick all that apply.

- [ ] Urban
- [ ] Rural
- [ ] Remote

10. How satisfied are you with the current electronic health records systems? *

Mark only one oval.

Very dissatisfied

1 ◯

2 ◯

3 ◯

4 ◯

5 ◯

6 ◯

7 ◯

Completely Satisfied

11. Can you explain your answer to the previous question?

Contact information

If you wish to receive $50 eGift voucher card please provide a valid professional email address below (not gmail, hotmail, etc). We will only use this to send the voucher, and if you opt-in, follow-up information about the study outcomes and/or an interview.

12. Please indicate if you wish to receive information or participate in an interview following this study:

Tick all that apply.

- [ ] I would like to receive $50 eGift voucher
- [ ] I would like to receive information following this study
- [ ] I would like to be invited for an interview in relation to this study

13. Please provide either a valid professional email address (state health department, e.g., nsw.health.org.au, GP Practice email address, RACGP or similar) or a regular email address:

14. If you provided a regular email address in the question above, please provide your name and your main GP practice (E.g. Dr John Smith, Goat Island Practice, Sydney):

This content is neither created nor endorsed by Google.

Google Forms
