# Supplementary material for: Understanding Clinician Perceptions of GenAI: A Mixed Methods Analysis of Clinical Documentation Tasks
Source: J Med Syst. 2025 Aug 2;49(1):101. doi: 10.1007/s10916-025-02234-8 (PMC12317912; doi:10.1007/s10916-025-02234-8)
Supplement: Supplementary file 3 — (pdf 143 KB) [file 10916_2025_2234_MOESM3_ESM.pdf]

```
import pandas as pd

# Replace 'my_excel_file.xlsx' with your actual Excel filename
# and 'Sheet1' with the name of the relevant worksheet.
df = pd.read_excel('magicgp_data.xlsx', sheet_name='Sheet1')

# Export to CSV, without the pandas index column
df.to_csv('magicgp_data.csv', index=False)

import pandas as pd
import numpy as np

from scipy.stats import kruskal, mannwhitneyu, spearmanr,
friedmanchisquare
import pingouin as pg


# --------------------------------------------------------------
-------
# 1) Load the data
# --------------------------------------------------------------
-------
# Adjust the filename to your actual CSV export
df = pd.read_csv("magicgp_data.csv")

# Quick sanity check
print(df.head())
print(df.info())


# --------------------------------------------------------------
-------
# 2) Basic Cleaning / Conversions
# --------------------------------------------------------------
-------
# Ensure numeric columns are truly numeric
```

```python
df['ScenarioMatch'] = pd.to_numeric(df['ScenarioMatch'],
errors='coerce')
df['Importance']    = pd.to_numeric(df['Importance'],
errors='coerce')
df['UseDayToDay']   = pd.to_numeric(df['UseDayToDay'],
errors='coerce')

# Convert categorical columns
df['ImproveWorkflow']    =
df['ImproveWorkflow'].astype('category')
df['PreferredModality']  =
df['PreferredModality'].astype('category')
# "Reasons" can remain text, no special conversion needed
(df['Reasons']).

# Ordinal mapping for safety ranks
# E.g., 1=Safest, 5=Least safe
safety_map = {
    'Safe': 1,
    'Safe with caution': 2,
    'Neither safe nor unsafe': 3,
    'Probably unsafe': 4,
    'Definitely Unsafe': 5
}
df['RankLessAutomated_num'] =
df['RankLessAutomated'].map(safety_map)
df['RankMoreAutomated_num'] =
df['RankMoreAutomated'].map(safety_map)
df['RankCompletelyAutomated_num'] =
df['RankCompletelyAutomated'].map(safety_map)


# --------------------------------------------------------------
-------
# 3) Descriptive Statistics
```

```
# ---------------------------------------------------------------
-------
print("\n--- Descriptive Statistics ---")
num_cols = ['ScenarioMatch', 'Importance', 'UseDayToDay']
print(df[num_cols].describe())

print("\nImproveWorkflow counts:")
print(df['ImproveWorkflow'].value_counts(dropna=False))

print("\nPreferredModality counts:")
print(df['PreferredModality'].value_counts(dropna=False))

# Safety ranks distribution
print("\nSafety Ranks (Less/More/Completely Automated):")
for col in ['RankLessAutomated', 'RankMoreAutomated',
'RankCompletelyAutomated']:
    print(f"{col}:\n{df[col].value_counts(dropna=False)}\n")


# ---------------------------------------------------------------
-------
# 4) Group Comparisons: Kruskal-Wallis
#    Example: test if ScenarioMatch, Importance, UseDayToDay
differ by
#    ImproveWorkflow (Yes/No/Maybe) or by PreferredModality.
# ---------------------------------------------------------------
-------
def kruskal_by_category(data, numeric_col, cat_col):
    """Run Kruskal-Wallis for numeric_col grouped by cat_col."""
    groups = []
    labels = []
    unique_cats = data[cat_col].dropna().unique()

    # Collect data per category
    for cat in unique_cats:
```

```
        cat_data = data.loc[data[cat_col] == cat, 
numeric_col].dropna()
        groups.append(cat_data)
        labels.append(cat)

    # Run test only if at least 2 groups have data
    if len(groups) < 2:
        print(f"Not enough groups to compare for {numeric_col} 
vs {cat_col}")
        return

    stat, p_val = kruskal(*groups)
    print(f"\nKruskal-Wallis: {numeric_col} by {cat_col}")
    print(f" H={stat:.4f}, p={p_val:.4f}, Groups={labels}")

    # If significant, do post-hoc Dunn tests
    if p_val < 0.05:
        print("  --> Post-hoc pairwise (Dunn test) with 
Bonferroni:")
        posthoc = pg.pairwise_tests(
            data=data, dv=numeric_col, between=cat_col,
            parametric=False, padjust='bonf'
        )
        print(posthoc)


# Kruskal-Wallis by ImproveWorkflow
for col in num_cols:
    kruskal_by_category(df, col, 'ImproveWorkflow')

# Kruskal-Wallis by PreferredModality
for col in num_cols:
    kruskal_by_category(df, col, 'PreferredModality')
```

```python
# --------------------------------------------------------------------
-------
# 5) Ordinal Safety Comparisons
#    If you want to see if there's a difference in perceived
safety across
#    Less/More/Completely automated for each participant
(repeated measures),
#    you can use Friedman test (non-parametric repeated-
measures).
#    Each row => one participant's 3 ranks.
# --------------------------------------------------------------------
-------
# We'll skip rows with missing safety data
df_safety = df.dropna(subset=['RankLessAutomated_num',
                              'RankMoreAutomated_num',
                              'RankCompletelyAutomated_num'])

# Gather into arrays or into wide format for Friedman
less_ = df_safety['RankLessAutomated_num']
more_ = df_safety['RankMoreAutomated_num']
comp_ = df_safety['RankCompletelyAutomated_num']

if len(less_) > 2:  # Must have enough data
    stat, pval = friedmanchisquare(less_, more_, comp_)
    print("\nFriedman test for perceived safety (Less vs More vs
Completely automated)")
    print(f"  chi2={stat:.4f}, p={pval:.4f}")
else:
    print("\nNot enough complete data for Friedman test on
safety rankings.")


# --------------------------------------------------------------------
-------
# 6) Correlations among numeric variables
```

```
# ----------------------------------------------------------------
-------
print("\n--- Spearman Correlations among numeric variables ---")
corr_matrix = df[num_cols].corr(method='spearman')
print(corr_matrix)

print("\nPairwise Spearman with p-values:")
for i, c1 in enumerate(num_cols):
    for c2 in num_cols[i+1:]:
        rho, pval = spearmanr(df[c1], df[c2], nan_policy='omit')
        print(f"{c1} vs {c2}: rho={rho:.3f}, p={pval:.4f}")


# ----------------------------------------------------------------
-------
# 7) Example of looking at the distribution of 'Reasons'
#    Because 'Reasons' is free text or multi-option, you might
do
#    simpler frequency checks (e.g., how many times "More
reliable" was
#    mentioned, "More time saved," etc.). This is more of a text
analysis.
# ----------------------------------------------------------------
-------

# A naive approach might search for keywords:
keywords = ["More time saved", "More reliable", "Control over
decisions", "No need to read long texts"]

for kw in keywords:
    df[kw] = df['Reasons'].str.contains(kw, case=False,
na=False)

freq_table = df[keywords].sum().sort_values(ascending=False)
print("\n--- Frequency of Key Reasons ---")
print(freq_table)
```

```
# You can then see how many times each reason was selected among
participants.


# ---------------------------------------------------------------
-----------
# 1) Imports
# ---------------------------------------------------------------
-----------
import pandas as pd
import numpy as np
from scipy.stats import kruskal, mannwhitneyu, spearmanr
import pingouin as pg

# ---------------------------------------------------------------
-----------
# 2) Load the data
# ---------------------------------------------------------------
-----------
# Adjust the path/filename as needed:
df = pd.read_csv("stt_scenario_data.csv")

# Quick check:
print(df.head())
print(df.info())

# ---------------------------------------------------------------
-----------
# 3) Basic Cleaning / Conversions
# ---------------------------------------------------------------
-----------
# Ensure numeric columns are indeed numeric
# (In case CSV loaded them as strings)
df['ScenarioMatch'] = pd.to_numeric(df['ScenarioMatch'],
errors='coerce')
```

```
df['Importance']    = pd.to_numeric(df['Importance'],
errors='coerce')
df['UseDayToDay']   = pd.to_numeric(df['UseDayToDay'],
errors='coerce')

# Sometimes blank or missing cells might appear as NaN
df['ImproveWorkflow'] = df['ImproveWorkflow'].astype('category')
# Yes/No/Maybe

# Drop rows with missing numeric data if necessary:
df_clean = df.dropna(subset=['ScenarioMatch', 'Importance',
'UseDayToDay'])

# --------------------------------------------------------------
-----------
# 4) Descriptive Statistics
# --------------------------------------------------------------
-----------
print("\n--- Descriptive Statistics ---")
print("ScenarioMatch:")
print(df_clean['ScenarioMatch'].describe())

print("\nImportance:")
print(df_clean['Importance'].describe())

print("\nUseDayToDay:")
print(df_clean['UseDayToDay'].describe())

# Count of each category in ImproveWorkflow:
print("\nImproveWorkflow counts:")
print(df_clean['ImproveWorkflow'].value_counts())

# --------------------------------------------------------------
-----------
# 5) Group Comparisons
```

```
#    e.g., Do ScenarioMatch, Importance, or UseDayToDay differ
by
#    whether participants answered Yes/No/Maybe to
ImproveWorkflow?
# ---------------------------------------------------------------
-----------
# Because we have three groups (Yes/No/Maybe), a non-parametric
approach
# like Kruskal-Wallis is appropriate.

# We'll do this for each numeric column: ScenarioMatch,
Importance, UseDayToDay

def kruskal_by_category(df, numeric_col, cat_col):
    """Run Kruskal-Wallis test for the numeric_col grouped by
cat_col
       (which should have 2+ categories). Return summary."""
    groups = []
    unique_cats = df[cat_col].dropna().unique()
    for cat in unique_cats:
        data_group = df.loc[df[cat_col] == cat,
numeric_col].dropna()
        groups.append(data_group)

    # If there are fewer than 2 groups with data, skip
    if len(groups) < 2:
        print(f"Not enough groups for {numeric_col}.")
        return

    stat, p_val = kruskal(*groups)
    print(f"\nKruskal-Wallis: {numeric_col} by {cat_col}")
    print(f" H={stat:.4f}, p={p_val:.4f},
Groups={list(unique_cats)}")

    # If significant, do post-hoc pairwise (Dunn test) with
Pingouin
```

```
    if p_val < 0.05:
        print("  --> Post-hoc pairwise comparisons (Dunn
test):")
        posthoc = pg.pairwise_tests(
            data=df, dv=numeric_col, between=cat_col,
            padjust='bonf', parametric=False
        )
        print(posthoc)


# Run Kruskal-Wallis for each numeric variable
kruskal_by_category(df_clean, 'ScenarioMatch',
'ImproveWorkflow')
kruskal_by_category(df_clean, 'Importance',
'ImproveWorkflow')
kruskal_by_category(df_clean, 'UseDayToDay',
'ImproveWorkflow')

# ----------------------------------------------------------------
-----------
# 6) Pairwise Yes vs. No, Yes vs. Maybe, etc. (if needed)
# ----------------------------------------------------------------
-----------
# If the Kruskal-Wallis is significant, you can do more direct
pairwise
# tests. The Pingouin approach above does Dunn’s test for all
pairs,
# so that might be enough. Alternatively, you can do separate
Mann-Whitney:

"""
for combo in [('Yes','No'), ('Yes','Maybe'), ('No','Maybe')]:
    g1 = df_clean[df_clean['ImproveWorkflow'] ==
combo[0]]['UseDayToDay'].dropna()
    g2 = df_clean[df_clean['ImproveWorkflow'] ==
combo[1]]['UseDayToDay'].dropna()
```

```
    if len(g1) > 0 and len(g2) > 0:
        stat, p_val = mannwhitneyu(g1, g2, alternative='two-
sided')
        print(f"Mann-Whitney: UseDayToDay ~ {combo[0]} vs
{combo[1]}, "
              f"U={stat:.4f}, p={p_val:.4f}")
"""

# ---------------------------------------------------------------
-----------
# 7) Correlations
#    Example: Do ScenarioMatch, Importance, and UseDayToDay
correlate?
# ---------------------------------------------------------------
-----------
print("\n--- Spearman Correlations among numeric variables ---")

cols = ['ScenarioMatch', 'Importance', 'UseDayToDay']
corr_results = df_clean[cols].corr(method='spearman')
print(corr_results)

# Alternatively, do pairwise with p-values:
for c1 in cols:
    for c2 in cols:
        if c1 < c2:  # avoid duplicates
            rho, pval = spearmanr(df_clean[c1], df_clean[c2])
            print(f"{c1} vs {c2}: rho={rho:.3f}, p={pval:.4f}")


import pandas as pd
import numpy as np
from scipy.stats import kruskal, mannwhitneyu, spearmanr
import pingouin as pg

# ---------------------------------------------------------------
------
```

```
# 1) Load the combined data
# ----------------------------------------------------------------
------
# Replace 'combined_data.csv' with the filename that includes
both
# the old columns (ScenarioMatch, Importance, etc.) and the new
columns
# (EHRsatisfaction, YearsExperience, etc.).
df = pd.read_csv("magicgp_data.csv")

# Quick check
print(df.head())
print(df.info())

# ----------------------------------------------------------------
------
# 2) Basic Cleaning / Conversions
# ----------------------------------------------------------------
------
# Ensure numeric columns are truly numeric
numeric_cols = ['ScenarioMatch', 'Importance', 'UseDayToDay',
'EHRsatisfaction']
for col in numeric_cols:
    df[col] = pd.to_numeric(df[col], errors='coerce')

# Convert some columns to categorical if needed
df['ImproveWorkflow'] = df['ImproveWorkflow'].astype('category')
# "Yes", "No", "Maybe"
df['UrbanOrRural']    = df['UrbanOrRural'].astype('category')
# "Urban", "Rural", "Urban, Rural", etc.

# For years of experience, you might keep it as a string or
convert to ordered categories:
# e.g. "Less than 5 years" < "Between 5 and 10" < "Between 10
and 20" < "More than 20 years"
exp_order = [
```

```
    "Less than 5 years",
    "Between 5 and 10",
    "Between 10 and 20",
    "More than 20 years"
]
df['YearsExperience'] = pd.Categorical(df['YearsExperience'],
categories=exp_order, ordered=True)

# Drop rows with critical missing data if needed
df_clean =
df.dropna(subset=['ScenarioMatch','Importance','UseDayToDay','EH
Rsatisfaction'])

# --------------------------------------------------------------
------
# 3) Descriptive Stats & Frequencies
# --------------------------------------------------------------
------
print("\n-- Descriptive Statistics for Key Numeric Columns --")
print(df_clean[numeric_cols].describe())

print("\n-- Distribution of UrbanOrRural --")
print(df_clean['UrbanOrRural'].value_counts())

print("\n-- Distribution of YearsExperience --")
print(df_clean['YearsExperience'].value_counts())

# --------------------------------------------------------------
------
# 4) Example Analysis A: Correlations with EHR Satisfaction
# --------------------------------------------------------------
------
# Spearman correlation between EHRsatisfaction and other numeric
columns
corr_cols =
['ScenarioMatch','Importance','UseDayToDay','EHRsatisfaction']
```

```
corr_matrix = df_clean[corr_cols].corr(method='spearman')
print("\n-- Spearman Correlation Matrix --")
print(corr_matrix)

# Pairwise with p-values
print("\n-- Pairwise Spearman Correlations (with p-values) --")
for i, c1 in enumerate(corr_cols):
    for c2 in corr_cols[i+1:]:
        rho, pval = spearmanr(df_clean[c1], df_clean[c2],
nan_policy='omit')
        print(f"{c1} vs {c2}: rho={rho:.3f}, p={pval:.4f}")

# Interpretation:
# - This can tell you if participants who are more (or less)
satisfied
#   with EHRs also have higher (or lower) "UseDayToDay" scores,
etc.

# ---------------------------------------------------------------
------
# 5) Example Analysis B: Does EHR Satisfaction differ by
Urban/Rural?
# ---------------------------------------------------------------
------
# Because "UrbanOrRural" might have multiple categories
("Urban", "Rural", "Urban, Rural"),
# you can do a Kruskal-Wallis or group them into 2 if it makes
sense (Urban vs. Rural).
grouped_data = []
labels = df_clean['UrbanOrRural'].unique().tolist()
for cat in labels:
    cat_data = df_clean.loc[df_clean['UrbanOrRural'] == cat,
'EHRsatisfaction'].dropna()
    if len(cat_data) > 0:
        grouped_data.append(cat_data)
```

```
if len(grouped_data) > 1:
    stat, p_val = kruskal(*grouped_data)
    print(f"\nKruskal-Wallis EHRsatisfaction by UrbanOrRural:
H={stat:.4f}, p={p_val:.4f}")
    # If significant, do post-hoc
    if p_val < 0.05:
        posthoc = pg.pairwise_tests(
            data=df_clean, dv='EHRsatisfaction',
between='UrbanOrRural',
            parametric=False, padjust='bonf'
        )
        print("\nPost-hoc Dunn test for EHRsatisfaction ~
UrbanOrRural:")
        print(posthoc)

# ----------------------------------------------------------------
------
# 6) Example Analysis C: Does EHR Satisfaction differ by
YearsExperience?
# ----------------------------------------------------------------
------
# YearsExperience is ordinal (4 categories). We can do Kruskal-
Wallis
exp_groups = []
exp_labels = df_clean['YearsExperience'].dropna().unique()
for cat in exp_labels:
    cat_data = df_clean.loc[df_clean['YearsExperience'] == cat,
'EHRsatisfaction'].dropna()
    exp_groups.append(cat_data)

if len(exp_groups) > 1:
    stat, p_val = kruskal(*exp_groups)
    print(f"\nKruskal-Wallis EHRsatisfaction by YearsExperience:
H={stat:.4f}, p={p_val:.4f}")
    if p_val < 0.05:
        posthoc = pg.pairwise_tests(
```

```
            data=df_clean, dv='EHRsatisfaction',
between='YearsExperience',
            parametric=False, padjust='bonf'
        )
        print("\nPost-hoc Dunn test for EHRsatisfaction ~
YearsExperience:")
        print(posthoc)

# ---------------------------------------------------------------
------
# 7) Example Analysis D: Relationship between "Preferred
Automation"
#    levels and "EHRsatisfaction" or "UseDayToDay"
# ---------------------------------------------------------------
------
# Suppose you have columns for each scenario's "preferred
automation,"
# e.g. `AutomationScenario1`, `AutomationScenario2`,
`AutomationScenario3`.
# Each is a category: ["Less Automated","More
Automated","Completely Automated","I would not deploy", ...]
# We can do a Kruskal-Wallis again, grouping by the chosen
category.

scenarios = ['AutomationScenario1', 'AutomationScenario2',
'AutomationScenario3']
for sc_col in scenarios:
    if sc_col in df_clean.columns:
        # E.g., see if "UseDayToDay" differs by chosen
automation approach for scenario 1
        groups = []
        cat_labels = df_clean[sc_col].dropna().unique()
        for cat in cat_labels:
            grp_data = df_clean.loc[df_clean[sc_col] == cat,
'UseDayToDay'].dropna()
            groups.append(grp_data)
```

```
        if len(groups) > 1:
            stat, p_val = kruskal(*groups)
            print(f"\nKruskal-Wallis UseDayToDay by {sc_col}:
H={stat:.4f}, p={p_val:.4f}")
            if p_val < 0.05:
                posthoc = pg.pairwise_tests(
                    data=df_clean, dv='UseDayToDay',
between=sc_col,
                    parametric=False, padjust='bonf'
                )
                print(f"\nPost-hoc Dunn test for UseDayToDay ~
{sc_col}:")
                print(posthoc)

# -------------------------------------------------------------
------
# 8) Summarize or export results
# -------------------------------------------------------------
------
# Optionally, you could export partial results to CSV or a text
file.
# For instance, to export the correlation matrix:
# corr_matrix.to_csv("correlation_matrix.csv")

import pandas as pd
import numpy as np
from scipy.stats import spearmanr, kruskal
import pingouin as pg

# -------------------------------------------------------------
-----------
# 1) Load the data
# -------------------------------------------------------------
-----------
# Replace 'my_data.csv' with your actual CSV file name/path
df = pd.read_csv('magicgp_data.csv')
```

```
# Quick check of data
print(df.head())
print(df.info())

# ---------------------------------------------------------------
-----------
# 2) Spearman Correlations:
#    EHRsatisfaction (numeric) vs ScenarioMatch, Importance,
UseDayToDay
# ---------------------------------------------------------------
-----------
numeric_vars = ["ScenarioMatch", "Importance", "UseDayToDay"]
for var in numeric_vars:
    # Drop rows missing either EHRsatisfaction or the variable
of interest
    valid_data = df.dropna(subset=["EHRsatisfaction", var])

    # Spearman correlation
    rho, p_val = spearmanr(valid_data["EHRsatisfaction"],
valid_data[var], nan_policy='omit')
    print(f"\nEHRsatisfaction vs. {var}:")
    print(f"  Spearman rho = {rho:.3f}, p = {p_val:.4f}")

# ---------------------------------------------------------------
-----------
# 3) Kruskal–Wallis:
#    EHRsatisfaction ~ ImproveWorkflow (categorical: e.g.
"Yes","No","Maybe")
# ---------------------------------------------------------------
-----------
if "ImproveWorkflow" in df.columns:
    # Drop rows missing EHRsatisfaction or ImproveWorkflow
    df_kw = df.dropna(subset=["EHRsatisfaction",
"ImproveWorkflow"])
```

```
    # Create groups for Kruskal-Wallis
    groups = []
    categories = df_kw["ImproveWorkflow"].unique()
    for cat in categories:
        cat_data = df_kw.loc[df_kw["ImproveWorkflow"] == cat,
"EHRsatisfaction"]
        groups.append(cat_data)

    if len(groups) > 1:  # Ensure at least 2 groups
        stat, p_val = kruskal(*groups)
        print(f"\nKruskal–Wallis: EHRsatisfaction by
ImproveWorkflow")
        print(f"  H={stat:.3f}, p={p_val:.4f}")

        # If significant, run post-hoc Dunn test
        if p_val < 0.05:
            posthoc = pg.pairwise_tests(
                data=df_kw,
                dv='EHRsatisfaction',
                between='ImproveWorkflow',
                padjust='bonf',
                parametric=False
            )
            print("\nPost-hoc (Dunn) test results:")
            print(posthoc)
    else:
        print("\nNot enough categories in ImproveWorkflow to run
Kruskal–Wallis.")
else:
    print("\nColumn 'ImproveWorkflow' not found or not needed.")
```
